# Analysis the Burden of Breast Cancer Among Adolescents and Young Adults Using the Global Burden of Disease 2021

**DOI:** 10.1245/s10434-024-16648-0

**Published:** 2024-12-12

**Authors:** Min Zhang, Linlin Yuan, Meimei Cui, Jiayi Chen, Jingjing Jia, Ming Zhao, Dan Zhou, Liling Zhu, Limei Luo

**Affiliations:** 1 Maternal and Child Health Development Research Center, Shandong Provincial Maternal and Child Health Care Hospital Affiliated to Qingdao University, Jinan, Shandong China; 2 School of Public Health, Jiamusi University, Jiamusi, Heilongjiang China; 3 Department of Science and Education, Shandong Provincial Maternal and Child Health Care Hospital Affiliated to Qingdao University, Qingdao, Shandong, China; 4 Department of Clinical Pathology, School of Basic Medicine, Shandong Second Medical University, Weifang, Shandong China; 5 School of Second Clinical Medicine (Yantai Affiliated Hospital), Binzhou Medical University, Yantai, Shandong China; 6 School of Basic Medical, Jiamusi University, Jiamusi, Heilongjiang China; 7 National Office for Maternal and Child Health Surveillance of China, National Center for Birth Defect Surveillance of China, Department of Pediatrics, West China Second University Hospital, Sichuan University, Chengdu, China

**Keywords:** Breast cancer, Adolescents and young adults, Age-standardized incidence rate, Disability-adjusted life years, Estimated annual percentage changes

## Abstract

**Background:**

Unique features and worse outcomes have been reported for breast cancers among adolescents and young adults (AYAs; age 15–39 years). However, there are few up-to-date and comprehensive data to analyze the disease burden of AYA breast cancer.

**Patients and Methods:**

The data of incidence, deaths, and disability-adjusted life years (DALYs) owing to breast cancer was obtained from the Global Burden of Disease (GBD) 2021. We computed estimated annual percentage changes (EAPCs) of each indicator to capture the secular trend in breast cancer and conducted a decomposition analysis to examine factors behind DALYs changes. We also predicted the incident cases, deaths and DALYs to 2044.

**Results:**

From 1990 to 2021, the age-standardized incidence rate (ASIR) experienced an increasing trend globally [EAPC: 0.87, 95% confidence interval (CI) 0.77–0.97]. The age-standardized rates (ASRs) of male AYAs breast cancer were all on the rise. The most significant increase trends in ASRs among female AYAs occurred in North Africa and Middle East, while male AYAs showed the highest increases in East Asia. Population growth contributed the most to the growth of DALYs in East Asia. Frontier analysis showed that despite limited resources, some underdeveloped countries still exhibit superior performance, while other countries with higher sociodemographic index have great room for improvement.

**Conclusions:**

The global burden of AYAs breast cancer is grim, especially in North Africa and Middle East. The significant increase in male AYAs breast cancer burden, targeted prevention strategies may need to be developed for AYAs breast cancer by sex and countries.

**Supplementary Information:**

The online version contains supplementary material available at 10.1245/s10434-024-16648-0.

Breast cancer is considered a major health threat globally, ranking as the leading cause of cancer incidence in females, with over 2.31 million new cases diagnosed in 2022.^1^ A concerning trend is the steady rise in the incidence of breast cancer among younger individuals.^2^ Younger age is an independent predictor of a more unfavorable prognosis.^3^ The diagnosis of breast cancer can induce higher levels of psychological and emotional distress in younger individuals.^4^ Breast cancer in younger individuals typically exhibits a more aggressive phenotype, characterized by a poor prognosis, and a higher proportion of high-grade and later-stage tumors.^5^ Moreover, it remains the most prevalent cause of cancer-related mortality among males worldwide.^6^ Although breast cancer in young males is rare, their survival rate is much lower than that of young females with the same condition, and their prognosis is less optimistic.^7^ Moreover, the factors that increase the risk of breast cancer in male are comparable but not identical to those in females. Thus, a tailored prevention strategy was warranted for males.

There are some studies on the burden of breast cancer currently. A study based on the Global Burden of Disease (GBD) 2019 uncovered a rise in the incidence, deaths and disability-adjusted life years (DALYs) of breast cancer across all ages globally from 1990 to 2019.^8^ Specific focus in other studies on regions, such as developing countries in Asia and Africa, has highlighted an escalating burden of breast cancer across all age groups.^9,10^ Nevertheless, there exists a crucial gap in analyzing the burden of breast cancer among adolescents and young adults (AYAs). AYAs represent a heterogeneous population composed of individuals aged 15–39 years, recognizing that cancer diagnoses coincide with a developmental period from adolescence to adulthood, marked by significant life milestones, such as independent living, workforce entry, and relationship establishment.^4^ This transitional stage often witnesses the adoption of behavior detrimental to their health, including smoking, high-pressure jobs, and elevated consumption of red meat, all contributing to the development of breast cancer.^11^ Additionally, AYAs females with breast cancer have unique concerns about fertility, pregnancy, and contraception, and report difficulty in obtaining information in this regard.^12^

Therefore, we utilized age-standardized incidence rate (ASIR), age-standardized mortality rate (ASMR), age-standardized DALYs rate (ASDR), age-standardized years lived with disability rate (YLDs), and age-standardized years of life lost rate (YLLs) and its temporal trends among AYAs of both sexes from 1990 to 2021. This analysis was conducted at global, regional, and national levels, aiming to identify areas for improvement and potential opportunities to reduce the burden of breast cancer on AYAs.

## Patients and Methods

### Data Source

The GBD 2021 provides a standardized method for estimating mortality and incidence rates by cause and age on the global scale.^13^ The data of breast cancer comes from vital registration, verbal autopsy, police records, surveillance, surveys/censuses, and other health-related data sources, which can provide some or all information about breast cancer incidence and death.^14^ The AYAs referred to the age range from 15 to 39 years (i.e., 15–19 years as youths, 20–39 as young adults) as per earlier publications using GBD data.^15^ Breast cancer was defined by the International Classification of Diseases (ICD) 10th version codes C50-C50.9, D05-D05.9, D24-D24.9, D48.6, D49.3.

For this study, we collected the data of incidence, death, DALYs, YLDs, and YLLs of breast cancer for patients aged 15–39 between 1990 and 2021 from the global health data exchange query tool (http://ghdx.healthdata.org/gbd-results-tool). YLDs were determined by multiplying the prevalence of each sequela by its weight of disability and adding the clinical morbidity associated with a breast cancer diagnosis. YLLs caused by breast cancer were estimated using global standard life expectancy and number of deaths by age. DALYs for breast cancer were derived as the sum of YLDs and YLLs. Detailed calculation methods were elucidated in the series of articles published by GBD.^16^

### Statistical Analysis

To calculate the percent change in age-standardized rates (ASRs) of breast cancer in AYAs with a 95% confidential interval (CI), we utilized the direct method. Specifically, ASRs were computed by summing up the products of age-specific rates (${\alpha }_{i}$, where ${i}$ denotes the ${i}$ th age class) and the number of persons (or weight) (${w}_{i}$) in the same age subgroup ${i}$ of the chosen reference standard population. The sum was then divided by the sum of the standard population weights, as defined by the following equation.

$$\text{ASR}=\frac{{\sum }_{i=1}^{A}{\alpha }_{iwi}}{{\sum }_{i={1}^{{W}^{i}}}^{A}}\times \text{100,000}$$

We used EAPC and 95% CI to quantify the time trend of ASRs in AYAs with breast cancer from 1990 to 2021. Logarithmic linear regression was employed to calculate the EAPC as follows:

$$y=\alpha +\beta x+\varepsilon$$

$$\text{EAPC}=100\times (\text{exp}\left(\beta \right)-1)$$

The ASR was considered to be in a decreasing trend when EAPC and the 95% CI were < 0. On the contrary, the ASR was deemed to be on the rise when EAPC and the 95% CI were > 0. The 95% uncertainty interval (UI) was calculated by taking the 2.5th and 97.5th values of 1000 draw-level estimates for each quantity of interest.

To gain a better understanding of explanatory factors that drove change in AYAs with breast cancer DALYs between 1990 and 2021, we conducted decomposition analyses by population growth, aging, and epidemiologic changes. For the decomposition analysis, we used the methods developed by Das Gupta.^17^ The methodology involved evaluating the contribution of each factor in isolation, with the other two factors remaining fixed.

To assess the relationship between the burden of breast cancer and development status in different territories, we conducted a frontier analysis to identify the lowest potentially achievable ASDR on the basis of socio-demographic index (SDI) value. The frontier delineates the countries or territories with leading performance (at the frontier pushing the envelope) that have the lowest breast cancer burden for their SDI. Distance from the frontier is termed “effective difference” and represents the gap between the observed burden and the potentially achievable burden of disease in a country or territory given their SDI; this gap could be potentially reduced or eliminated on the basis of the country or territory’s sociodemographic resources.^18^ SDI was defined as geometric average of total fertility, per capita income, and average years of education. The countries and territories were divided into five groups by SDI quintiles (high, high-middle, middle, low-middle, and low SDI regions).^19^

We used the nordpred forecasted the incident cases, deaths, and DALYs in 2044. Functions were available in R Studio and the NORDPRED Package.^20^ All statistical analysis was conducted by R 4.3.3 (R Foundation for Statistical Computing, Vienna, Austria).

## Results

### Global Burden of Breast Cancer Among AYAs

In 2021, the global burden of breast cancer among AYAs was notable, with 180,791.49 (95% UI: 166,125.47–197,396.04) new cases and 42,055.42 (95% UI: 38,187.76–46,379.31) deaths reported, and accounted for 2,484,705.41 (95% UI: 2,241,610–2,742,178.83) DALYs (Table 1, Tables S1-S2). The ASIR of female AYAs with breast cancer increased to 11.73/100,000 (95% CI 10.77–12.82) in 2021, with an EAPC of ASIR was 0.83 (95% CI 0.72–0.94) from 1990 to 2021. Meanwhile, the ASMR, ASDR, and age-standardized YLLs rate among female AYAs with breast cancer showed a stable trend; however, the age-standardized YLDs rate showed an increasing trend (EAPC: 0.82, 95% CI 0.71–0.92). Notably, all the ASRs, of male AYAs breast cancer were on the rise globally from 1990 to 2021 (Tables S1 and S3).

**Table 1:** Breast cancer incidence cases, age-standardized incidence rate, and temporal trends among adolescents and young adults globally and geographic regions in 1990 and 2021

Characteristics	Sex	Number (95% UI)		Age-standardized incidence rate (per 100,000) (95% CI)		EAPC (95% CI)
		1990	2021	1990	2021	1990–2021
Global	Both	88,133.32 (83,002.6–94,286.16)	180,791.49 (166,125.47–197,396.04)	4.40 (4.15–4.71)	5.87 (5.39–6.41)	0.87 (0.77–0.97)
	Male	641.26 (487.37–777.42)	2,078.03 (1,172.46–2,681.48)	0.06 (0.05–0.07)	0.13 (0.08–0.17)	2.91 (2.74–3.08)
	Female	87,492.05 (82,347.62–93,671.54)	178,713.45 (164,258.51–195,324.00)	8.86 (8.34–9.48)	11.73 (10.77–12.82)	0.83 (0.72–0.94)
*SDI*						
High	Both	32,326.34 (31,173.54–33,611.94)	34,383.51 (32,658.57–36,180.61)	8.69 (8.38–9.04)	8.32 (7.90–8.76)	− 0.20 (− 0.30 - − 0.09)
	Male	114.74 (106.13–124.29)	180.83 (163.13–208.73)	0.06 (0.06–0.07)	0.09 (0.08–0.10)	0.89 (0.64–1.15)
	Female	32,211.60 (31,056.44–33,497.41)	342,02.68 (32,476.02–35,997.48)	17.55 (16.92–18.25)	17.16 (16.29–18.06)	− 0.12 (− 0.22 - − 0.02)
High-middle	Both	21,688.52 (19,681.61–23,974.52)	35,386.26 (30,459.52–41,446.30)	4.86 (4.42–5.38)	6.60 (5.67–7.73)	1.07 (0.93–1.22)
	Male	169.39 (122.60–221.21)	528.05 (253.79–730.01)	0.07 (0.05–0.10)	0.20 (0.10–0.28)	3.86 (3.53–4.20)
	Female	21,519.13 (19,507.22–23,809.10)	34,858.21 (29,938.12–40,963.94)	9.82 (8.90–10.86)	13.39 (11.49–15.75)	1.07 (0.91–1.22)
Middle	Both	21,517.08 (19,220.36–24,271.35)	59,553.42 (53,578.56–66,366.66)	3.30 (2.95–3.72)	5.93 (5.33–6.60)	1.87 (1.78–1.96)
	Male	207.17 (121.08–272.09)	832.71 (338.23–1,130.48)	0.06 (0.03–0.08)	0.17 (0.07–0.23)	4.20 (3.89–4.50)
	Female	21,309.91 (18,994.56–24,062.90)	58,720.71 (52,784.00–65,524.45)	6.67 (5.95–7.52)	11.79 (10.60–13.16)	1.80 (1.70–1.9)
Low-middle	Both	92,12.53 (7,884.11–10,757.34)	36,976.30 (31,502.32–43,284.30)	2.37 (2.03–2.76)	4.84 (4.13–5.66)	2.24 (2.18–2.31)
	Male	70.02 (53.13–94.83)	275.56 (180.45–429.46)	0.04 (0.03–0.05)	0.07 (0.05–0.11)	2.43 (2.24–2.62)
	Female	9,142.52 (7,810.31–10,691.25)	36,700.75 (31,233.51–42,964.04)	4.75 (4.07–5.55)	9.62 (8.20–11.24)	2.20 (2.12–2.27)
Low	Both	3,287.09 (2,683.54–3,995.60)	14,341.06 (11,504.69–17,365.02)	2.14 (1.75–2.61)	3.80 (3.06–4.59)	1.80 (1.65–1.95)
	Male	79.52 (57.42–121.68)	259.88 (177.32–444.70)	0.10 (0.08–0.16)	0.14 (0.10–0.24)	0.84 (0.79–0.89)
	Female	3,207.57 (2,607.84–3,923.61)	14,081.18 (11,276.27–17,089.35)	4.11 (3.34–5.03)	7.31 (5.87–8.84)	1.79 (1.65–1.93)
*Region*						
Andean Latin America	Both	352.99 (278.34–453.15)	1,139.15 (825.29–1548.07)	2.73 (2.16–3.49)	4.26 (3.09–5.79)	1.38 (1.20–1.56)
	Male	1.69 (1.01–2.74)	7.09 (3.93–12.05)	0.03 (0.02–0.04)	0.05 (0.03–0.09)	3.31 (2.66–3.96)
	Female	351.29 (276.87–451.55)	1,132.06 (819.81–1,541.07)	5.31 (4.20–6.81)	8.38 (6.07–11.41)	1.40 (1.22–1.59)
Australasia	Both	751.11 (633.51–882.5)	1,009.97 (807.98–1,259.18)	8.82 (7.44–10.37)	8.46 (6.76–10.55)	–0.12 (− 0.28 - 0.04)
	Male	2.77 (1.72–4.28)	5.24 (3.36–7.32)	0.07 (0.04–0.10)	0.09 (0.06–0.12)	0.80 (0.36–1.24)
	Female	748.34 (630.61–879.44)	1,004.73 (802.15–1,253.8)	17.48 (14.73–20.55)	16.63 (13.27–20.77)	− 0.13 (− 0.28–0.02)
Caribbean	Both	695.52 (593.19–811.3)	1,101.55 (856.69–1,416.84)	5.47 (4.67–6.38)	5.97 (4.64–7.67)	0.28 (0.16–0.41)
	Male	2.53 (1.97–3.28)	7.58 (5.65–10.18)	0.04 (0.03–0.05)	0.08 (0.06–0.11)	2.24 (1.71–2.76)
	Female	692.99 (590.73–808.59)	1,093.97 (849.28–1,407.97)	10.57 (9.02–12.33)	11.72 (9.1–15.09)	0.34 (0.21–0.46)
Central Asia	Both	1,436.81 (1,318.98–1,562.98)	1,732.09 (1,460.03–2,072.75)	5.60 (5.14–6.10)	4.25 (3.58–5.08)	− 0.77 (− 0.89– − 0.65)
	Male	1.99 (1.57–2.54)	6.09 (4.77–7.78)	0.01 (0.01–0.02)	0.03 (0.02–0.04)	3.44 (2.68–4.20)
	Female	1,434.82 (1,316.98–1,561.07)	1,726.00 (1,454.21–2,066.36)	11.04 (10.13–12.02)	8.48 (7.15–10.16)	− 0.71 (− 0.85– − 0.58)
Central Europe	Both	2956.15 (2728.88–3211.81)	2,815.17 (2,477.09–3,195.42)	5.67 (5.23–6.16)	6.48 (5.70–7.35)	0.51 (0.35–0.67)
	Male	11.89 (10.01–14.20)	16.56 (13.40–21.01)	0.05 (0.04–0.06)	0.08 (0.06–0.10)	1.48 (1.18–1.77)
	Female	2,944.26 (2,717.21–3,199.78)	2,798.62 (2,459.90–3,177.81)	11.41 (10.52–12.41)	13.19 (11.59–14.98)	0.56 (0.39–0.72)
Central Latin America	Both	2,644.13 (2,464.41–2,832.50)	8,282.26 (6,936.44–9,639.21)	4.67 (4.35–5.00)	8.23 (6.89–9.58)	1.70 (1.54–1.85)
	Male	5.55 (4.93–6.23)	20.22 (17.41–23.56)	0.02 (0.02–0.02)	0.04 (0.04–0.05)	2.00 (1.32–2.69)
	Female	2,638.58 (2,458.94–2,827.05)	8,262.05 (6,916.74–9,617.76)	9.04 (8.42–9.68)	15.86 (13.28–18.47)	1.70 (1.54–1.87)
Central Sub-Saharan Africa	Both	328.12 (215.05–492.28)	1,443.14(985.58–2081.25)	1.97 (1.30–2.95)	3.23 (2.22–4.65)	1.62 (1.34–1.90)
	Male	5.33 (3.01–9.89)	17.74 (9.85–32.80)	0.06 (0.04–0.12)	0.08 (0.04–0.14)	0.69 (0.41–0.97)
	Female	322.78 (209.44–486.86)	1,425.4 (965.85–2061.10)	3.83 (2.49–5.76)	6.34 (4.32–9.15)	1.66 (1.40–1.92)
East Asia	Both	16,636.91 (13,204.73–20,737.41)	35,430.98 (26,599.67–46,158.99)	3.21 (2.55–4.00)	6.01 (4.52–7.83)	2.07 (1.93–2.22)
	Male	239.36 (138.56–336.01)	1,051.16 (392.43–1,469.22)	0.09 (0.05–0.12)	0.37 (0.14–0.51)	6.08 (5.54–6.63)
	Female	16,397.55 (12,955.48–20,522.82)	34,379.82 (25,588.19–45,101.27)	6.55 (5.18–8.20)	12.05 (8.99–15.82)	1.94 (1.78–2.10)
Eastern Europe	Both	5,499.25 (5,113.25–5,925.27)	4,992.68 (4,347.73–5,745.95)	5.66 (5.26–6.10)	5.62 (4.89–6.47)	− 0.22 (− 0.43– − 0.02)
	Male	38.64 (35.58–41.55)	24.11 (21.59–26.96)	0.08 (0.07–0.09)	0.06 (0.05–0.06)	− 1.27 (− 1.81–− 0.71)
	Female	5,460.6 (5,074.56–5,885.26)	4,968.57 (4,322.99–5,721.68)	11.20 (10.40–12.07)	11.22 (9.75–12.92)	− 0.21 (− 0.42 - 0.00)
Eastern Sub-Saharan Africa	Both	1,605.9 (1,241.85–2,035.97)	6,935.63 (5,248.4–9,065.87)	2.85 (2.21–3.61)	4.82 (3.67–6.27)	1.52 (1.32–1.71)
	Male	65.89 (45.44–104.91)	229.57 (135.9–425.69)	0.24 (0.16–0.38)	0.33 (0.19–0.61)	0.92 (0.89–0.95)
	Female	1540.01 (1180.5–1970.19)	6706.05 (5021.9–8834.73)	5.26 (4.04–6.72)	9.00 (6.78–11.81)	1.56 (1.38–1.74)
High-income Asia Pacific	Both	3716.88 (3253.77–4268.5)	4531.64 (3884.78–5207.48)	5.42 (4.74–6.22)	7.59 (6.50–8.73)	1.17 (0.94–1.41)
	Male	4.24 (3.02–5.89)	4.27 (2.97–6.00)	0.01 (0.01–0.02)	0.01 (0.01–0.02)	0.06 (-0.41 – 0.53)
	Female	3,712.63 (3,249.87–4,264.93)	4,527.37 (3,880.83–5,203.15)	10.97 (9.6–12.60)	15.66 (13.41–18.01)	1.24 (1.01–1.46)
High-income North America	Both	15,100.03 (14,455.63–15,751.72)	13,042.57 (12,177.62–13,968.74)	12.06 (11.54–12.58)	9.78 (9.13–10.48)	− 0.77 (− 0.92– − 0.62)
	Male	74.13 (67.8–80.78)	90.25 (83.33–96.77)	0.12 (0.11–0.13)	0.14 (0.13–0.15)	0.07 (− 0.28– 0.41)
	Female	15,025.90 (14,383.97–15,676.88)	12,952.32 (12,086.81–13,879.86)	23.89 (22.87–24.93)	19.39 (18.10–20.78)	− 0.78 (− 0.93– − 0.63)
North Africa and Middle East	Both	3,114.29 (2,565.40–3,801.18)	18,869.76 (15,946.73–22,250.75)	2.83 (2.33–3.46)	6.99 (5.91–8.25)	3.21 (3.10–3.32)
	Male	24.12 (16.25–35.39)	94.31 (63.93–134.56)	0.04 (0.03–0.06)	0.07 (0.05–0.10)	1.83 (1.74–1.91)
	Female	3,090.17 (2,540.29–3,777.70)	18,775.46 (15,864.65–22,149.78)	5.79 (4.76–7.08)	14.69 (12.41–17.34)	3.33 (3.20–3.46)
Oceania	Both	116.43 (79.5–165.7)	306.19 (207.48–448.31)	5.16 (3.54–7.33)	5.84 (3.97–8.53)	0.11 (-0.12–0.34)
	Male	0.27 (0.14–0.49)	0.94 (0.43–1.74)	0.02 (0.01–0.04)	0.04 (0.02–0.07)	1.45 (1.34–1.56)
	Female	116.17 (79.30–165.32)	305.25 (206.82–447.24)	10.57 (7.24–15.01)	11.65 (7.91–17.04)	0.03 (− 0.21–0.26)
South Asia	Both	7,611.22 (6,463.47–8,827.84)	32,711.97 (27,081.18–40,022.96)	1.99 (1.70–2.31)	4.26 (3.53–5.21)	2.49 (2.41–2.57)
	Male	58.5 (43.89–79.63)	226.21 (135.02–323.62)	0.03 (0.02–0.04)	0.06 (0.03–0.08)	2.32 (2.02–2.63)
	Female	7,552.72 (6,406.72–8,772.93)	32,485.77 (26,842.17–39,795.04)	4.10 (3.49–4.76)	8.60 (7.11–10.52)	2.40 (2.31–2.48)
Southeast Asia	Both	5,706.98 (4,548.85–7,235.05)	16,400.58 (13,672.64–19,693.41)	3.35 (2.68–4.23)	5.71 (4.76–6.86)	1.58 (1.45–1.71)
	Male	31.52 (18.81–40.66)	97.03 (45.29–134.95)	0.04 (0.02–0.05)	0.07(0.03–0.09)	2.10 (1.97–2.23)
	Female	5,675.46 (4,516.74–7,202.7)	16,303.55 (13,578.69–19,597.95)	6.59 (5.27–8.33)	11.49 (9.57–13.82)	1.67 (1.54–1.79)
Southern Latin America	Both	942.06 (797.31–1,110.92)	1,640.84 (1,374.31–1,961.96)	5.18 (4.38–6.10)	6.08 (5.09–7.27)	0.67 (0.49–0.85)
	Male	2.97 (2.03–4.26)	6.40 (4.40–8.98)	0.03 (0.02–0.05)	0.05 (0.03–0.07)	1.22 (0.82–1.63)
	Female	939.1 (794.13–1,107.97)	1,634.44 (1,367.89–1,955.61)	10.16 (8.60–11.99)	11.99 (10.03–14.35)	0.68 (0.50–0.86)
Southern Sub-Saharan Africa	Both	830.37 (693.52–992.3)	1,815.62 (1,457.27–2,264.21)	4.66 (3.91–5.56)	5.18 (4.16–6.47)	0.67 (− 0.09–1.43)
	Male	10.82 (7.69–14.07)	25.79 (17.62–38.69)	0.12 (0.09–0.16)	0.15 (0.10–0.22)	0.47 (− 0.07–1.01)
	Female	819.55 (683.34–981.11)	1,789.82 (1,431.39–2,233.73)	8.94 (7.48–10.68)	10.15 (8.12–12.67)	0.83 (0.07–1.59)
Tropical Latin America	Both	2,211.48 (2,033.84–2,413.63)	6,712.43 (6,102.63–7,388.06)	3.87 (3.56–4.22)	6.95 (6.31–7.65)	1.65 (1.52–1.78)
	Male	6.44 (5.63–7.40)	27.86 (24.23–31.90)	0.02 (0.02–0.03)	0.06 (0.05–0.07)	3.48 (2.98–3.98)
	Female	2,205.04 (2,027.55–2,407.08)	6,684.57 (6,074.63–7,360.28)	7.55 (6.94–8.24)	13.59 (12.34–14.97)	1.67 (1.54–1.80)
Western Europe	Both	14,537.93 (13,597.80–15,535.70)	13,678.9 (12,602.91–14,728.83)	9.78 (9.15–10.45)	9.22 (8.49–9.93)	-0.15 (-0.28 - -0.02)
	Male	30.2 (26.02–35.37)	48.32 (40.07–58.51)	0.04 (0.03–0.05)	0.07 (0.05–0.08)	1.20 (0.51–1.89)
	Female	14,507.73 (13,565.96–15,506.82)	13,630.58 (12,554.91–14,679.64)	19.68 (18.40–21.04)	18.47 (17.01–19.90)	-0.18 (-0.31–-0.05)
Western Sub-Saharan Africa	Both	1,338.76 (1,009.36–1,684.57)	6,198.37 (4,137.18–8,818.77)	2.32 (1.75–2.91)	4.02 (2.69–5.71)	1.73 (1.60–1.86)
	Male	22.4 (12.35–37.90)	71.30 (37.65–136.54)	0.08 (0.04–0.13)	0.10 (0.05–0.19)	0.60 (0.46–0.74)
	Female	1,316.36 (995.1–1,658.93)	6,127.07 (4,064.54–8,707.86)	4.58 (3.47–5.75)	7.53 (5.00–10.68)	1.59 (1.45–1.72)

*SDI* socio-demographic index; *EAPC* estimated annual percentage change; *CI* confidence interval; *UI* uncertainty intervall

### SDI Level Burden of Breast Cancer Among AYAs

As for different SDI quintiles, the incident cases among AYAs breast cancer increased from 1990 to 2021, with low-middle SDI quintiles experiencing the most significant upward trend in ASIR (EAPC: 2.24, 95% CI 2.18–2.31). Moreover, the high SDI quintiles had the highest ASIR of female AYAs with breast cancer (17.16/100,000, 95% CI 16.29–18.06), demonstrating a decreasing trend from 1990 to 2021 (EAPC: − 0.12, 95% CI − 0.22 to − 0.02). However, the ASIR of breast cancer in male AYAs exhibited an upward trend in all quintiles (Table 1). From 1990 to 2021, the low-middle and low SDI quintiles exhibited an increasing trend in the ASMR and ASDR of female AYAs with breast cancer. However, except for high SDI quintiles, the ASMR and ASDR of male AYAs showed an upward trend in other SDI quintiles (Tables S1 and S3).

### Regional Burden of Breast Cancer Among AYAs

Among the 21 regions, East Asia and South Asia recorded the highest number of incident cases, deaths, DALYs among AYAs with breast cancer in 2021. Notably, The ASRs in North Africa and the Middle East, as well as South Asia exhibited the most significant upward trend (Table 1, Tables S1–S2).

In terms of ASIR among female AYAs, the highest was in high-income North America (19.39/100,000, 95% CI 18.10–20.78). Oceania exhibited the highest ASMR (5.38/100,000, 95% CI 3.60–7.82) and ASDR (311.39/100,000, 95% CI 208.16–453.91) in 2021. However, for male AYAs with breast cancer, Eastern Sub-Saharan Africa ranked first in ASMR (0.18/100,000, 95% CI 0.10–0.33) and ASDR (10.15/100,000, 95% CI 6.07–18.92). From 1990 to 2021, the most significant increase in ASIR (EAPC: 3.33, 95% CI 3.20–3.46), ASMR (EAPC: 1.36, 95% CI 1.22–1.49), and ASDR (EAPC: 1.48, 95% CI 1.35–1.62) among female AYAs were observed in North Africa and Middle East. Nevertheless, East Asia exhibited the most significant upward trends in ASIR and ASDR among male AYAs (Tables S1 and S3).

### National Burden of Breast Cancer Among AYAs

**Fig. 1:**
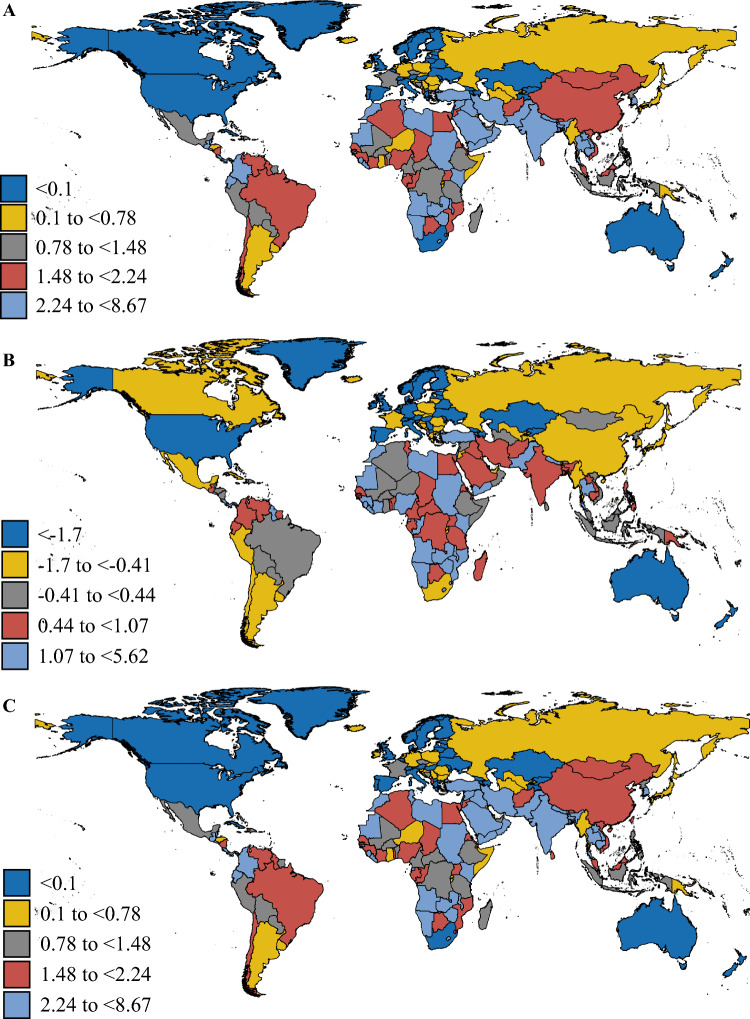
The EAPCs of age-standardized rates of breast cancer in adolescents and young adults between 1990 and 2021 at the national level. **A** Age-standardized incidence rate, **B** Age-standardized mortality rate, and **C** Age-standardized DALYs rate. *DALYs* disability-adjusted life years, *EAPCs* estimated annual percentage changes

In 2021, China and India reported the highest incident cases, deaths, DALYs, YLDs, and YLLs for AYAs breast cancer (Tables S4–S6). The highest ASIR was identified in the Monaco (26.66/100,000, 95% CI 15.36–42.04). From 1990 to 2021, the ASIR increased the most in the Zimbabwe (EAPC: 3.82, 95% CI 2.94–4.71; Table S4 and Fig. 1A). Tokelau had the highest ASMR in 2021, while Zimbabwe showed the most significant upward trend (EAPC: 4.09, 95% CI 3.19–5.00) between 1990 and 2021 (Table S5 and Fig. 1B). As for DALYs, the highest ASDR was observed in the Tokelau (280.76/100,000, 95% CI 178.12–414.27), while the highest increasing trend was observed in the Zimbabwe (EAPC: 4.08, 95% CI 3.18–4.99) from 1990 to 2021 (Table S7 and Fig. 1C).

### Decomposition Analysis of Breast Cancer Disease Burden Among AYAs Globally

**Fig. 2:**
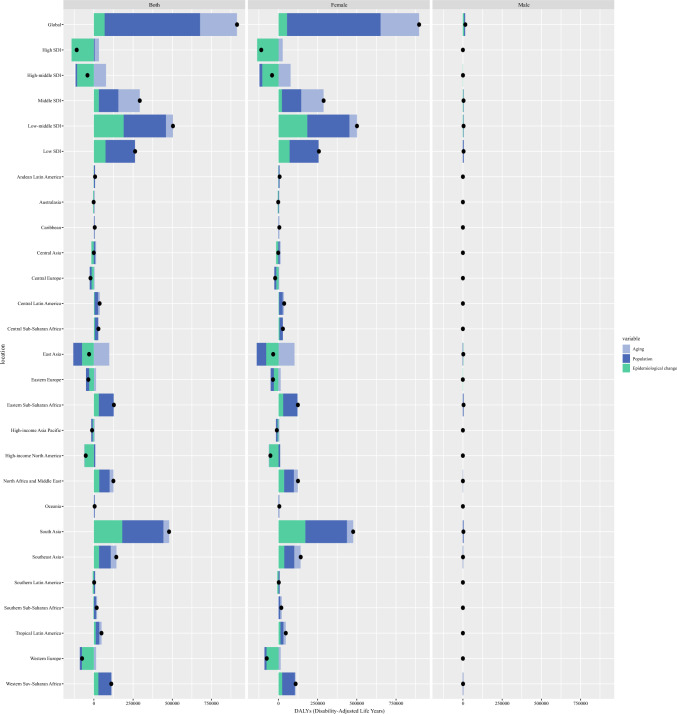
Changes in breast cancer DALYs according to population-level determinants of population growth, aging, and epidemiological change from 1990 to 2021 across location. *DALYs* disability-adjusted life years, *SDI* socio-demographic index

From 1990 to 2021, there was a significant total variation in DALYs among breast cancer in AYAs globally (913,994.98), the growth in DALYs worldwide can be largely attributed to population growth (66.55%) and aging (25.92%; Fig. 2).

Several GBD regions exhibited a decrease in the total variation of DAYLs, but they showed a noticeable increase in epidemiologic changes, including Central Asia (1066.74%), East Asia (241.47%), and Australasia (234.71%). Population growth was the largest contributor to DALYs changes in most regions. South Asia and Southeast Asia exhibited a significant total variation in DALYs, with population growth contributing 54.38% and 50.45% to the change, respectively. In addition, population growth contributed the most to the growth in East Asia (178.24%), population ageing had a considerable contribution to the increased DALYs in Southern Latin America (103.86%) (Table S8 and Fig. 2).

### Frontier Analysis of the Burden of Breast Cancer Among AYAs

**Fig. 3:**
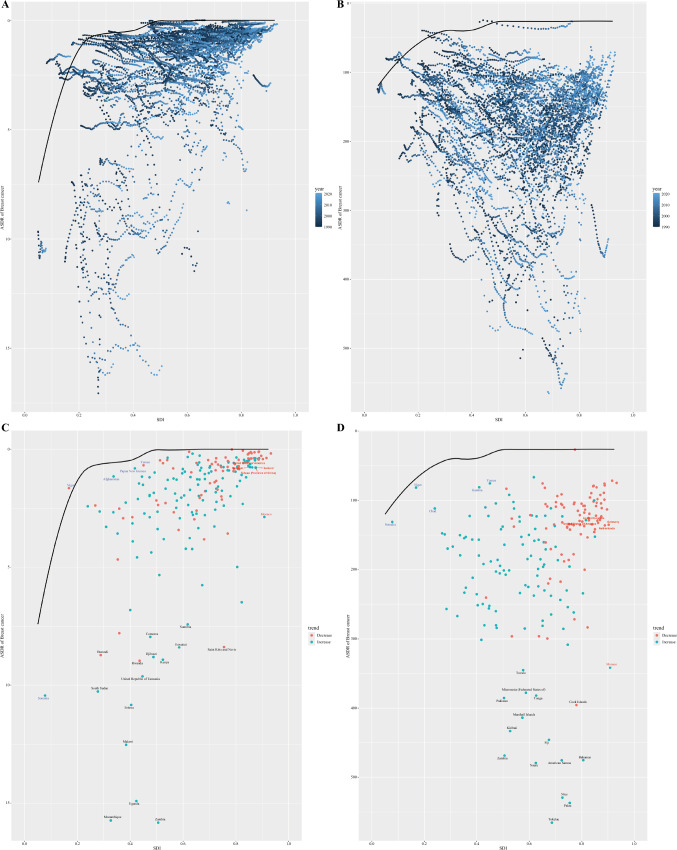
Frontier analysis for identifying improvement gaps for the burden of AYAs breast cancer. The analysis was based on the SDI and ASDR of AYAs breast cancer from 1990 to 2021. **A** Male and **B** Female in 2021. **C** Male and **D** Female. The frontier is delineated in solid black color; countries and territories are represented as dots. The top 15 countries with the largest effective difference (largest breast cancer DALYs gap from the frontier) are labeled in black; examples of frontier countries with low SDI (< 0.5) and low effective difference are labeled in blue, and examples of countries and territories with high SDI (> 0.85) and relatively high effective difference for their level of development are labeled in red. Red dots indicate an increase in ASDR in breast cancer from 1990 to 2021; blue dots indicate a decrease in ASDR in breast cancer between 1990 and 2021. *AYAs* adolescents and young adults, *DALYs* disability-adjusted life years, *SDI* socio-demographic index, *ASDR* age-standardized DALYs rates

We conducted a frontier analysis on the basis of ASDR and SDI to better understand the potential improvement in breast cancer disease burden among AYAs on the basis of SDI of each country (Fig. 3A and B). Among female AYAs, the top ten countries with the highest effective difference in breast cancer were Tokelau, Palau, Niue, Zambia, Nauru, American Samoa, Bahamas, Fiji, Kiribati, and Marshall Islands. Conversely, the five countries with the lowest ASDR and minimal effective differences were Somalia, Niger, Chad, Gambia, and Yemen, all of which have SDI of less than 0.5. In contrast, the five countries with high SDI (> 0.85) and relatively high effective difference for their level of development, namely UK, USA, Germany, the Netherlands, and Monaco (Fig. 3C). For male AYAs with breast cancer, we observed that the effective difference tended to decrease as the SDI increased. The top ten countries with the highest effective difference, considering their stage of development, were Zambia, Mozambique, Uganda, Malawi, Eritrea, South Sudan, United Republic of Tanzania, Rwanda, Kenya, and Djibouti (Fig. 3D).

### Global AYAs Breast Cancer Projections for 2044

**Fig. 4:**
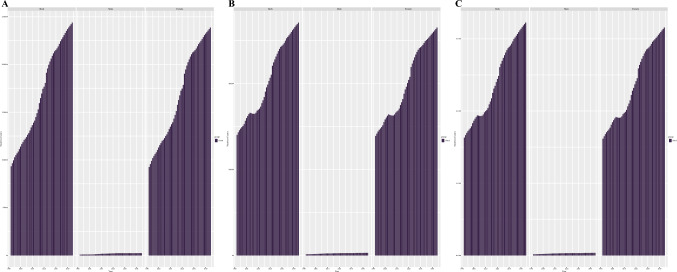
The forecast of AYAs breast cancer to 2044 globally. **A** Incident cases, **B** Deaths, and **C** DALYs. *AYAs* adolescents and young adults, *DALYs* disability-adjusted life years

According to the forecast data, there will be 244,018 incident cases (238,829 females and 2444 males) of breast cancer globally in 2044 (Fig. 4). We observed increasing incident cases in 16 regions from 2021 to 2044, South Asia was projected the region with the fastest-growing (incident cases: 32,711 in 2021 versus 48,318 in 2044). Meanwhile, South Asia (48,318) and Oceania (567) were expected to the highest and lowest incident cases in 2044, respectively. Subsequently, we also projected deaths rising to 54,202 in 2044. By 2044, it is projected that the global DALYs for breast cancer will reach 3,224,966, marking a 29.79% increase compared with 2021. A large increase will be seen in Eastern Sub-Saharan Africa (DALYs: 184,212 in 2021 versus 453,616 in 2044; Table S9).

## Discussion

In this study, we conducted a comprehensive analysis of AYAs breast cancer burden at different levels worldwide and identified improvement gaps using GBD 2021. Meanwhile, we did a decomposition analysis to compute the number of incident cases, deaths and DALYs of AYAs breast cancer worldwide to find the driving factors and made predictions by the year 2044. Our findings are poised to offer etiological insights for breast cancer prevention among AYAs worldwide and elucidate the future health promotion and health resource allocation strategies.

On a global scale, we observed an increase in the number of incident cases, deaths, and DALYs owing to breast cancer among AYAs from 1990 to 2021, and we predicted that it will continue to increase by 2044. Through decomposition analysis found that the increase was driven mainly by population growth and aging. Also, this may be related to the expected increase in breast cancer risk factors, such as high blood glucose levels and high body mass index (BMI).^21^ In addition, the growth trend was attributed to changes in lifestyle (prolonged sitting, dietary changes), and an increase in tobacco and alcohol consumption.^22^ Compared with previous studies, the ASIR trend of AYAs breast cancer was nearly three times higher from 1990 to 2021, this indicated that the disease burden in this age group was gradually increasing. The adoption of western lifestyle, including changes in high energy (caloric) intake, physical activity, and reproductive mode, also promoted the occurrence of AYAs breast cancer.^22^ Notably, the age-standardized YLDs rate of breast cancer in AYAs exhibited an upward trend, this indicated that breast cancer was contributing to an increasing disability burden in AYAs age groups, likely influenced by factors, such as preventive measures, treatment advancements, and improved patient survival.^23^ Particularly, the ASRs trends of breast cancer in male AYAs globally has been on the rise since 1990, which may be related to high estrogen levels, alcohol use, delayed diagnosis and lack of awareness.^22,24^ However, despite the high incidence of hormone receptor positive tumors in male breast cancer, they did not receive chemotherapy or hormone treatment, and tamoxifen has been proven to improve the survival rate of males.^25^ Furthermore, the treatment protocol for male AYAs breast cancer predominantly derives from those for female AYAs breast cancer, no standard of care exists for male breast cancer, and there is an unmet need for research and therapeutic options for this disease.^26^ Thus, there is a compelling need to tailor strategies specifically for males to enhance breast cancer prevention and control efforts.

Of note, the breast cancer burden of AYAs varied in different regions and countries. South Asia, Southeast Asia, and East Asia witnessed the highest numbers among AYAs breast cancer in 2021, our analysis showed that the increase was primarily driven by population growth. Additionally, we found that the growth trend of ASRs of breast cancer in Asia from 1990 to 2021 was more significant than that reported the whole age group.^8^ Especially in South Asia, the growth trend of ASRs was nearly twice as high, the remarkable increase may be attributed to younger females in Asia being more susceptible to development-related risk factors, such as later marriage, delayed childbirth, and shorter period of breastfeeding.^9,10^ Notably, the burden of breast cancer in India’s AYAs was particularly worrying, this phenomenon may be caused by the fact that AYAs in India are more likely to be affected by social and cultural beliefs and lack of screening opportunities.^27,28^ Although ASIR was generally on the rise, ASMR and ASDR in high-income Asia Pacific was on the decline trends, which was contrary to the prior results reported study, the decline was mainly related to regular breast X-ray examinations in high-income Asian countries, such as Japan and South Korea, aimed at detecting breast cancer early, resulting in better survival and lower mortality rate.^29,30^ In addition, the ASRs increase trends of AYAs breast cancer in the North Africa and Middle East from 1990 to 2021 was higher than prior study,^31^ especially in Yemen, Saudi Arabia, and Sudan, where increases may be related to religiosity, social support, and lack of basic healthcare equipment.^32,33^ Contrary to female AYAs breast cancer, male AYAs breast cancer is a rare cancer.^7^ However, we found that the ASRs of male AYAs has increased from 1990 to 2021 globally, mainly in countries located in Central Asia, East Asia, and Tropical Latin America. Most countries have limited medical resources and opportunities for screening and early diagnosis may be delayed, affecting clinical results and leading to an increase in the disease burden of male AYAs breast cancer.^34^ Fortunately, in Eastern Europe, Western Europe, and high-income Asia Pacific, the ASIR of male AYAs breast cancer has declined or remained stable in most countries, this may be related to screening (organized, but also spontaneous), early diagnosis, and timely and appropriate treatment, reflecting the increased attention paid to the burden of breast cancer in AYAs.^35,36^ These results suggested that all regions and countries urgently need to pay more attention to the increasing incidence of AYAs with breast cancer and implement effective preventive measures, including controlling relevant risk factors and carrying out population-based screening programs to reduce the burden of breast cancer.

In frontier analyses, some countries may serve as an example of factors contributing to success, particularly in low-SDI countries (such as Somalia and Niger), which have exhibited superior performance despite the limited resources. Conversely, countries with higher SDI and relatively high effective difference for their level of development, such as Monaco and USA, experienced a more significant breast cancer burden during the study period, necessitating targeted medical policies to ensure proper management of breast cancer.^37^ Additionally, we found that the countries with the largest effective differences in female breast cancer were mainly in Oceania (Tokelau, Palau, Niue, and American Samoa). Sarfati et al. reported that geographic isolation, limited capacity (in terms of personnel skills and infrastructure) and small population were challenges faced in the Pacific region in reducing the burden of cervical cancer.^38^ Contrarily, the highest effective difference in male breast cancer was observed in Eastern Sub-Saharan Africa, particularly in countries, such as Zambia and Mozambique. The low screening rate and lack of professional healthcare personnel in male breast cancer were the main reasons for their lagging performance.^39,40^ Studies further indicate that countries should give priority to adjusting policies and making effective use of resources to mitigate the impact of breast cancer, regardless of their level of development.

However, it is crucial to note several limitations when interpreting our results. Firstly, the data coverage and quality from cancer registries, which significantly affect GBD estimates, are insufficient in many middle and low-income countries. This implies potential bias in breast cancer estimates for these countries. Secondly, the time trend of the ASRs of male AYAs breast cancer should be interpreted with caution. In some countries, the EAPC may be biased owing to the small number of cases of male AYAs breast cancer. Thirdly, owing to the lack of histopathological data, we were unable to further evaluate the burden and trends of specific breast cancer subtypes. Lastly, the SDI is not a metric agreed upon by governments, has not undergone global consultations, and is somewhat affected by assumptions owing to the lack of good country-level data. Therefore, the results should be interpreted with care.

In conclusion, the global burden of AYAs breast cancer is grim, especially in North Africa and Middle East. The significant increase in male breast cancer burden, targeted prevention strategies may need to be developed for AYAs breast cancer by sex and countries. Thus, countries should prioritize adjusting policies and effectively utilizing resources to mitigate the impact of AYAs breast cancer, regardless of their level of development.

## Supplementary Information

Below is the link to the electronic supplementary material.

Supplementary file1 (DOCX 342 kb)
