# Supplementary material for: Analysis the Burden of Breast Cancer Among Adolescents and Young Adults Using the Global Burden of Disease 2021
Source: Ann Surg Oncol. 2024 Dec 12;32(3):2056–69. doi: 10.1245/s10434-024-16648-0 (PMC11811250; doi:10.1245/s10434-024-16648-0)
Supplement: Supplementary file 1 — Supplementary file1 (DOCX 342 kb) [file 10434_2024_16648_MOESM1_ESM.docx]

**Supplementary appendix**

**Table S1**. Breast cancer death cases, age-standardized mortality rate, and temporal trends among adolescents and young adults globally and geographic regions in 1990 and 2021.

**Table S2.** Absolute numbers of DALYs, YLDs and YLLs for breast cancer among adolescents and young adults globally and geographic regions in 1990 and 2021.

**Table S3.** Age-standardized DALYs, YLDs, and YLLs rates for breast cancer among adolescents and young adults globally and geographic regions in 1990 and 2021, as well as time trends.

**Table S4.** The number and age-standardized rate of incidence caused by breast cancer at the national level among adolescents and young adults in 1990 and 2021, as well as time trends.

**Table S5.** The number and age-standardized rate of mortality caused by breast cancer at the national level among adolescents and young adults in 1990 and 2021, as well as time trends

**Table S6.** The numbers of DALYs, YLDs and YLLs for breast cancer among adolescents and young adults at the national level in 1990 and 2021.

**Table S7.** Age-standardized DALYs, YLDs, and YLLs rates for breast cancer among adolescents and young adults at the national level in 1990 and 2021, as well as time trends.

**Table S8.** Percentage of DALYs attributable to risk factors for breast cancer in 2021 globally.

**Table S9.** Incident cases, deaths and DALYs projections of adolescents and young adults breast cancer across different regions in 2044.

**Table S1.** Breast cancer death cases, age-standardized mortality rate, and temporal trends among adolescents and young adults globally and geographic regions in 1990 and 2021.

| **Characteristics** | **Sex** | **Number**  **(95% UI)** | |  | **Age-standardized mortality rate (per 100000) (95% CI)** | |  | **EAPC**  **(95% CI)** |
| --- | --- | --- | --- | --- | --- | --- | --- | --- |
|  |  | **1990** | **2021** |  | **1990** | **2021** |  | **1990-2021** |
| **Global** | Both | 27025.26  (25005.48 to 29516.27) | 42055.42  (38187.76 to 46379.31) |  | 1.35  (1.25 to 1.47) | 1.37  (1.24 to 1.51) |  | -0.06 (-0.18 to 0.05) |
|  | Male | 245.12  (183 to 300.97) | 507.99  (321.37 to 711.95) |  | 0.02  (0.02 to 0.03) | 0.03  (0.02 to 0.05) |  | 1.19 (1.08 to 1.31) |
|  | Female | 26780.14  (24759.54 to 29288.06) | 41547.43  (37729.07 to 45853.42) |  | 2.71  (2.51 to 2.97) | 2.73  (2.47 to 3.01) |  | -0.1 (-0.23 to 0.03) |
| **SDI** |  |  |  |  |  |  |  |  |
| **High** | Both | 5888.13  (5713.09 to 6069.24) | 3880.5  (3706.17to 4061.76) |  | 1.58  (1.53 to 1.63) | 0.93  (0.89 to 0.98) |  | -1.82 (-1.99 to -1.66) |
|  | Male | 17.54  (16.37 to 19.13) | 20.88  (18.47 to 25.19) |  | 0.01  (0.01 to 0.01) | 0.01  (0.01 to 0.01) |  | -0.04 (-0.25 to 0.17) |
|  | Female | 5870.59  (5696.01 to 6051.31) | 3859.61  (3686. to 4040.06) |  | 3.19  (3.1 to 3.29) | 1.92  (1.83 to 2.01) |  | -1.75 (-1.92 to -1.58) |
| **High-middle** | Both | 6378.3  6(5785.1 to 7147.13) | 5497.13  (4854.15 to 6276.29) |  | 1.43  (1.3 to 1.6) | 1.02  (0.9 to 1.16) |  | -1.2 (-1.31 to -1.08) |
|  | Male | 49.33  (36.14 to 62.55) | 60.77  (32.38 to 79.18) |  | 0.02  (0.02 to 0.03) | 0.02  (0.01 to 0.03) |  | 0.32 (0.16 to 0.48) |
|  | Female | 6329.04  (5731.93 to 7098.8) | 5436.36  (4791.79 to 6213.12) |  | 2.89  (2.62 to 3.24) | 2.07  (1.82 to 2.37) |  | -1.19 (-1.32 to -1.06) |
| **Middle** | Both | 8238.21  (7337.96to 9332.81) | 13096.98  (11902.86 to 14391.32) |  | 1.27  (1.13 to 1.44) | 1.3  (1.18 to 1.43) |  | -0.01 (-0.09 to 0.08) |
|  | Male | 81.93  (49.02 to 103.53) | 158.78  (76.42 to 204.82) |  | 0.02  (0.01 to 0.03) | 0.03  (0.02 to 0.04) |  | 1.35 (1.15 to 1.55) |
|  | Female | 8156.28  (7251.05 to 9256.69) | 12938.21  (11736.55 to 14235.8) |  | 2.57  (2.28 to 2.91) | 2.59  (2.35 to 2.85) |  | -0.07 (-0.16 to 0.02) |
| **Low-middle** | Both | 4582.78  (3893.57 to 5394.33) | 13154.42  (11159.17 to 15523.89) |  | 1.18  (1.01 to 1.39) | 1.73  (1.47 to 2.03) |  | 1.14 (1.08 to 1.21) |
|  | Male | 42.9  (32.72 to 58.98) | 127.48  (83.17 to 197.94) |  | 0.02  (0.02 to 0.03) | 0.03  (0.02 to 0.05) |  | 1.47 (1.21 to 1.73) |
|  | Female | 4539.89  (3853.02 to 5355.67) | 13026.93  (11038.46 to 15387.37) |  | 2.37  (2.01 to 2.79) | 3.42  (2.91 to 4.03) |  | 1.09 (1.02 to 1.16) |
| **Low** | Both | 1906.52  (1554.08 to 2317.9) | 6389.05  (5160.46 to 7767.56) |  | 1.25  (1.02 to 1.52) | 1.71  (1.38 to 2.07) |  | 0.94 (0.81 to 1.06) |
|  | Male | 53.25  (38.43 to 80.92) | 139.73  (95.48 to 238.67) |  | 0.07  (0.05 to 0.11) | 0.08  (0.05 to 0.13) |  | 0.11 (0.05 to 0.17) |
|  | Female | 1853.27  (1502.83 to 2266.74) | 6249.32  (5016.34 to 7607.35) |  | 2.39  (1.94 to 2.92) | 3.27  (2.63 to 3.96) |  | 0.93 (0.82 to 1.04) |
| **Region** |  |  |  |  |  |  |  |  |
| **Andean Latin America** | Both | 152.32  (120 to 195.58) | 272.59  (204.08 to 363.01) |  | 1.18  (0.94 to 1.52) | 1.02  (0.77 to 1.36) |  | -0.59 (-0.74 to -0.44) |
|  | Male | 0.94  (0.58 to 1.52) | 2.24  (1.34 to 3.43) |  | 0.01  (0.01 to 0.02) | 0.02  (0.01 to 0.03) |  | 1.26 (0.6 to 1.93) |
|  | Female | 151.38  (119.06 to 194.56) | 270.35  (201.66 to 360.97) |  | 2.3  (1.82 to 2.95) | 2.01  (1.5 to 2.68) |  | -0.58 (-0.73 to -0.42) |
| **Australasia** | Both | 144.8  (124.54 to 165.48) | 105.9  (89.25 to 125.04) |  | 1.7  (1.46 to 1.94) | 0.88  (0.74 to 1.04) |  | -2.31 (-2.45 to -2.18) |
|  | Male | 0.34  (0.27 to 0.43) | 0.46  (0.34 to 0.59) |  | 0.01  (0.01 to 0.01) | 0.01  (0.01 to 0.01) |  | -0.21 (-0.51 to 0.09) |
|  | Female | 144.46  (124.21 to 165.12) | 105.44  (88.78 to 124.54) |  | 3.37  (2.9 to 3.85) | 1.74  (1.46 to 2.05) |  | -2.33 (-2.47 to -2.19) |
| **Caribbean** | Both | 216.26  (181.26 to 265.08) | 313.33  (225.31 to 424.59) |  | 1.71  (1.44 to 2.1) | 1.7  (1.22 to 2.3) |  | 0.1 (-0.07 to 0.26) |
|  | Male | 1.22  (0.94 to 1.63) | 2.84  (2.08 to 3.92) |  | 0.02  (0.02 to 0.03) | 0.03  (0.02 to 0.04) |  | 1.45 (1.07 to 1.84) |
|  | Female | 215.04  (180.02 to 263.92) | 310.49  (222.38 to 421.71) |  | 3.31  (2.77 to 4.05) | 3.33  (2.38 to 4.52) |  | 0.15 (-0.01 to 0.31) |
| **Central Asia** | Both | 513.35  (475.78 to 550.32) | 485.45  (410.33 to 576.68) |  | 2.02  (1.87 to 2.16) | 1.19  (1.01 to 1.41) |  | -1.67 (-1.76 to -1.59) |
|  | Male | 0.72  (0.58 to 0.87) | 1.7  (1.39 to 2.07) |  | 0.01  (0 to 0.01) | 0.01  (0.01 to 0.01) |  | 2.4 (1.72 to 3.08) |
|  | Female | 512.63  (475.11 to 549.57) | 483.75  (408.57 to 574.99) |  | 3.97  (3.68 to 4.26) | 2.38  (2.01 to 2.82) |  | -1.62 (-1.7 to -1.53) |
| **Central Europe** | Both | 835.03  (779.49 to 894.39) | 442.57  (396.19 to 491.91) |  | 1.59  (1.49 to 1.71) | 1.01  (0.9 to 1.12) |  | -1.5 (-1.7 to -1.3) |
|  | Male | 3.35  (2.96 to 3.81) | 2.82  (2.48 to 3.17) |  | 0.01  (0.01 to 0.01) | 0.01  (0.01 to 0.01) |  | -0.21 (-0.42 to 0.01) |
|  | Female | 831.69  (776.24 to 890.97) | 439.76  (393.36 to 488.97) |  | 3.2  (2.99 to 3.43) | 2.05  (1.83 to 2.28) |  | -1.45 (-1.66 to -1.25) |
| **Central Latin America** | Both | 699.63  (662 to 739.49) | 1301.55  (1092.68 to 1501.66) |  | 1.25  (1.18 to 1.32) | 1.29  (1.09 to 1.49) |  | 0.07 (-0.14 to 0.27) |
|  | Male | 2.84  (2.58 to 3.12) | 6.58  (5.85 to 7.38) |  | 0.01  (0.01 to 0.01) | 0.01  (0.01 to 0.02) |  | 0.45 (-0.19 to 1.09) |
|  | Female | 696.78  (659.22 to 736.66) | 1294.97  (1086.42 to 1494.74) |  | 2.41  (2.28 to 2.55) | 2.49  (2.09 to 2.87) |  | 0.07 (-0.15 to 0.29) |
| **Central Sub-Saharan Africa** | Both | 195.57  (129.22 to 289.22) | 685.21  (470.01 to 982.5) |  | 1.18  (0.78 to 1.74) | 1.54  (1.06 to 2.21) |  | 0.88 (0.67 to 1.09) |
|  | Male | 3.56  (2.01 to 6.51) | 9.71  (5.36 to 17.89) |  | 0.04  (0.02 to 0.08) | 0.04  (0.02 to 0.08) |  | 0.03 (-0.16 to 0.23) |
|  | Female | 192.01  (125.65 to 285.92) | 675.5  (460.42 to 972.81) |  | 2.29  (1.5 to 3.4) | 3.02  (2.07 to 4.34) |  | 0.92 (0.74 to 1.11) |
| **East Asia** | Both | 5920.45  (4659.39 to 7412.27) | 5157.05  (3855.71 to 6713.44) |  | 1.14  (0.9 to 1.43) | 0.87  (0.65 to 1.13) |  | -1.16 (-1.3 to -1.01) |
|  | Male | 70.12  (38.87 to 97.25) | 113.1  (41.71 to 156.03) |  | 0.03  (0.01 to 0.04) | 0.04  (0.01 to 0.05) |  | 2.23 (1.85 to 2.6) |
|  | Female | 5850.33  (4589.61 to 7345.73) | 5043.95  (3747.68 to 6592.51) |  | 2.34  (1.84 to 2.94) | 1.75(1.3 to 2.29) |  | -1.26 (-1.42 to -1.09) |
| **Eastern Europe** | Both | 1677.04  (1572.34 to 1784.61) | 1031.92  (889.62 to 1206.24) |  | 1.72  (1.61 to 1.83) | 1.15  (0.99 to 1.34) |  | -1.67 (-1.83 to -1.51) |
|  | Male | 11.86  (11.07 to 12.53) | 4.97  (4.52 to 5.46) |  | 0.02  (0.02 to 0.03) | 0.01  (0.01 to 0.01) |  | -2.78 (-3.38 to -2.17) |
|  | Female | 1665.18  (1560.45 to 1772.67) | 1026.95  (884.48 to 1201.31) |  | 3.4  (3.19 to 3.62) | 2.29  (1.97 to 2.67) |  | -1.66 (-1.81 to -1.51) |
| **Eastern Sub-Saharan Africa** | Both | 950.58  (727.33 to 1214.25) | 3149.31  (2399.86 to 4125.72) |  | 1.7  (1.3 to 2.17) | 2.2  (1.69 to 2.88) |  | 0.67 (0.51 to 0.83) |
|  | Male | 43.71  (30.62 to 69.36) | 122.04  (72.97 to 227.99) |  | 0.16  (0.11 to 0.25) | 0.18  (0.1 to 0.33) |  | 0.19 (0.13 to 0.25) |
|  | Female | 906.86  (686.84 to 1173.9) | 3027.27  (2285.65 to 4000.31) |  | 3.12  (2.36 to 4.03) | 4.1  (3.11 to 5.39) |  | 0.71 (0.56 to 0.86) |
| **High-income Asia Pacific** | Both | 645.54  (596.89 to 696.76) | 433.76  (393.92 to 483.86) |  | 0.94  (0.87 to 1.02) | 0.72  (0.65 to 0.8) |  | -1 (-1.11 to -0.88) |
|  | Male | 1.25  (0.91 to 1.72) | 0.6  (0.51 to 0.75) |  | 0  (0 to 0) | 0  (0 to 0) |  | -2.58 (-2.89 to -2.26) |
|  | Female | 644.28  (595.69 to 695.67) | 433.17  (393.3 to 483.23) |  | 1.9  (1.76 to 2.06) | 1.48  (1.34 to 1.65) |  | -0.94 (-1.04 to -0.83) |
| **High-income North America** | Both | 2342.22  (2268.97 to 2419.59) | 1428.49  (1346.39 to 1508.5) |  | 1.87  (1.81 to 1.93) | 1.07  (1.01 to 1.13) |  | -1.88 (-2.12 to -1.64) |
|  | Male | 6.77  (6.45 to 7.1) | 7.3  (6.9 to 7.72) |  | 0.01  (0.01 to 0.01) | 0.01  (0.01 to 0.01) |  | -0.29 (-0.62 to 0.04) |
|  | Female | 2335.45  (2262.28 to 2412.84) | 1421.18  (1339.19 to 1501.15) |  | 3.71  (3.59 to 3.83) | 2.12  (2 to 2.24) |  | -1.89 (-2.13 to -1.65) |
| **North Africa and Middle East** | Both | 885.83  (730.38 to 1088.77) | 2969.39  (2498.15 to 3538.89) |  | 0.81  (0.67 to 1) | 1.1  (0.92 to 1.31) |  | 1.23 (1.11 to 1.34) |
|  | Male | 12.1  (8.18 to 17.67) | 27.39  (19.18 to 38.15) |  | 0.02  (0.01 to 0.03) | 0.02(0.01 to 0.03) |  | -0.13 (-0.18 to -0.09) |
|  | Female | 873.73  (718.63 to 1076.13) | 2942  (2474.4 to 3508.37) |  | 1.65  (1.36 to 2.04) | 2.3  (1.93 to 2.74) |  | 1.36 (1.22 to 1.49) |
| **Oceania** | Both | 50.86  (34.17 to 71.94) | 141.05  (94.39 to 205.09) |  | 2.27  (1.53 to 3.21) | 2.7  (1.81 to 3.92) |  | 0.51 (0.38 to 0.65) |
|  | Male | 0.16  (0.08 to 0.29) | 0.5  (0.22 to 0.93) |  | 0.01  (0.01 to 0.03) | 0.02  (0.01 to 0.04) |  | 1.08 (0.97 to 1.19) |
|  | Female | 50.71  (34.03 to 71.79) | 140.55  (93.92 to 204.6) |  | 4.65  (3.13 to 6.57) | 5.38  (3.6 to 7.82) |  | 0.43 (0.29 to 0.57) |
| **South Asia** | Both | 4093.84  (3476.22 to 4758.24) | 12177.63  (10044.8 to 14956.01) |  | 1.08  (0.92 to 1.25) | 1.59  (1.31 to 1.95) |  | 1.24 (1.15 to 1.34) |
|  | Male | 36.24  (27.11 to 49.97) | 101.03  (60.97 to 143.6) |  | 0.02  (0.01 to 0.03) | 0.03  (0.02 to 0.04) |  | 1.19 (0.81 to 1.57) |
|  | Female | 4057.6  (3440.67 to 4718.3) | 12076.59  (9943.97 to 14863.77) |  | 2.21  (1.88 to 2.57) | 3.2  (2.64 to 3.93) |  | 1.14 (1.05 to 1.24) |
| **Southeast Asia** | Both | 2599.87  (2041.14 to 3339.13) | 5087.39  (4233.9 to 6117.08) |  | 1.53  (1.21 to 1.96) | 1.77  (1.47 to 2.13) |  | 0.32 (0.19 to 0.44) |
|  | Male | 17.14  (10.32 to 22.05) | 38.1  (17.03 to 53.38) |  | 0.02  (0.01 to 0.02) | 0.03  (0.01 to 0.04) |  | 1.01 (0.83 to 1.2) |
|  | Female | 2582.73  (2023.31 to 3321.68) | 5049.29  (4194.55 to 6074.99) |  | 3.01  (2.37 to 3.86) | 3.56  (2.95 to 4.28) |  | 0.4 (0.27 to 0.52) |
| **Southern Latin America** | Both | 348.19  (298.96 to 405.64) | 371.27  (317.04 to 431.79) |  | 1.92  (1.65 to 2.23) | 1.37  (1.17 to 1.6) |  | -0.87 (-1.1 to -0.64) |
|  | Male | 1.34  (0.99 to 1.79) | 1.78  (1.35 to 2.3) |  | 0.01(0.01 to 0.02) | 0.01  (0.01 to 0.02) |  | -0.39 (-0.79 to 0) |
|  | Female | 346.84  (297.56 to 404.35) | 369.49  (315.31 to 430.03) |  | 3.76  (3.23 to 4.38) | 2.71  (2.31 to 3.15) |  | -0.86 (-1.09 to -0.63) |
| **Southern Sub-Saharan Africa** | Both | 376.77  (315.51 to 446.16) | 704.92  (572.58 to 873.91) |  | 2.13  (1.79 to 2.52) | 2.01  (1.63 to 2.5) |  | 0.18 (-0.59 to 0.96) |
|  | Male | 5.79  (4.08 to 7.37) | 11.34  (7.87 to 16.83) |  | 0.07  (0.05 to 0.08) | 0.07  (0.05 to 0.1) |  | -0.24 (-0.87 to 0.4) |
|  | Female | 370.98  (309.96 to 440.33) | 693.58  (560.5 to 862.72) |  | 4.07  (3.41 to 4.83) | 3.93  (3.18 to 4.89) |  | 0.35 (-0.43 to 1.13) |
| **Tropical Latin America** | Both | 830.24  (768.23 to 897.08) | 1665.74  (1535.7 to 1803.25) |  | 1.46  (1.35 to 1.58) | 1.72  (1.58 to 1.86) |  | 0.33 (0.22 to 0.44) |
|  | Male | 3.26  (2.94 to 3.63) | 9.55  (8.64 to 10.49) |  | 0.01  (0.01 to 0.01) | 0.02  (0.02 to 0.02) |  | 2.1 (1.61 to 2.59) |
|  | Female | 826.98  (765.02 to 893.92) | 1656.19  (1526.27 to 1793.56) |  | 2.84  (2.63 to 3.08) | 3.35  (3.09 to 3.63) |  | 0.34 (0.24 to 0.45) |
| **Western Europe** | Both | 2806.73  (2656.36 to 2959.07) | 1458.03  (1379.08 to 1539.21) |  | 1.89  (1.79 to 1.99) | 0.98  (0.92 to 1.03) |  | -2.18 (-2.34 to -2.03) |
|  | Male | 8.11  (7.3 to 8.99) | 7.17  (6.42 to 7.95) |  | 0.01  (0.01 to 0.01) | 0.01  (0.01 to 0.01) |  | -0.93 (-1.53 to -0.33) |
|  | Female | 2798.62  (2648.46 to 2951.23) | 1450.87  (1371.78 to 1532.27) |  | 3.8  (3.6 to 4.01) | 1.95  (1.84 to 2.06) |  | -2.2 (-2.36 to -2.05) |
| **Western Sub-Saharan Africa** | Both | 740.16  (571.04 to 918.53) | 2672.88  (1807.93 to 3711.93) |  | 1.29  (1 to 1.6) | 1.75  (1.18 to 2.42) |  | 0.92 (0.8 to 1.03) |
|  | Male | 14.31  (7.97 to 23.73) | 36.79  (20.66 to 68.87) |  | 0.05  (0.03 to 0.08) | 0.05  (0.03 to 0.1) |  | -0.12 (-0.25 to 0.01) |
|  | Female | 725.85  (557.32 to 900.84) | 2636.09  (1769.61 to 3668.38) |  | 2.54  (1.96 to 3.14) | 3.26  (2.19 to 4.54) |  | 0.77 (0.66 to 0.89) |

SDI: sociodemographic index; EAPC: estimated annual percentage change; CI: confidence interval; UI: uncertainty interval.

**Table S2.** Absolute numbers of DALYs, YLDs and YLLs in breast cancer among adolescents and young adults in 1990 and 2021.

| Characteristics | Sex | | DALYs (95% UI) | | |  | YLDs (95% UI) | |  | YLLs (95% UI) | |  |
| --- | --- | --- | --- | --- | --- | --- | --- | --- | --- | --- | --- | --- |
|  |  |  | **1990** | | **2021** |  | **1990** | **2021** |  | **1990** | **2021** |  |
| Global | Both | | 1570710.42(1451459.28 to 1718107.07) | | 2484705.41(2241610 to 2742178.83) |  | 62062.25(43142.3 to 84894.92) | 126236.64(87417.94 to 173484.49) |  | 1508648.17(1394531.7 to 1649416.64) | 2358468.76(2135839.05 to 2607334.68) |  |
|  | Male | | 14632.26(10885.49 to 18000.68) | | 30535.9(19189.29 to 42720.77) |  | 495.78(330.63 to 697.81) | 1609.83(870.5 to 2413.37) |  | 14136.49(10473.26 to 17369.18) | 28926.07(18245.25 to 40587.75) |  |
|  | Female | | 1556078.16(1436331.42 to 1703473.96) | | 2454169.51(2215872.29 to 2711727.66) |  | 61566.47(42769.81 to 84186.67) | 8.18(5.66 to 11.24) |  | 1494511.69(1380329.87 to 1636263.52) | 2329542.69(2109876.24 to 2577583.64) |  |
| SDI | |  | |  | | | | | | | |  |
| High | Both | | 347952.61(335147.14to 361828.15) | | 239050.59(226168.09 to 253688.42) |  | 23837.3(16703.2to 32520.19) | 25406(17814.96to34750.46) |  | 324115.3(314429.54to 334155.39) | 213644.59(204020.85 to 223667.01) |  |
|  | Male | | 1084.2(1008.99 to 1181.49) | | 1318.1(1163.18 to 1582.07) |  | 98.3(67.85 to 135.07) | 151.85(104.01 to 210.93) |  | 985.9(919.41 to 1076.21) | 1166.25(1030.93 to 1407.55) |  |
|  | Female | | 346868.41(334091.53 to 360738.99) | | 237732.49(224916.11 to 252239.29) |  | 23739(16634.28 to 32385.55) | 12.69(8.9 to 17.38) |  | 323129.41(313468.94 to 333148.28) | 212478.34(202906.43 to 222452.5) |  |
| High-middle | Both | | 368577.42(333469.86to 412681.58) | | 328152.8(288206.66to 375701.42) |  | 15331.15(10604.8 to 21218.38) | 25234.33(17188.5to 35592.62) |  | 353246.27(320041.11 to 396207.56) | 302918.47(267304.72 to 346151.99) |  |
|  | Male | | 2982.24(2164.24 to 3804.5) | | 3869.45(2037.22 to 5084.26) |  | 132.76(83.9 to 193.39) | 418.1(196.67 to 674.34) |  | 2849.48(2064.95 to 3631.7) | 3451.35(1827.62 to 4504.97) |  |
|  | Female | | 365595.18(330440.27 to 409969.49) | | 324283.35(284132.59 to 371524.11) |  | 15198.39(10507.44 to 21043.97) | 9.54(6.5 to 13.48) |  | 350396.79(316983.01 to 393416.5) | 299467.12(263743.63 to 342568.2) |  |
| Middle | Both | | 475714.72(422931.48to539501.28) | | 769417.34(697413.71 to 849458.94) |  | 14555.12(9977.96 to 20003.86) | 40854.51(28387.89 to 57054.43) |  | 461159.59(410525.83 to 522874.88) | 728562.83(661798.78 to 801158.88) |  |
|  | Male | | 4946.86(2946.9 to 6284.13) | | 9720.91(4644.38 to 12596.26) |  | 156.39(89.62 to 232.67) | 645.42(259.13 to 1017.11) |  | 4790.47(2846.75 to 6066.8) | 9075.49(4355.42 to 11710.77) |  |
|  | Female | | 470767.85(417544.64 to 534628.01) | | 759696.43(687754.35 to 839768.38) |  | 14398.73(9866.47 to 19792.96) | 8.08(5.6 to 11.31) |  | 456369.12(405443.67 to 518415.8) | 719487.34(652323.83 to 792216.05) |  |
| Low-middle | Both | | 266380.31(225576.28to 314169.38) | | 772128.58(651889.79 to 916793.77) |  | 6112.47(4099.76to 8465.07) | 24995.47(16976.42 to 34720.98) |  | 260267.85(220800.37 to 306829.69) | 747133.1(631164.37to 885382.21) |  |
|  | Male | | 2506.72(1911.76 to 3434.43) | | 7440.98(4857.38 to 11563.8) |  | 51.89(34.08 to 77.95) | 204.68(122.92 to 349.49) |  | 2454.83(1870.38 to 3367.65) | 7236.3(4711.48 to 11246.42) |  |
|  | Female | | 263873.59(223143.65to 311707.85) | | 764687.6(644625.87 to 908565.43) |  | 6060.57(4060.77 to 8405.03) | 6.49(4.41 to 9) |  | 257813.02(218471.06 to 304610.23) | 739896.8(624331.06 to 877667.47) |  |
| Low | Both | | 110282.45(89936.77 to 134348.35) | | 373772.82(301581.54 to 455712) |  | 2156.71(1427.31to 3027.89) | 9643.15(6518.23to13913.1) |  | 108125.74(88157.08to 131502.19) | 364129.67(293376.49 to 443657.96) |  |
|  | Male | | 3101.92(2236.21 to 4709.55) | | 8165.69(5583.04 to 13999.32) |  | 56.1(34.84 to 92.06) | 189.02(112.3 to 350.05) |  | 3045.83(2196.02 to 4626.16) | 7976.67(5446.19 to 13670.33) |  |
|  | Female | | 107180.53(86930.2 to 131343.53) | | 365607.13(292723.83 to 446087.17) |  | 2100.61(1386.07 to 2959.58) | 4.89(3.31 to 7.04) |  | 105079.91(85224.95 to 128569.91) | 356153(285228.15 to 434537.35) |  |
| Region | |  | |  | | | | | | | |  |
| Andean Latin America | Both | | 8792.58(6923.65 to 11324.14) | | 15997.11(11924.01 to 21339.42) |  | 221.69(136.9 to 336.3) | 740.77(455.4 to 1149.45) |  | 8570.89(6738.79 to 11032.17) | 15256.34(11409.36 to 20338.59) |  |
|  | Male | | 56.85(34.52 to 91) | | 134.43(80.02 to 206.34) |  | 1.21(0.64 to 2.1) | 5.2(2.74 to 9.4) |  | 55.64(33.78 to 89.35) | 129.23(76.9 to 198.07) |  |
|  | Female | | 8735.72(6871.4 to 11257.26) | | 15862.69(11786.55 to 21203.81) |  | 220.47(135.93 to 334.77) | 5.44(3.35 to 8.46) |  | 8515.25(6683.51 to 10971.34) | 15127.11(11269.99 to 20219.09) |  |
| Australasia | Both | | 8525.35(7341.08 to 9814.86) | | 6561.55(5515.37 to 7770.25) |  | 556.98(353.58 to 807.6) | 754.83(467.83 to 1117.43) |  | 7968.37(6851.78 to 9110.4) | 5806.72(4889.77 to 6864.18) |  |
|  | Male | | 21.12(16.61 to 26.76) | | 29.15(21.95 to 38.27) |  | 2.36(1.28 to 4.02) | 4.41(2.57 to 6.9) |  | 18.77(14.92 to 23.61) | 24.74(18.6 to 32.04) |  |
|  | Female | | 8504.23(7321.48 to 9794.24) | | 6532.4(5486.1 to 7743.66) |  | 554.62(351.87 to 804.5) | 12.44(7.7 to 18.45) |  | 7949.6(6833.65 to 9090.56) | 5781.98(4864.21 to 6837.04) |  |
| Caribbean | Both | | 12542.07(10530.34 to 15387.97) | | 18130.23(13066.43 to 24598.63) |  | 445.23(296.63 to 614.19) | 704.21(436.86 to 1019.69) |  | 12096.84(10130.35 to 14845.98) | 17426.03(12507.7 to 23688.63) |  |
|  | Male | | 71.66(55.22 to 95.46) | | 166.7(122.36 to 228.83) |  | 1.81(1.19 to 2.6) | 5.43(3.59 to 7.97) |  | 69.84(53.85 to 93.33) | 161.27(117.89 to 222.41) |  |
|  | Female | | 12470.41(10456.33 to 15313.4) | | 17963.53(12892.47 to 24434.81) |  | 443.42(295.35 to 611.91) | 7.49(4.64 to 10.85) |  | 12027(10059.54 to 14779.85) | 17264.75(12341.85 to 23523.91) |  |
| Central Asia | Both | | 29658.36(27472.12 to 31775.02) | | 28225.23(23868.2 to 33445.76) |  | 989.68(672.76 to 1392.54) | 1219.21(809.92 to 1758.07) |  | 28668.68(26576.62 to 30729.38) | 27006.02(22811.17 to 32092.6) |  |
|  | Male | | 45.45(37.18 to 55.4) | | 107.35(88.09 to 131.02) |  | 1.67(1.1 to 2.46) | 4.89(3.19 to 7.14) |  | 43.78(35.73 to 53.41) | 102.46(84.09 to 124.9) |  |
|  | Female | | 29612.91(27428.3 to 31728.39) | | 28117.88(23759.56 to 33341.61) |  | 988.01(671.59 to 1390.36) | 5.97(3.96 to 8.61) |  | 28624.9(26535.52 to 30683.97) | 26903.56(22705.45 to 31990.36) |  |
| Central Europe | Both | | 47725.74(44567.42 to 51128) | | 26220.49(23346.3 to 29202.29) |  | 2043.5(1412.43 to 2800.49) | 1981.13(1346.81 to 2751.78) |  | 45682.24(42628.91 to 48949.6) | 24239.36(21694.89 to 26944.73) |  |
|  | Male | | 197.75(175.96 to 225.06) | | 170.71(150.19 to 193.43) |  | 9.61(6.47 to 13.8) | 13.2(8.87 to 18.82) |  | 188.14(166.59 to 214.35) | 157.51(138.86 to 177.34) |  |
|  | Female | | 47527.99(44376.1 to 50931.25) | | 26049.78(23178.42 to 29020) |  | 2033.89(1405 to 2786.85) | 9.29(6.32 to 12.91) |  | 45494.1(42446.02 to 48756.55) | 24081.86(21536.84 to 26779.91) |  |
| Central Latin America | Both | | 40926.7(38748.64 to 43288.75) | | 77690.11(65352.54 to 89714.74) |  | 1689.02(1199.32 to 2297.29) | 5286.36(3637.14 to 7525.71) |  | 39237.69(37120.88 to 41472.88) | 72403.74(60809.78 to 83529.37) |  |
|  | Male | | 170.82(154.3 to 187.36) | | 396.06(351.72 to 443.62) |  | 4.09(2.84 to 5.75) | 14.79(10.24 to 20.48) |  | 166.73(151.02 to 183.18) | 381.27(339.17 to 427.66) |  |
|  | Female | | 40755.88(38575.59 to 43115.6) | | 77294.05(64966.09 to 89299.5) |  | 1684.92(1196.24 to 2292.05) | 10.12(6.96 to 14.41) |  | 39070.96(36957.54 to 41307.32) | 72022.47(60447.07 to 83126.99) |  |
| Central Sub-Saharan Africa | Both | | 11197.57(7399.39 to 16558.72) | | 39455.53(26889.6 to 56626.76) |  | 213.35(115.3 to 354.7) | 966.13(536.8 to 1559.46) |  | 10984.22(7252.15 to 16262.34) | 38489.4(26297.56 to 55275.9) |  |
|  | Male | | 208.12(117.26 to 380.87) | | 567.64(313.11 to 1050.81) |  | 3.8(1.97 to 7.29) | 13.08(6.63 to 26.55) |  | 204.32(115.03 to 373.52) | 554.56(305.02 to 1025.09) |  |
|  | Female | | 10989.45(7187.36 to 16367.58) | | 38887.9(26360.27 to 56151.59) |  | 209.55(111.96 to 350.53) | 4.23(2.35 to 6.84) |  | 10779.9(7049.04 to 16074.4) | 37934.84(25747.4 to 54722.13) |  |
| East Asia | Both | | 341768.02(269246.45 to 428783.06) | | 310971.39(232105.74 to 405083.44) |  | 11596.01(7493.83 to 16662.08) | 25322.53(15849.32 to 38104.09) |  | 330172.01(259807.17 to 413873.07) | 285648.86(213545.7 to 372028.2) |  |
|  | Male | | 4312.28(2364.49 to 6007.37) | | 7289.5(2697.55 to 10086.2) |  | 182.76(99.86 to 282.41) | 824.73(296.7 to 1318.78) |  | 4129.52(2257.98 to 5736.7) | 6464.77(2371.16 to 8922.58) |  |
|  | Female | | 337455.74(265026.67 to 424890.86) | | 303681.89(225317.72 to 397240.54) |  | 11413.25(7371.45 to 16389.89) | 8.6(5.32 to 13) |  | 326042.49(255695.03 to 409904.25) | 279184.09(207343.02 to 365136.43) |  |
| Eastern Europe | Both | | 96035.85(89941.15 to 102455.99) | | 59914.82(51687.12 to 69995.59) |  | 3800.58(2616.51 to 5287.73) | 3502.64(2475.75 to 4858.76) |  | 92235.27(86479.89 to 98172.36) | 56412.17(48624.88 to 65953.97) |  |
|  | Male | | 694.87(648.19 to 734.33) | | 293.1(265.97 to 322.21) |  | 30.81(21.62 to 42.04) | 19.54(13.67 to 27.04) |  | 664.06(620.02 to 701.7) | 273.56(248.87 to 300.63) |  |
|  | Female | | 95340.99(89249.39 to 101776.34) | | 59621.72(51392.35 to 69709.71) |  | 3769.77(2594.77 to 5247.22) | 7.88(5.56 to 10.95) |  | 91571.21(85814.41 to 97504.15) | 56138.61(48342.25 to 65682.23) |  |
| Eastern Sub-Saharan Africa | Both | | 55130.34(42177.68 to 70505.79) | | 184212.99(139665.56 to 241812.55) |  | 1057.37(677.15 to 1537.5) | 4683.39(3027.48 to 7034.64) |  | 54072.97(41347.02 to 69106.31) | 179529.6(136289.79 to 235813.34) |  |
|  | Male | | 2562.55(1797.86 to 4069.71) | | 7159.2(4286.37 to 13391.62) |  | 46.28(27.74 to 77.59) | 167.09(86.78 to 331.72) |  | 2516.27(1765.24 to 3995.91) | 6992.11(4185.08 to 13087.09) |  |
|  | Female | | 52567.79(39784.36 to 68027.43) | | 177053.79(133154.64 to 234093.71) |  | 1011.09(645.75 to 1484.81) | 6.04(3.89 to 9.15) |  | 51556.7(39019.13 to 66771.49) | 172537.49(129786.13 to 228664.25) |  |
| High-income Asia Pacific | Both | | 38411.7(35373.46 to 41722.12) | | 27276.16(24499.72 to 30620.89) |  | 2828.48(1903.11 to 3935.41) | 3436.53(2250.25 to 4898.26) |  | 35583.23(32841.91 to 38469.12) | 23839.62(21646.38 to 26608.02) |  |
|  | Male | | 75.69(55.2 to 102.85) | | 37.49(31.58 to 47.08) |  | 3.99(2.43 to 6.31) | 3.91(2.32 to 6.08) |  | 71.7(52.17 to 98.28) | 33.58(28.46 to 42.03) |  |
|  | Female | | 38336.01(35298.41 to 41637.33) | | 27238.67(24463.37 to 30580.54) |  | 2824.49(1899.66 to 3930.53) | 11.9(7.79 to 16.98) |  | 35511.53(32772.43 to 38406.56) | 23806.04(21611.8 to 26572.74) |  |
| High-income North America | Both | | 140171.66(134390.94 to 146431.6) | | 88536.87(82726.34 to 94812.4) |  | 11090.54(7785.75 to 15068.14) | 9643.07(6791.28 to 13204.45) |  | 129081.12(125014.03 to 133370.68) | 78893.8(74349.55 to 83315.46) |  |
|  | Male | | 443.07(414.05 to 474.88) | | 485.5(449.52 to 527.73) |  | 63.52(44.18 to 87.08) | 77.11(53.11 to 105.34) |  | 379.56(361.2 to 397.89) | 408.39(385.28 to 431.77) |  |
|  | Female | | 139728.59(133958.21 to 145969.73) | | 88051.37(82265.47 to 94315.88) |  | 11027.03(7739.2 to 14984.34) | 14.34(10.1 to 19.64) |  | 128701.56(124638.68 to 132992.91) | 78485.41(73946.35 to 82904.42) |  |
| North Africa and Middle East | Both | | 51667.34(42766.96 to 63481.1) | | 177844.14(149156.35 to 212573.33) |  | 2165.52(1411.47 to 3138.38) | 13013(8718.85 to 18293.5) |  | 49501.82(40807.99 to 60831.89) | 164831.14(138605.7 to 196572.5) |  |
|  | Male | | 724.21(488.79 to 1054.32) | | 1642.72(1146.11 to 2294.23) |  | 18.37(10.99 to 28.67) | 73.96(44.71 to 115.82) |  | 705.85(476.33 to 1030.49) | 1568.76(1095.76 to 2185.02) |  |
|  | Female | | 50943.13(42043.78 to 62804.02) | | 176201.42(147779.73 to 210757.03) |  | 2147.15(1397.58 to 3115.61) | 10.13(6.79 to 14.26) |  | 48795.98(40116.57 to 60095.62) | 163262.38(137251.62 to 194825.13) |  |
| Oceania | Both | | 2967.73(1990.19 to 4214.22) | | 8195.48(5473.97 to 11951.55) |  | 78.57(44.58 to 126.17) | 205.06(112.16 to 343.27) |  | 2889.16(1935 to 4095.18) | 7990.41(5328.97 to 11658.8) |  |
|  | Male | | 8.84(4.44 to 16.44) | | 27.93(12.52 to 52.56) |  | 0.2(0.1 to 0.39) | 0.7(0.31 to 1.4) |  | 8.64(4.34 to 16.08) | 27.23(12.19 to 51.16) |  |
|  | Female | | 2958.89(1982.12 to 4204.46) | | 8167.54(5449.87 to 11927.57) |  | 78.37(44.45 to 125.88) | 7.78(4.25 to 13.04) |  | 2880.52(1927.48 to 4086.71) | 7963.18(5303.39 to 11631.39) |  |
| South Asia | Both | | 238196.07(202057.79 to 277412.09) | | 718573.95(588958.89 to 884821.32) |  | 5076.22(3449.32 to 7002.71) | 22361.66(14554.81 to 31956.47) |  | 233119.85(197604.76 to 271424.33) | 696212.28(571795.35 to 858818.33) |  |
|  | Male | | 2101.41(1576.35 to 2891.46) | | 5853.61(3516.43 to 8353.84) |  | 44.29(28.45 to 65.75) | 169.64(96.06 to 272.59) |  | 2057.13(1539.46 to 2831.24) | 5683.98(3426.42 to 8089.57) |  |
|  | Female | | 236094.65(199984.15 to 275207.6) | | 712720.33(583981.67 to 879460.78) |  | 5031.93(3417.96 to 6949) | 5.87(3.82 to 8.38) |  | 231062.72(195582.69 to 269173.83) | 690528.31(566115.57 to 853636.56) |  |
| Southeast Asia | Both | | 149709.2(117077.26 to 192815.02) | | 293944.93(243946.2 to 352785.56) |  | 3853.08(2398.58 to 5623.42) | 11319.37(7598.15 to 16009.02) |  | 145856.12(114150.5 to 187857.34) | 282625.56(235081.73 to 340115.04) |  |
|  | Male | | 1034.22(620.76 to 1334.14) | | 2294.93(1017.64 to 3224.47) |  | 23.77(14.08 to 34.84) | 73.95(34.56 to 117.76) |  | 1010.44(606.16 to 1301.84) | 2220.98(986.04 to 3111.24) |  |
|  | Female | | 148674.98(115993.33 to 191804.15) | | 291650(241606.86 to 350757.99) |  | 3829.3(2382.4 to 5596.27) | 7.93(5.32 to 11.21) |  | 144845.67(113100.18 to 186830.73) | 280404.58(232802.68 to 337662.65) |  |
| Southern Latin America | Both | | 19947.25(17161.88 to 23252.64) | | 21827.34(18501.59 to 25541.92) |  | 684.02(427.41 to 1025.76) | 1227.36(759.12 to 1837.55) |  | 19263.23(16532.9 to 22453.64) | 20599.98(17571.72 to 23994.08) |  |
|  | Male | | 79.33(58.32 to 105.39) | | 107.43(81.42 to 138.66) |  | 2.44(1.44 to 3.86) | 5.4(3.22 to 8.63) |  | 76.89(56.44 to 102.53) | 102.02(77.34 to 131.75) |  |
|  | Female | | 19867.92(17082.79 to 23178.05) | | 21719.91(18391.61 to 25436.82) |  | 681.58(425.6 to 1022.39) | 8.97(5.54 to 13.43) |  | 19186.34(16452.31 to 22379.22) | 20497.96(17472.24 to 23893.21) |  |
| Southern Sub-Saharan Africa | Both | | 21733.81(18190.69 to 25803.88) | | 40201.53(32592.53 to 50049.47) |  | 557.49(375.1 to 796.92) | 1220.91(819.41 to 1735.29) |  | 21176.32(17704.4 to 25101.91) | 38980.62(31589.91 to 48457.77) |  |
|  | Male | | 338.69(238.78 to 434.04) | | 665.08(461.78 to 985.78) |  | 7.98(5.06 to 11.83) | 19.51(12.02 to 31.94) |  | 330.71(233.06 to 424.04) | 645.56(447.08 to 958.31) |  |
|  | Female | | 21395.12(17835.15 to 25474.57) | | 39536.46(31887.86 to 49346.94) |  | 549.51(368.96 to 787.26) | 6.81(4.55 to 9.7) |  | 20845.61(17387.01 to 24769.31) | 38335.06(30910.2 to 47818.05) |  |
| Tropical Latin America | Both | | 47840.68(44213.12 to 51730.4) | | 96443.77(88948.27 to 104653.07) |  | 1408.13(965.42 to 1926.59) | 4262.05(2974.44 to 5767.9) |  | 46432.55(42964.33 to 50173.36) | 92181.72(84956.44 to 99811.31) |  |
|  | Male | | 195.39(175.5 to 217.15) | | 571.17(516.16 to 629.83) |  | 4.88(3.3 to 6.88) | 20.31(14.22 to 28.29) |  | 190.51(171.37 to 212) | 550.86(498.09 to 605.38) |  |
|  | Female | | 47645.29(44015.81 to 51533.8) | | 95872.6(88375.21 to 104081.26) |  | 1403.26(961.94 to 1919.9) | 8.64(6.03 to 11.71) |  | 46242.04(42777.46 to 49989.21) | 91630.86(84411.44 to 99250.72) |  |
| Western Europe | Both | | 165333.9(156221.18 to 174843.7) | | 90284.17(84010.5 to 96984.94) |  | 10829.64(7433.41 to 15027.84) | 10254.95(6882.03 to 14275.33) |  | 154504.26(146244.57 to 162910.43) | 80029.22(75656.53 to 84523.78) |  |
|  | Male | | 480.36(432.35 to 531.37) | | 438.44(391.52 to 493.91) |  | 26.11(17.59 to 36.66) | 41.24(27.72 to 58.35) |  | 454.25(409.05 to 503.49) | 397.2(355.87 to 441.23) |  |
|  | Female | | 164853.55(155753.42 to 174369.68) | | 89845.73(83571.32 to 96539.54) |  | 10803.53(7415.05 to 14994.29) | 13.86(9.31 to 19.31) |  | 154050.01(145802.01 to 162471.11) | 79632.02(75252.3 to 84138.15) |  |
| Western Sub-Saharan Africa | Both | | 42428.5(32610.85 to 52655.2) | | 154197.62(103983.76 to 213921.72) |  | 877.16(564.82 to 1295.09) | 4131.47(2421.68 to 6397.18) |  | 41551.34(32001.35 to 51623.98) | 150066.15(101388.48 to 208604.28) |  |
|  | Male | | 809.58(448.15 to 1348.47) | | 2097.76(1166.77 to 3964.58) |  | 15.84(8.43 to 28.44) | 51.75(27.41 to 109.63) |  | 793.75(438.98 to 1321.49) | 2046.01(1138 to 3864.33) |  |
|  | Female | | 41618.91(31853.47 to 51774.51) | | 152099.86(101973.6 to 211475.17) |  | 861.32(552.96 to 1271.31) | 4.99(2.93 to 7.73) |  | 40757.59(31244.67 to 50641.17) | 148020.14(99285.08 to 206144.96) |  |

DALYs: disability-adjusted life years; YLDs: years lived with disability; YLLs: years of life lost; SDI: sociodemographic index; UI: uncertainty interval.

**Table S3.** Age-standardized DALYs, YLDs, and YLLs rates for breast cancer among adolescents and young adults globally and geographic regions in 1990 and 2021, as well as time trends.

| Characteristics | | | Sex | Age-standardized DALYs rates  (per 100,000)  (95% CI) | | | | |  | EAPC  (95% CI) | |  | Age-standardized  YLDs rates  (per 100,000)  (95% CI) | |  | EAPC  (95% CI) |  | Age-standardized YLLs rates  (per 100,000)  (95% CI) | |  | EAPC  (95% CI) |  |
| --- | --- | --- | --- | --- | --- | --- | --- | --- | --- | --- | --- | --- | --- | --- | --- | --- | --- | --- | --- | --- | --- | --- |
|  |  |  |  |  |  |  |  |  |  |  |  |  |  |  |  |  |  |  |  |  |  |  |
|  |  |  |  | **1990** | | | **2021** | |  | **1990-2021** | |  | **1990** | **2021** |  | **1990-2021** |  | **1990** | **2021** |  | **1990-2021** |  |
| Global | | Both | | 78.22(72.33 to 85.5) | | | 80.8(72.84 to 89.23) | |  | 0.01(-0.11 to 0.13) | |  | 3.1(2.15 to 4.24) | 4.1(2.84 to 5.63) |  | 0.86(0.76 to 0.96) |  | 75.13(69.49 to 82.08) | 76.7(69.4 to 84.86) |  | -0.03(-0.15 to 0.09) |  |
|  |  | Male | | 1.4(1.05 to 1.73) | | | 1.97(1.24 to 2.76) | |  | 1.25(1.14 to 1.36) | |  | 0.05(0.03 to 0.07) | 0.1(0.06 to 0.16) |  | 2.91(2.74 to 3.09) |  | 1.36(1.01 to 1.67) | 1.87(1.18 to 2.62) |  | 1.18(1.07 to 1.29) |  |
|  |  | Female | | 157.15(145.16 to 171.89) | | | 161.32(145.54 to 178.4) | |  | -0.02(-0.15 to 0.1) | |  | 6.23(4.33 to 8.52) | 8.18(5.66 to 11.24) |  | 0.82(0.71 to 0.92) |  | 150.92(139.49 to 165.1) | 153.14(138.58 to 169.6) |  | -0.06(-0.19 to 0.06) |  |
| SDI |  | | | |  |  | |  | | |  | | | | | | | | | | |  |
| High | | Both | | 93.54(90.1 to 97.28) | | | 57.72(54.6 to 61.27) | |  | -1.66(-1.82 to -1.5) | |  | 6.41(4.49 to 8.75) | 6.16(4.32 to 8.43) |  | -0.17(-0.27 to -0.07) |  | 87.13(84.52 to 89.83) | 51.56(49.23 to 53.99) |  | -1.81(-1.98 to -1.64) |  |
|  |  | Male | | 0.58(0.54 to 0.63) | | | 0.63(0.56 to 0.76) | |  | 0.06(-0.15 to 0.27) | |  | 0.05(0.04 to 0.07) | 0.07(0.05 to 0.1) |  | 0.83 (0.59 to 1.08) |  | 0.53(0.49 to 0.58) | 0.56(0.49 to 0.67) |  | -0.03(-0.24 to 0.17) |  |
|  |  | Female | | 188.96(181.99 to 196.53) | | | 118.96(112.53 to 126.25) | |  | -1.59(-1.75 to -1.43) | |  | 12.94(9.07 to 17.66) | 12.69(8.9 to 17.38) |  | -0.09(-0.19 to 0) |  | 176.02(170.75 to 181.49) | 106.27(101.47 to 111.28) |  | -1.73(-1.9 to -1.56) |  |
| High-middle | | Both | | 82.69(74.83 to 92.57) | | | 60.99(53.54 to 69.86) | |  | -1.06(-1.17 to -0.94) | |  | 3.44(2.38 to 4.76) | 4.71(3.21 to 6.65) |  | 1.11(0.98 to 1.25) |  | 79.25(71.81 to 88.87) | 56.28(49.65 to 64.35) |  | -1.19(-1.3 to -1.08) |  |
|  |  | Male | | 1.31(0.95 to 1.67) | | | 1.47(0.77 to 1.94) | |  | 0.57(0.4 to 0.74) | |  | 0.06(0.04 to 0.08) | 0.16(0.07 to 0.26) |  | 3.89(3.56 to 4.22) |  | 1.25(0.91 to 1.59) | 1.31(0.69 to 1.72) |  | 0.32(0.16 to 0.49) |  |
|  |  | Female | | 166.87(150.85 to 187.09) | | | 124.17(108.73 to 142.36) | |  | -1.05(-1.18 to -0.92) | |  | 6.93(4.79 to 9.6) | 9.54(6.5 to 13.48) |  | 1.11(0.96 to 1.25) |  | 159.94(144.71 to 179.53) | 114.63(100.9 to 131.24) |  | -1.18(-1.3 to -1.06) |  |
| Middle | | Both | | 72.87(64.82 to 82.57) | | | 76.54(69.36 to 84.55) | |  | 0.08(0 to 0.16) | |  | 2.23(1.53 to 3.06) | 4.07(2.83 to 5.68) |  | 1.94(1.86 to 2.02) |  | 70.65(62.93 to 80.02) | 72.47(65.81 to 79.74) |  | 0(-0.08 to 0.08) |  |
|  |  | Male | | 1.42(0.85 to 1.8) | | | 1.95(0.93 to 2.52) | |  | 1.46(1.27 to 1.65) | |  | 0.04(0.03 to 0.07) | 0.13(0.05 to 0.2) |  | 4.29(3.98 to 4.61) |  | 1.37(0.82 to 1.73) | 1.82(0.87 to 2.35) |  | 1.33(1.13 to 1.52) |  |
|  |  | Female | | 147.26(130.7 to 167.05) | | | 152.59(138.09 to 168.77) | |  | 0.02(-0.07 to 0.11) | |  | 4.5(3.08 to 6.18) | 8.08(5.6 to 11.31) |  | 1.87(1.77 to 1.96) |  | 142.76(126.93 to 161.99) | 144.51(130.97 to 159.22) |  | -0.06(-0.15 to 0.03) |  |
| Low-middle | | Both | | 68.1(57.77 to 80.17) | | | 100.93(85.4 to 119.59) | |  | 1.19(1.12 to 1.26) | |  | 1.57(1.05 to 2.17) | 3.27(2.22 to 4.53) |  | 2.33(2.27 to 2.39) |  | 66.53(56.55 to 78.29) | 97.66(82.68 to 115.5) |  | 1.16(1.09 to 1.23) |  |
|  |  | Male | | 1.25(0.96 to 1.72) | | | 1.94(1.27 to 3.02) | |  | 1.49(1.23 to 1.74) | |  | 0.03(0.02 to 0.04) | 0.05(0.03 to 0.09) |  | 2.45(2.3 to 2.61) |  | 1.23(0.94 to 1.69) | 1.89(1.23 to 2.93) |  | 1.46(1.2 to 1.72) |  |
|  |  | Female | | 136.35(115.51 to 160.76) | | | 200.18(169.1 to 237.34) | |  | 1.14(1.07 to 1.21) | |  | 3.14(2.11 to 4.35) | 6.49(4.41 to 9) |  | 2.28(2.21 to 2.35) |  | 133.21(113.11 to 157.09) | 193.69(163.77 to 229.28) |  | 1.11(1.04 to 1.18) |  |
| Low | | Both | | 71.52(58.32 to 87.09) | | | 98.83(80.03 to 120.16) | |  | 0.97(0.85 to 1.09) | |  | 1.4(0.93 to 1.97) | 2.55(1.73 to 3.67) |  | 1.89 (1.74 to 2.04) |  | 70.12(57.16 to 85.23) | 96.28(77.85 to 116.96) |  | 0.95(0.83 to 1.07) |  |
|  |  | Male | | 4.02(2.9 to 6.11) | | | 4.39(3.01 to 7.5) | |  | 0.12(0.06 to 0.18) | |  | 0.07(0.05 to 0.12) | 0.1(0.06 to 0.19) |  | 0.96(0.9 to 1.01) |  | 3.95(2.85 to 6) | 4.29(2.93 to 7.32) |  | 0.11(0.04 to 0.17) |  |
|  |  | Female | | 136.74(110.9 to 167.47) | | | 189.1(151.89 to 230.03) | |  | 0.97(0.86 to 1.08) | |  | 2.68(1.77 to 3.78) | 4.89(3.31 to 7.04) |  | 1.89(1.75 to 2.03) |  | 134.05(108.71 to 163.92) | 184.21(148 to 224.07) |  | 0.95(0.84 to 1.06) |  |
| Region |  | | | |  |  | |  | | |  | | | | | | | | | | |  |
| Andean Latin America | | Both | | 67.83(53.57 to 87.09) | | | 59.91(44.67 to 79.9) | |  | -0.51(-0.66 to -0.36) | |  | 1.71(1.06 to 2.59) | 2.77(1.7 to 4.3) |  | 1.54(1.36 to 1.72) |  | 66.12(52.14 to 84.83) | 57.14(42.75 to 76.16) |  | -0.58(-0.73 to -0.44) |  |
|  |  | Male | | 0.84(0.51 to 1.34) | | | 1.01(0.6 to 1.55) | |  | 1.34(0.67 to 2.01) | |  | 0.02(0.01 to 0.03) | 0.04(0.02 to 0.07) |  | 3.38 (2.76 to 4) |  | 0.82(0.5 to 1.32) | 0.97(0.58 to 1.49) |  | 1.28(0.61 to 1.95) |  |
|  |  | Female | | 131.85(104.04 to 169.36) | | | 117.57(87.38 to 157.16) | |  | -0.49(-0.64 to -0.34) | |  | 3.33(2.05 to 5.04) | 5.44(3.35 to 8.46) |  | 1.56(1.38 to 1.75) |  | 128.52(101.18 to 165.04) | 112.13(83.55 to 149.87) |  | -0.56(-0.71 to -0.41) |  |
| Australasia | | Both | | 100.08(86.17 to 115.23) | | | 54.87(46.08 to 65.02) | |  | -2.12(-2.25 to -1.99) | |  | 6.54(4.15 to 9.49) | 6.33(3.92 to 9.38) |  | -0.1(-0.27 to 0.06) |  | 93.53(80.42 to 106.95) | 48.54(40.85 to 57.42) |  | -2.32(-2.45 to -2.18) |  |
|  |  | Male | | 0.5(0.39 to 0.63) | | | 0.49(0.37 to 0.65) | |  | -0.14(-0.45 to 0.17) | |  | 0.06(0.03 to 0.1) | 0.07(0.04 to 0.12) |  | 0.76(0.34 to 1.19) |  | 0.44(0.35 to 0.56) | 0.42(0.31 to 0.54) |  | -0.28(-0.58 to 0.02) |  |
|  |  | Female | | 198.57(170.93 to 228.72) | | | 107.92(90.56 to 128.05) | |  | -2.13(-2.26 to -1.99) | |  | 12.96(8.22 to 18.81) | 12.44(7.7 to 18.45) |  | -0.11(-0.26 to 0.04) |  | 185.6(159.53 to 212.28) | 95.48(80.27 to 113) |  | -2.33(-2.47 to -2.19) |  |
| Caribbean | | Both | | 98.74(83 to 121.01) | | | 98.17(70.76 to 133.13) | |  | 0.11(-0.05 to 0.26) | |  | 3.49(2.32 to 4.81) | 3.81(2.36 to 5.52) |  | 0.28(0.16 to 0.4) |  | 95.25(79.86 to 116.75) | 94.35(67.73 to 128.21) |  | 0.1(-0.06 to 0.26) |  |
|  |  | Male | | 1.13(0.87 to 1.5) | | | 1.83(1.34 to 2.51) | |  | 1.47(1.08 to 1.87) | |  | 0.03(0.02 to 0.04) | 0.06(0.04 to 0.09) |  | 2.25(1.73 to 2.77) |  | 1.1(0.85 to 1.47) | 1.77(1.29 to 2.44) |  | 1.45(1.06 to 1.84) |  |
|  |  | Female | | 190.53(159.94 to 233.68) | | | 192.49(138.15 to 261.79) | |  | 0.16(0.01 to 0.31) | |  | 6.75(4.49 to 9.31) | 7.49(4.64 to 10.85) |  | 0.33(0.21 to 0.45) |  | 183.78(153.89 to 225.54) | 185(132.24 to 252.04) |  | 0.15(0 to 0.31) |  |
| Central Asia | | Both | | 115.71(107.12 to 123.98) | | | 69.22(58.54 to 82.01) | |  | -1.63(-1.71 to -1.55) | |  | 3.85(2.61 to 5.41) | 2.99(1.99 to 4.31) |  | -0.69(-0.82 to -0.56) |  | 111.86(103.64 to 119.93) | 66.23(55.95 to 78.69) |  | -1.67(-1.75 to -1.58) |  |
|  |  | Male | | 0.32(0.26 to 0.39) | | | 0.54(0.44 to 0.66) | |  | 2.42(1.73 to 3.11) | |  | 0.01(0.01 to 0.02) | 0.02(0.02 to 0.04) |  | 3.22(2.5 to 3.94) |  | 0.31(0.25 to 0.38) | 0.52(0.42 to 0.63) |  | 2.39(1.7 to 3.07) |  |
|  |  | Female | | 227.99(211.08 to 244.32) | | | 138.17(116.75 to 163.83) | |  | -1.57(-1.65 to -1.49) | |  | 7.58(5.15 to 10.65) | 5.97(3.96 to 8.61) |  | -0.63(-0.77 to -0.49) |  | 220.41(204.22 to 236.34) | 132.2(111.58 to 157.19) |  | -1.61(-1.69 to -1.53) |  |
| Central Europe | | Both | | 91.34(85.24 to 97.89) | | | 60.04(53.43 to 66.89) | |  | -1.36(-1.55 to -1.16) | |  | 3.92(2.71 to 5.38) | 4.57(3.11 to 6.35) |  | 0.58(0.43 to 0.74) |  | 87.41(81.53 to 93.71) | 55.47(49.63 to 61.68) |  | -1.48(-1.68 to -1.28) |  |
|  |  | Male | | 0.77(0.69 to 0.88) | | | 0.8(0.7 to 0.91) | |  | -0.09(-0.3 to 0.13) | |  | 0.04(0.03 to 0.05) | 0.06(0.04 to 0.09) |  | 1.43(1.15 to 1.72) |  | 0.73(0.65 to 0.84) | 0.74(0.65 to 0.83) |  | -0.19(-0.4 to 0.02) |  |
|  |  | Female | | 183.86(171.57 to 197.12) | | | 122.19(108.66 to 136.18) | |  | -1.31(-1.52 to -1.11) | |  | 7.9(5.45 to 10.83) | 9.29(6.32 to 12.91) |  | 0.63(0.47 to 0.79) |  | 175.96(164.1 to 188.67) | 112.9(100.92 to 125.58) |  | -1.44(-1.64 to -1.23) |  |
| Central Latin America | | Both | | 72.34(68.5 to 76.51) | | | 77.22(64.95 to 89.17) | |  | 0.16(-0.05 to 0.36) | |  | 2.97(2.11 to 4.05) | 5.25(3.61 to 7.48) |  | 1.72(1.57 to 1.87) |  | 69.37(65.64 to 73.32) | 71.97(60.44 to 83.03) |  | 0.07(-0.14 to 0.27) |  |
|  |  | Male | | 0.58(0.53 to 0.64) | | | 0.81(0.72 to 0.91) | |  | 0.51(-0.13 to 1.15) | |  | 0.01(0.01 to 0.02) | 0.03(0.02 to 0.04) |  | 2.02(1.37 to 2.66) |  | 0.57(0.52 to 0.63) | 0.78(0.69 to 0.87) |  | 0.46(-0.17 to 1.11) |  |
|  |  | Female | | 139.77(132.32 to 147.85) | | | 148.44(124.76 to 171.49) | |  | 0.16(-0.06 to 0.38) | |  | 5.75(4.09 to 7.83) | 10.12(6.96 to 14.41) |  | 1.73(1.57 to 1.88) |  | 134.02(126.8 to 141.69) | 138.31(116.09 to 159.64) |  | 0.07(-0.15 to 0.29) |  |
| Central Sub-Saharan Africa | | Both | | 66.97(44.32 to 98.84) | | | 88.13(60.39 to 126.19) | |  | 0.91(0.69 to 1.12) | |  | 1.28(0.69 to 2.12) | 2.16(1.2 to 3.47) |  | 1.73(1.44 to 2.01) |  | 65.7(43.43 to 97.08) | 85.97(59.06 to 123.19) |  | 0.89(0.68 to 1.1) |  |
|  |  | Male | | 2.43(1.38 to 4.45) | | | 2.49(1.38 to 4.58) | |  | 0.04(-0.15 to 0.24) | |  | 0.04(0.02 to 0.09) | 0.06(0.03 to 0.12) |  | 0.81(0.52 to 1.1) |  | 2.39(1.35 to 4.36) | 2.43(1.34 to 4.47) |  | 0.03(-0.17 to 0.22) |  |
|  |  | Female | | 129.7(84.94 to 192.71) | | | 172.61(117.68 to 248.65) | |  | 0.94(0.75 to 1.13) | |  | 2.47(1.32 to 4.13) | 4.23(2.35 to 6.84) |  | 1.77(1.51 to 2.02) |  | 127.23(83.3 to 189.28) | 168.39(114.94 to 242.33) |  | 0.93(0.74 to 1.11) |  |
| East Asia | | Both | | 65.9(51.91 to 82.69) | | | 52.61(39.34 to 68.49) | |  | -0.97(-1.12 to -0.82) | |  | 2.24(1.44 to 3.21) | 4.3(2.69 to 6.47) |  | 2.18(2.05 to 2.32) |  | 63.66(50.09 to 79.81) | 48.31(36.19 to 62.86) |  | -1.15(-1.29 to -1) |  |
|  |  | Male | | 1.56(0.86 to 2.17) | | | 2.52(0.93 to 3.49) | |  | 2.47(2.09 to 2.85) | |  | 0.07(0.04 to 0.1) | 0.29(0.1 to 0.46) |  | 6.17(5.62 to 6.72) |  | 1.49(0.82 to 2.07) | 2.23(0.81 to 3.09) |  | 2.19(1.82 to 2.56) |  |
|  |  | Female | | 134.82(105.87 to 169.75) | | | 106.21(78.93 to 138.92) | |  | -1.07(-1.24 to -0.9) | |  | 4.56(2.95 to 6.55) | 8.6(5.32 to 13) |  | 2.04(1.89 to 2.19) |  | 130.26(102.15 to 163.78) | 97.61(72.6 to 127.59) |  | -1.25(-1.41 to -1.08) |  |
| Eastern Europe | | Both | | 98.78(92.5 to 105.4) | | | 67.15(57.91 to 78.47) | |  | -1.6(-1.75 to -1.44) | |  | 3.91(2.69 to 5.44) | 3.95(2.79 to 5.49) |  | -0.15(-0.35 to 0.04) |  | 94.87(88.94 to 100.99) | 63.2(54.46 to 73.92) |  | -1.67(-1.83 to -1.51) |  |
|  |  | Male | | 1.46(1.36 to 1.55) | | | 0.68(0.61 to 0.74) | |  | -2.72(-3.32 to -2.12) | |  | 0.06(0.05 to 0.09) | 0.05(0.03 to 0.06) |  | -1.21(-1.74 to -0.67) |  | 1.4(1.31 to 1.48) | 0.63(0.57 to 0.69) |  | -2.81(-3.41 to -2.2) |  |
|  |  | Female | | 195.4(182.89 to 208.62) | | | 133.95(115.42 to 156.66) | |  | -1.58(-1.73 to -1.44) | |  | 7.73(5.32 to 10.77) | 7.88(5.56 to 10.95) |  | -0.14(-0.35 to 0.06) |  | 187.66(175.84 to 199.86) | 126.07(108.53 to 147.57) |  | -1.66(-1.81 to -1.5) |  |
| Eastern Sub-Saharan Africa | | Both | | 97.12(74.39 to 124.1) | | | 127.35(97.11 to 166.5) | |  | 0.7(0.54 to 0.86) | |  | 1.87(1.2 to 2.72) | 3.24(2.1 to 4.86) |  | 1.61(1.42 to 1.8) |  | 95.25(72.92 to 121.61) | 124.11(94.77 to 162.36) |  | 0.68(0.52 to 0.84) |  |
|  |  | Male | | 9.19(6.43 to 14.57) | | | 10.15(6.07 to 18.92) | |  | 0.2(0.14 to 0.26) | |  | 0.17(0.1 to 0.28) | 0.24(0.12 to 0.47) |  | 1.04(1.01 to 1.08) |  | 9.02(6.31 to 14.3) | 9.91(5.92 to 18.49) |  | 0.18(0.12 to 0.25) |  |
|  |  | Female | | 178.2(135.05 to 230.4) | | | 236.73(179.09 to 311.55) | |  | 0.75(0.59 to 0.9) | |  | 3.44(2.2 to 5.05) | 6.04(3.89 to 9.15) |  | 1.65(1.48 to 1.83) |  | 174.76(132.46 to 226.1) | 230.69(174.53 to 304.37) |  | 0.73(0.57 to 0.88) |  |
| High-income Asia Pacific | | Both | | 56.07(51.58 to 60.94) | | | 45.52(40.88 to 51.15) | |  | -0.78(-0.9 to -0.66) | |  | 4.13(2.78 to 5.75) | 5.77(3.78 to 8.23) |  | 1.18(0.94 to 1.41) |  | 51.94(47.89 to 56.2) | 39.76(36.09 to 44.42) |  | -1(-1.11 to -0.88) |  |
|  |  | Male | | 0.22(0.16 to 0.3) | | | 0.13(0.11 to 0.16) | |  | -2.38(-2.69 to -2.07) | |  | 0.01(0.01 to 0.02) | 0.01(0.01 to 0.02) |  | 0.05(-0.37 to 0.46) |  | 0.21(0.15 to 0.28) | 0.11(0.1 to 0.14) |  | -2.59(-2.89 to -2.28) |  |
|  |  | Female | | 113.44(104.33 to 123.28) | | | 93.91(84.33 to 105.51) | |  | -0.72(-0.83 to -0.61) | |  | 8.35(5.62 to 11.63) | 11.9(7.79 to 16.98) |  | 1.24(1.01 to 1.46) |  | 105.08(96.87 to 113.73) | 82.01(74.43 to 91.64) |  | -0.93(-1.04 to -0.83) |  |
| High-income North America | | Both | | 111.88(107.26 to 116.87) | | | 66.34(61.99 to 71.05) | |  | -1.76(-1.99 to -1.52) | |  | 8.86(6.22 to 12.04) | 7.24(5.1 to 9.91) |  | -0.72(-0.85 to -0.59) |  | 103.01(99.77 to 106.44) | 59.1(55.7 to 62.42) |  | -1.87(-2.11 to -1.62) |  |
|  |  | Male | | 0.72(0.68 to 0.77) | | | 0.74(0.68 to 0.8) | |  | -0.23(-0.56 to 0.09) | |  | 0.1(0.07 to 0.14) | 0.12(0.08 to 0.16) |  | 0.09(-0.24 to 0.41) |  | 0.62(0.59 to 0.65) | 0.62(0.58 to 0.65) |  | -0.29(-0.61 to 0.04) |  |
|  |  | Female | | 222.05(212.88 to 231.97) | | | 131.71(123.05 to 141.09) | |  | -1.76(-1.99 to -1.53) | |  | 17.55(12.31 to 23.85) | 14.34(10.1 to 19.64) |  | -0.73(-0.85 to -0.6) |  | 204.51(198.05 to 211.33) | 117.37(110.57 to 123.98) |  | -1.87(-2.11 to -1.63) |  |
| North Africa and Middle East | | Both | | 47.04(38.96 to 57.81) | | | 65.89(55.25 to 78.79) | |  | 1.36(1.24 to 1.47) | |  | 1.96(1.28 to 2.84) | 4.83(3.23 to 6.79) |  | 3.2(3.09 to 3.32) |  | 45.08(37.17 to 55.41) | 61.06(51.33 to 72.85) |  | 1.25(1.13 to 1.36) |  |
|  |  | Male | | 1.2(0.81 to 1.75) | | | 1.18(0.82 to 1.65) | |  | -0.06(-0.1 to -0.02) | |  | 0.03(0.02 to 0.05) | 0.05(0.03 to 0.08) |  | 1.91(1.82 to 2) |  | 1.17(0.79 to 1.71) | 1.13(0.79 to 1.57) |  | -0.13(-0.17 to -0.08) |  |
|  |  | Female | | 95.69(79.04 to 117.97) | | | 137.86(115.61 to 164.95) | |  | 1.48(1.35 to 1.62) | |  | 4.01(2.61 to 5.82) | 10.13(6.79 to 14.26) |  | 3.33(3.19 to 3.47) |  | 91.68(75.43 to 112.9) | 127.73(107.36 to 152.47) |  | 1.37(1.24 to 1.51) |  |
| Oceania | | Both | | 131.29(88.41 to 185.87) | | | 156.04(104.46 to 227.02) | |  | 0.51(0.37 to 0.65) | |  | 3.47(1.97 to 5.57) | 3.9(2.14 to 6.53) |  | 0.12(-0.11 to 0.34) |  | 127.82(85.95 to 180.63) | 152.14(101.7 to 221.47) |  | 0.52(0.38 to 0.66) |  |
|  |  | Male | | 0.8(0.4 to 1.49) | | | 1.09(0.49 to 2.05) | |  | 1.09(0.98 to 1.19) | |  | 0.02(0.01 to 0.04) | 0.03(0.01 to 0.05) |  | 1.43(1.33 to 1.52) |  | 0.78(0.39 to 1.45) | 1.07(0.48 to 2) |  | 1.08(0.97 to 1.19) |  |
|  |  | Female | | 268.52(180.63 to 380.41) | | | 311.39(208.16 to 453.91) | |  | 0.43(0.28 to 0.57) | |  | 7.1(4.04 to 11.4) | 7.78(4.25 to 13.04) |  | 0.04(-0.19 to 0.27) |  | 261.42(175.64 to 369.79) | 303.6(202.57 to 442.66) |  | 0.44(0.3 to 0.58) |  |
| South Asia | | Both | | 62.1(52.78 to 72.2) | | | 93.5(76.75 to 114.95) | |  | 1.3(1.2 to 1.39) | |  | 1.33(0.9 to 1.83) | 2.91(1.9 to 4.16) |  | 2.59(2.52 to 2.67) |  | 60.78(51.61 to 70.64) | 90.59(74.51 to 111.57) |  | 1.26(1.17 to 1.36) |  |
|  |  | Male | | 1.06(0.79 to 1.46) | | | 1.51(0.91 to 2.15) | |  | 1.2(0.82 to 1.58) | |  | 0.02(0.01 to 0.03) | 0.04(0.02 to 0.07) |  | 2.32(2.07 to 2.58) |  | 1.04(0.78 to 1.43) | 1.46(0.88 to 2.08) |  | 1.17(0.79 to 1.55) |  |
|  |  | Female | | 127.71(108.39 to 148.57) | | | 188.42(154.6 to 232.16) | |  | 1.2(1.11 to 1.29) | |  | 2.73(1.85 to 3.76) | 5.87(3.82 to 8.38) |  | 2.5(2.41 to 2.58) |  | 124.98(105.99 to 145.32) | 182.55(149.87 to 225.34) |  | 1.17(1.08 to 1.26) |  |
| Southeast Asia | | Both | | 87.69(68.87 to 112.53) | | | 102.46(85.02 to 122.99) | |  | 0.35(0.22 to 0.48) | |  | 2.26(1.41 to 3.29) | 3.95(2.65 to 5.58) |  | 1.67(1.54 to 1.8) |  | 85.43(67.14 to 109.62) | 98.51(81.93 to 118.57) |  | 0.31(0.18 to 0.44) |  |
|  |  | Male | | 1.16(0.7 to 1.49) | | | 1.6(0.71 to 2.25) | |  | 1.06(0.88 to 1.24) | |  | 0.03(0.02 to 0.04) | 0.05(0.02 to 0.08) |  | 2.14(2.03 to 2.24) |  | 1.13(0.68 to 1.45) | 1.55(0.69 to 2.17) |  | 1.03(0.84 to 1.21) |  |
|  |  | Female | | 172.18(134.97 to 221.24) | | | 205.61(170.31 to 247.34) | |  | 0.43(0.31 to 0.55) | |  | 4.43(2.76 to 6.46) | 7.93(5.32 to 11.21) |  | 1.76(1.63 to 1.88) |  | 167.75(131.59 to 215.49) | 197.68(164.1 to 238.11) |  | 0.39(0.27 to 0.51) |  |
| Southern Latin America | | Both | | 109.65(94.36 to 127.79) | | | 80.89(68.55 to 94.69) | |  | -0.79(-1.01 to -0.57) | |  | 3.76(2.35 to 5.63) | 4.55(2.81 to 6.81) |  | 0.76(0.59 to 0.92) |  | 105.89(90.9 to 123.41) | 76.34(65.1 to 88.95) |  | -0.86(-1.09 to -0.64) |  |
|  |  | Male | | 0.87(0.64 to 1.16) | | | 0.82(0.62 to 1.05) | |  | -0.3(-0.69 to 0.09) | |  | 0.03(0.02 to 0.04) | 0.04(0.02 to 0.07) |  | 1.31(0.93 to 1.7) |  | 0.85(0.62 to 1.13) | 0.78(0.59 to 1) |  | -0.37(-0.76 to 0.02) |  |
|  |  | Female | | 215(184.9 to 250.78) | | | 159.34(134.87 to 186.7) | |  | -0.78(-1 to -0.55) | |  | 7.37(4.6 to 11.05) | 8.97(5.54 to 13.43) |  | 0.77(0.6 to 0.94) |  | 207.63(178.08 to 242.14) | 150.37(128.12 to 175.36) |  | -0.85(-1.08 to -0.62) |  |
| Southern Sub-Saharan Africa | | Both | | 121.95(102.34 to 144.58) | | | 114.79(93.07 to 142.95) | |  | 0.16(-0.62 to 0.94) | |  | 3.12(2.1 to 4.46) | 3.49(2.34 to 4.96) |  | 0.64(-0.1 to 1.38) |  | 118.83(99.59 to 140.65) | 111.31(90.2 to 138.41) |  | 0.15(-0.63 to 0.93) |  |
|  |  | Male | | 3.82(2.69 to 4.84) | | | 3.85(2.67 to 5.7) | |  | -0.2(-0.83 to 0.42) | |  | 0.09(0.06 to 0.13) | 0.11(0.07 to 0.18) |  | 0.55(0.03 to 1.06) |  | 3.73(2.63 to 4.73) | 3.73(2.58 to 5.54) |  | -0.22(-0.85 to 0.41) |  |
|  |  | Female | | 233.16(194.92 to 277.18) | | | 224.15(180.8 to 279.83) | |  | 0.32(-0.46 to 1.11) | |  | 5.97(4.02 to 8.55) | 6.81(4.55 to 9.7) |  | 0.8(0.05 to 1.55) |  | 227.19(190.01 to 269.5) | 217.34(175.25 to 271.16) |  | 0.31(-0.47 to 1.1) |  |
| Tropical Latin America | | Both | | 83.61(77.27 to 90.4) | | | 99.8(92.01 to 108.32) | |  | 0.38(0.27 to 0.5) | |  | 2.46(1.68 to 3.36) | 4.42(3.08 to 5.99) |  | 1.69(1.57 to 1.82) |  | 81.15(75.09 to 87.69) | 95.38(87.88 to 103.29) |  | 0.33(0.22 to 0.45) |  |
|  |  | Male | | 0.67(0.6 to 0.74) | | | 1.24(1.12 to 1.36) | |  | 2.17(1.69 to 2.66) | |  | 0.02(0.01 to 0.02) | 0.04(0.03 to 0.06) |  | 3.36(2.92 to 3.81) |  | 0.65(0.59 to 0.72) | 1.19(1.08 to 1.31) |  | 2.14(1.65 to 2.63) |  |
|  |  | Female | | 162.99(150.57 to 176.27) | | | 194.79(179.48 to 211.53) | |  | 0.4(0.29 to 0.51) | |  | 4.79(3.28 to 6.55) | 8.64(6.03 to 11.71) |  | 1.71(1.59 to 1.83) |  | 158.2(146.34 to 171.01) | 186.15(171.42 to 201.68) |  | 0.35(0.24 to 0.46) |  |
| Western Europe | | Both | | 111.3(105.16 to 117.71) | | | 60.67(56.42 to 65.22) | |  | -1.99(-2.14 to -1.85) | |  | 7.28(5 to 10.11) | 6.92(4.65 to 9.64) |  | -0.13(-0.26 to 0.01) |  | 104.02(98.45 to 109.68) | 53.75(50.78 to 56.8) |  | -2.18(-2.34 to -2.02) |  |
|  |  | Male | | 0.64(0.58 to 0.71) | | | 0.59(0.53 to 0.67) | |  | -0.79(-1.4 to -0.19) | |  | 0.03(0.02 to 0.05) | 0.06(0.04 to 0.08) |  | 1.17(0.51 to 1.84) |  | 0.61(0.55 to 0.67) | 0.54(0.48 to 0.6) |  | -0.95(-1.55 to -0.35) |  |
|  |  | Female | | 223.79(211.42 to 236.71) | | | 121.36(112.81 to 130.51) | |  | -2.02(-2.16 to -1.87) | |  | 14.65(10.06 to 20.34) | 13.86(9.31 to 19.31) |  | -0.15(-0.28 to -0.02) |  | 209.13(197.93 to 220.57) | 107.49(101.52 to 113.64) |  | -2.2(-2.36 to -2.05) |  |
| Western Sub-Saharan Africa | | Both | | 73.27(56.47 to 90.75) | | | 99.86(67.44 to 138.33) | |  | 0.94(0.82 to 1.05) | |  | 1.51(0.98 to 2.23) | 2.67(1.57 to 4.13) |  | 1.8(1.67 to 1.93) |  | 71.76(55.42 to 88.99) | 97.19(65.76 to 134.91) |  | 0.92(0.8 to 1.03) |  |
|  |  | Male | | 2.83(1.58 to 4.68) | | | 2.9(1.64 to 5.41) | |  | -0.1(-0.23 to 0.04) | |  | 0.06(0.03 to 0.1) | 0.07(0.04 to 0.15) |  | 0.7(0.55 to 0.85) |  | 2.77(1.55 to 4.59) | 2.83(1.6 to 5.27) |  | -0.12(-0.25 to 0.02) |  |
|  |  | Female | | 143.91(110.49 to 178.63) | | | 186.6(125.26 to 259.12) | |  | 0.8(0.68 to 0.92) | |  | 2.98(1.91 to 4.38) | 4.99(2.93 to 7.73) |  | 1.66(1.53 to 1.79) |  | 140.93(108.39 to 174.71) | 181.6(121.95 to 252.62) |  | 0.78(0.66 to 0.89) |  |

DALYs: disability-adjusted life years; YLDs: years lived with disability; YLLs: years of life lost; SDI: sociodemographic index; EAPC: estimated annual percentage change; CI: confidence interval.

**Table S4.** The number and age-standardized rate of incidence caused by breast cancer at the national level among adolescents and young adults

in 1990 and 2021, as well as time trends.

| **Characteristics** | **Sex** | **1990** | |  | **2021** | |  | **1990-2021** |
| --- | --- | --- | --- | --- | --- | --- | --- | --- |
|  |  | **Number  (95% UI)** | **ASIR (per 100000）  (95% CI)** |  | **Number  (95% UI)** | **ASIR (per 100000）  (95% CI)** |  | **EAPC (95% CI)** |
| **Afghanistan** | Both | 52.81(18.69 to 113.07 | 2.64(0.93 to 5.69) |  | 358.34(138.4 to 740.75) | 4.21(1.62 to 8.7) |  | 1.54(1.22 to 1.86) |
| **Albania** | Both | 33.21(23.82 to 44.67) | 2.62(1.89 to 3.51) |  | 42.47(27.7 to 61.07) | 4.43(2.9 to 6.36) |  | 2.2(1.66 to 2.74) |
| **Algeria** | Both | 190.14(120.04 to 285.85) | 2.47(1.57 to 3.71) |  | 836.26(511.32 to 1310.71) | 4.28(2.62 to 6.72) |  | 1.53(1.39 to 1.68) |
| **American Samoa** | Both | 1.17(0.73 to 1.8) | 6.7(4.23 to 10.25) |  | 1.89(1.2 to 2.85) | 11.54(7.32 to 17.4) |  | 1.75(1.61 to 1.89) |
| **Andorra** | Both | 2.44(1.44 to 3.82) | 8.79(5.21 to 13.76) |  | 3.16(1.74 to 5.06) | 9.98(5.46 to 16.02) |  | 0.81(0.47 to 1.15) |
| **Angola** | Both | 61.44(35.91 to 99) | 1.88(1.11 to 3.03) |  | 373.62(213.22 to 603.42) | 3.76(2.16 to 6.05) |  | 2.58(2.35 to 2.81) |
| **Antigua and Barbuda** | Both | 1.94(1.55 to 2.38) | 8.15(6.53 to 10) |  | 2.94(2.34 to 3.61) | 8.02(6.37 to 9.83) |  | 0.75(0.45 to 1.04) |
| **Argentina** | Both | 691.93(551.39 to 859.24) | 5.83(4.65 to 7.24) |  | 1159.71(900.22 to 1470.84) | 6.39(4.96 to 8.1) |  | 0.37(0.16 to 0.58) |
| **Armenia** | Both | 137.56(120.6 to 156.26) | 9.59(8.41 to 10.89) |  | 56.83(44.89 to 69.8) | 4.28(3.38 to 5.25) |  | -2.49(-2.89 to -2.08) |
| **Australia** | Both | 597.93(485.77 to 725.09) | 8.39(6.82 to 10.18) |  | 837.46(643 to 1078.91) | 8.37(6.41 to 10.79) |  | -0.05(-0.23 to 0.12) |
| **Austria** | Both | 258.09(200.01 to 325.43) | 8.66(6.71 to 10.91) |  | 261.79(201.05 to 336.14) | 8.03(6.16 to 10.32) |  | 0.09(-0.13 to 0.31) |
| **Azerbaijan** | Both | 152.82(118.29 to 189.13) | 5.5(4.27 to 6.79) |  | 210.5(146.03 to 288.88) | 4.27(2.97 to 5.85) |  | -0.86(-1.12 to -0.6) |
| **Bahamas** | Both | 12.74(10.2 to 15.68) | 12.21(9.79 to 15.01) |  | 27.08(19.24 to 36.98) | 17.33(12.31 to 23.66) |  | 0.64(0.42 to 0.85) |
| **Bahrain** | Both | 12.74(8.21 to 18.54) | 4.54(2.93 to 6.6) |  | 67.77(43.5 to 99.98) | 8.35(5.35 to 12.35) |  | 1.59(1.1 to 2.08) |
| **Bangladesh** | Both | 575.01(314.53 to 971.06) | 1.59(0.88 to 2.67) |  | 2536.08(1497.77 to 4028.88) | 3.75(2.22 to 5.96) |  | 2.76(2.57 to 2.95) |
| **Barbados** | Both | 10.37(8.49 to 12.71) | 9.51(7.79 to 11.65) |  | 14.5(10.3 to 19.74) | 13.45(9.55 to 18.32) |  | 0.8(0.57 to 1.03) |
| **Belarus** | Both | 249.19(193.42 to 316.34) | 5.81(4.51 to 7.38) |  | 233.57(154.62 to 332.78) | 5.93(3.92 to 8.45) |  | -0.59(-0.85 to -0.34) |
| **Belgium** | Both | 448.1(354.35 to 559.32) | 11.15(8.81 to 13.92) |  | 350.91(266 to 449.52) | 8.87(6.72 to 11.37) |  | -0.9(-1.15 to -0.65) |
| **Belize** | Both | 1.16(0.93 to 1.43) | 2.03(1.63 to 2.52) |  | 7.02(5.5 to 9) | 4.12(3.22 to 5.28) |  | 2.58(2.16 to 3) |
| **Benin** | Both | 22.18(13.34 to 34.93) | 1.58(0.96 to 2.49) |  | 103.94(56.45 to 171.62) | 2.47(1.35 to 4.04) |  | 1.18(1.04 to 1.33) |
| **Bermuda** | Both | 2.8(2.13 to 3.63) | 9.58(7.28 to 12.44) |  | 2.48(1.76 to 3.37) | 11.52(8.12 to 15.69) |  | 0.31(0.13 to 0.48) |
| **Bhutan** | Both | 2.45(1.35 to 4.04) | 1.22(0.68 to 2.01) |  | 8.43(4.39 to 14.61) | 2.4(1.25 to 4.15) |  | 1.99(1.88 to 2.11) |
| **Bolivia (Plurinational State of)** | Both | 54.59(28.89 to 92.36) | 2.59(1.38 to 4.38) |  | 179.49(103.85 to 285.52) | 3.79(2.2 to 6.02) |  | 0.97(0.86 to 1.08) |
| **Bosnia and Herzegovina** | Both | 66.69(48.32 to 87.98) | 3.45(2.5 to 4.55) |  | 51.5(32.8 to 73.17) | 4.39(2.79 to 6.24) |  | 1.11(0.75 to 1.47) |
| **Botswana** | Both | 11.72(5.51 to 21.8) | 2.95(1.39 to 5.48) |  | 45.41(21.98 to 81.04) | 4.02(1.94 to 7.18) |  | 1.59(1.02 to 2.16) |
| **Brazil** | Both | 2165.42(1989.1 to 2366.82) | 3.88(3.57 to 4.24) |  | 6557.21(5942.92 to 7236.33) | 7.01(6.35 to 7.73) |  | 1.66(1.53 to 1.8) |
| **Brunei Darussalam** | Both | 5.66(3.42 to 8.55) | 4.73(2.85 to 7.14) |  | 14.96(9.77 to 21.77) | 6.46(4.21 to 9.4) |  | 1.71(1.51 to 1.92) |
| **Bulgaria** | Both | 270.49(211.11 to 335.91) | 8.35(6.51 to 10.38) |  | 206.39(151.49 to 273.02) | 8.45(6.18 to 11.2) |  | 0.38(0.1 to 0.65) |
| **Burkina Faso** | Both | 101.08(59 to 159.14) | 3.94(2.31 to 6.16) |  | 366.1(198.25 to 596.87) | 4.97(2.7 to 8.07) |  | 0.82(0.66 to 0.99) |
| **Burundi** | Both | 44.76(26.17 to 73.3) | 2.57(1.5 to 4.2) |  | 135.74(82.25 to 219.08) | 2.94(1.78 to 4.73) |  | 0.16(-0.03 to 0.35) |
| **Cabo Verde** | Both | 4.29(2.68 to 6.57) | 4.66(2.93 to 7.07) |  | 9.06(5.37 to 14.49) | 3.47(2.06 to 5.55) |  | -0.48(-0.87 to -0.09) |
| **Cambodia** | Both | 75.47(37.47 to 139.18) | 2.43(1.22 to 4.47) |  | 341.96(203.01 to 543.8) | 4.71(2.8 to 7.48) |  | 2.26(2.18 to 2.34) |
| **Cameroon** | Both | 75.28(45.86 to 118.07) | 2.49(1.52 to 3.89) |  | 421.06(231.91 to 685.65) | 3.84(2.13 to 6.22) |  | 1.24(1.09 to 1.38) |
| **Canada** | Both | 1234.58(978.46 to 1529.07) | 9.89(7.84 to 12.25) |  | 1280.18(959.99 to 1655.59) | 9.45(7.08 to 12.23) |  | -0.07(-0.22 to 0.09) |
| **Central African Republic** | Both | 15.79(9.19 to 25.51) | 1.91(1.11 to 3.08) |  | 47.45(26.19 to 77.01) | 2.64(1.46 to 4.29) |  | 0.97(0.79 to 1.15) |
| **Chad** | Both | 23.21(12.64 to 38.29) | 1.38(0.75 to 2.27) |  | 95.66(54.12 to 157.93) | 2.04(1.17 to 3.36) |  | 1.49(1.37 to 1.62) |
| **Chile** | Both | 174.14(136 to 218.99) | 3.34(2.61 to 4.19) |  | 371.29(282.22 to 478.27) | 4.88(3.71 to 6.28) |  | 1.63(1.37 to 1.89) |
| **China** | Both | 15891.03(12402.62 to 20002.45) | 3.17(2.47 to 3.99) |  | 34216.69(25300.61 to 44983.87) | 6.01(4.46 to 7.9) |  | 2.12(1.97 to 2.27) |
| **Colombia** | Both | 576.99(441.41 to 727.57) | 4.74(3.63 to 5.97) |  | 2078.12(1470.89 to 2858.05) | 10.34(7.32 to 14.22) |  | 2.73(2.43 to 3.03) |
| **Comoros** | Both | 4.18(2.15 to 6.63) | 3.21(1.68 to 5.05) |  | 15.69(9.86 to 25.17) | 5.51(3.47 to 8.82) |  | 1.23(0.75 to 1.71) |
| **Congo** | Both | 21.97(10.54 to 39.79) | 3.07(1.47 to 5.58) |  | 121.24(60.87 to 216.3) | 5.89(2.96 to 10.51) |  | 2.12(1.89 to 2.35) |
| **Cook Islands** | Both | 0.79(0.46 to 1.23) | 11.76(6.88 to 18.24) |  | 0.93(0.57 to 1.44) | 16.6(10.2 to 25.58) |  | 1.23(0.98 to 1.47) |
| **Costa Rica** | Both | 65.89(50.68 to 84.72) | 5.81(4.47 to 7.45) |  | 255.14(186.18 to 340.69) | 12.5(9.12 to 16.69) |  | 2.9(2.48 to 3.31) |
| **Coted'Ivoire** | Both | 78.56(47.04 to 121.39) | 2.09(1.26 to 3.21) |  | 393.32(224.15 to 632.02) | 3.89(2.22 to 6.24) |  | 2.19(2.04 to 2.33) |
| **Croatia** | Both | 147.46(114.73 to 187.35) | 7.2(5.6 to 9.16) |  | 109.2(80.61 to 143.03) | 7.36(5.41 to 9.67) |  | 0.45(0.09 to 0.82) |
| **Cuba** | Both | 267.58(205.31 to 338.05) | 6.44(4.94 to 8.12) |  | 220.01(157.71 to 298.24) | 5.57(3.99 to 7.55) |  | -0.44(-0.64 to -0.24) |
| **Cyprus** | Both | 17.65(11.75 to 26.03) | 5.68(3.79 to 8.37) |  | 49.35(32.63 to 70.77) | 7.49(4.92 to 10.83) |  | 1.17(0.57 to 1.77) |
| **Czechia** | Both | 231.97(181.96 to 289.13) | 5.62(4.41 to 7.01) |  | 292.94(215.85 to 385.7) | 7.94(5.84 to 10.46) |  | 1.05(0.75 to 1.35) |
| **Democratic People's Republic of Korea** | Both | 228.69(127.9 to 375.39) | 3.04(1.7 to 4.98) |  | 487.85(280.56 to 794.18) | 4.45(2.56 to 7.25) |  | 1.37(1.23 to 1.51) |
| **Democratic Republic of the Congo** | Both | 217.05(124.55 to 357.11) | 1.91(1.1 to 3.13) |  | 836.26(498.3 to 1319.74) | 2.82(1.69 to 4.44) |  | 1.14(0.7 to 1.57) |
| **Denmark** | Both | 194.37(156 to 238.33) | 9.67(7.76 to 11.87) |  | 149.96(114.19 to 192.45) | 7.96(6.06 to 10.21) |  | -0.28(-0.51 to -0.04) |
| **Djibouti** | Both | 3.15(1.75 to 5.21) | 2.38(1.33 to 3.9) |  | 22.34(11.69 to 38.94) | 4.09(2.14 to 7.13) |  | 1.78(1.6 to 1.96) |
| **Dominica** | Both | 1.03(0.7 to 1.46) | 4.33(2.94 to 6.11) |  | 1.95(1.22 to 2.88) | 7.47(4.65 to 11.04) |  | 2.3(1.97 to 2.63) |
| **Dominican Republic** | Both | 84.91(57.51 to 117.81) | 3.42(2.33 to 4.73) |  | 211.81(126.69 to 328.85) | 4.74(2.84 to 7.35) |  | 1.3(1.06 to 1.54) |
| **Ecuador** | Both | 78.9(61.86 to 100.23) | 2.28(1.79 to 2.9) |  | 298.65(197.49 to 423.05) | 4.23(2.8 to 5.99) |  | 2.24(1.89 to 2.6) |
| **Egypt** | Both | 671.51(466.01 to 940.65) | 3.59(2.5 to 5.02) |  | 3131.62(2024.68 to 4542.32) | 7.6(4.91 to 11.02) |  | 2.19(2.05 to 2.32) |
| **El Salvador** | Both | 44.17(32.16 to 58.75) | 2.68(1.96 to 3.55) |  | 179.8(116.97 to 261.2) | 7.66(5 to 11.12) |  | 3.45(3.06 to 3.83) |
| **Equatorial Guinea** | Both | 2.57(1.49 to 4.21) | 2.1(1.22 to 3.43) |  | 29.51(14.29 to 53.86) | 5.25(2.57 to 9.53) |  | 3.09(2.83 to 3.34) |
| **Eritrea** | Both | 25.74(15.53 to 40.37) | 2.58(1.56 to 4.04) |  | 106.31(60.47 to 171.75) | 4.31(2.46 to 6.96) |  | 1.59(1.48 to 1.7) |
| **Estonia** | Both | 35.26(27.32 to 44.83) | 5.63(4.36 to 7.17) |  | 22.95(16.6 to 30.46) | 4.64(3.35 to 6.17) |  | -0.63(-1.01 to -0.25) |
| **Eswatini** | Both | 5.94(3.51 to 9.49) | 2.65(1.57 to 4.22) |  | 21.6(10.04 to 39.12) | 4.69(2.19 to 8.48) |  | 2.04(1.6 to 2.49) |
| **Ethiopia** | Both | 469.56(261.49 to 765.07) | 3.11(1.74 to 5.07) |  | 1755.8(1257.68 to 2406.23) | 4.62(3.34 to 6.3) |  | 1.06(0.78 to 1.34) |
| **Fiji** | Both | 25.99(16.21 to 39.74) | 8.91(5.58 to 13.57) |  | 33.16(19.88 to 53.15) | 9.15(5.48 to 14.68) |  | -0.14(-0.42 to 0.15) |
| **Finland** | Both | 170.62(134.7 to 211.25) | 8(6.31 to 9.91) |  | 148.82(112.77 to 190.61) | 7.82(5.92 to 10.03) |  | 0.07(-0.08 to 0.23) |
| **France** | Both | 2027.51(1587.88 to 2528.43) | 8.6(6.73 to 10.73) |  | 2634.28(2022.38 to 3291.22) | 12.2(9.35 to 15.28) |  | 1.25(1.02 to 1.47) |
| **Gabon** | Both | 9.3(5.5 to 14.29) | 2.91(1.73 to 4.47) |  | 35.06(19.3 to 59.34) | 5.34(2.95 to 9) |  | 1.71(1.56 to 1.86) |
| **Gambia** | Both | 2.43(1.38 to 3.91) | 0.84(0.48 to 1.35) |  | 14.32(8.19 to 23.49) | 1.78(1.03 to 2.91) |  | 2.14(1.83 to 2.46) |
| **Georgia** | Both | 225.28(189.99 to 266.65) | 10.38(8.76 to 12.28) |  | 88.09(70.59 to 108.59) | 6.54(5.24 to 8.07) |  | -1.48(-1.95 to -1.01) |
| **Germany** | Both | 2695.04(2143.36 to 3296.98) | 8.44(6.72 to 10.33) |  | 2769.85(2177.89 to 3477.15) | 9.26(7.27 to 11.64) |  | 0.28(0.11 to 0.45) |
| **Ghana** | Both | 184(111.28 to 289.23) | 3.83(2.33 to 5.99) |  | 679.95(391.12 to 1088.19) | 5.25(3.03 to 8.36) |  | 0.73(0.59 to 0.87) |
| **Greece** | Both | 399.77(327.67 to 480.91) | 10.44(8.55 to 12.56) |  | 277.82(225 to 336.59) | 8.23(6.65 to 9.98) |  | -0.7(-0.89 to -0.51) |
| **Greenland** | Both | 1.46(0.97 to 2.15) | 5.52(3.68 to 8.08) |  | 0.96(0.54 to 1.56) | 4.3(2.44 to 6.96) |  | -1.03(-1.24 to -0.82) |
| **Grenada** | Both | 2.31(1.74 to 3.02) | 8.02(6.07 to 10.47) |  | 3.6(2.59 to 4.81) | 9.69(6.98 to 12.92) |  | 0.23(-0.06 to 0.51) |
| **Guam** | Both | 2.74(1.86 to 3.87) | 4.49(3.05 to 6.35) |  | 3.92(2.88 to 5.23) | 7.29(5.37 to 9.73) |  | 1.89(1.29 to 2.5) |
| **Guatemala** | Both | 47.13(38.52 to 57.07) | 1.93(1.58 to 2.34) |  | 241.44(182.8 to 311.23) | 4.13(3.13 to 5.33) |  | 2.34(2 to 2.68) |
| **Guinea** | Both | 29.87(18.11 to 47) | 1.68(1.02 to 2.63) |  | 125.63(68.3 to 211.28) | 2.98(1.63 to 5) |  | 1.87(1.79 to 1.95) |
| **Guinea-Bissau** | Both | 8.3(4.88 to 13.39) | 2.84(1.68 to 4.57) |  | 30.76(17.89 to 49.16) | 4.29(2.51 to 6.86) |  | 1.42(1.36 to 1.47) |
| **Guyana** | Both | 10.21(7.3 to 13.71) | 3.63(2.61 to 4.87) |  | 19.58(12.24 to 29.09) | 7.17(4.49 to 10.65) |  | 2.15(1.73 to 2.57) |
| **Haiti** | Both | 84.68(37.68 to 160.38) | 3.98(1.78 to 7.51) |  | 263.58(135.59 to 463.38) | 4.95(2.55 to 8.69) |  | 0.9(0.81 to 0.99) |
| **Honduras** | Both | 29.79(18.8 to 44.69) | 2.17(1.37 to 3.24) |  | 114.71(57.57 to 204.57) | 3.06(1.54 to 5.45) |  | 0.65(0.37 to 0.92) |
| **Hungary** | Both | 314.6(244.07 to 393.16) | 7.23(5.61 to 9.05) |  | 238.09(177.56 to 308.47) | 7.39(5.5 to 9.59) |  | -0.15(-0.4 to 0.1) |
| **Iceland** | Both | 9.95(7.6 to 12.79) | 9.46(7.23 to 12.16) |  | 11(8.38 to 14.38) | 8.42(6.42 to 11.01) |  | 0.15(-0.25 to 0.55) |
| **India** | Both | 5825.88(4838.79 to 6909.09) | 1.91(1.59 to 2.26) |  | 22766.3(18584.06 to 27701.25) | 3.82(3.12 to 4.64) |  | 2.34(2.24 to 2.45) |
| **Indonesia** | Both | 1942.6(1306.72 to 2813.05) | 2.88(1.95 to 4.16) |  | 5526.63(3959.94 to 7760.97) | 4.67(3.35 to 6.57) |  | 1.36(1.23 to 1.48) |
| **Iran (Islamic Republic of)** | Both | 771.22(590.69 to 996.89) | 4.44(3.41 to 5.73) |  | 4687.67(3761.71 to 5712.7) | 10.6(8.48 to 12.97) |  | 3.06(2.76 to 3.36) |
| **Iraq** | Both | 214.76(134.3 to 327.79) | 3.91(2.46 to 5.96) |  | 1299.52(799.74 to 2093.09) | 8.32(5.13 to 13.34) |  | 2.46(2.33 to 2.6) |
| **Ireland** | Both | 130.74(101.69 to 162.37) | 9.86(7.66 to 12.24) |  | 156.31(118.36 to 201.14) | 8.74(6.6 to 11.28) |  | 0.48(0.2 to 0.76) |
| **Israel** | Both | 183.74(147.33 to 227.98) | 9.79(7.85 to 12.16) |  | 260.31(202.82 to 329.42) | 7.72(6.01 to 9.78) |  | -0.22(-0.47 to 0.02) |
| **Italy** | Both | 2505.4(2132.4 to 2961.59) | 11.86(10.1 to 14.02) |  | 1628.91(1383.71 to 1909.04) | 8.9(7.55 to 10.44) |  | -1.04(-1.22 to -0.85) |
| **Jamaica** | Both | 37.61(28.75 to 48.79) | 4.81(3.68 to 6.23) |  | 121.04(76.98 to 176.96) | 10.26(6.53 to 15) |  | 2.03(1.57 to 2.48) |
| **Japan** | Both | 3005.5(2584.13 to 3510.86) | 6.37(5.47 to 7.44) |  | 2921.28(2490.73 to 3330.6) | 7.75(6.61 to 8.85) |  | 0.62(0.39 to 0.85) |
| **Jordan** | Both | 48.72(28.7 to 76.15) | 4.73(2.8 to 7.38) |  | 364.22(206.02 to 594.85) | 7.33(4.15 to 11.97) |  | 1.53(1.01 to 2.06) |
| **Kazakhstan** | Both | 416.11(350.05 to 489.68) | 6.21(5.23 to 7.31) |  | 319.9(260.21 to 386.72) | 4.01(3.27 to 4.85) |  | -0.64(-0.87 to -0.42) |
| **Kenya** | Both | 132.46(86.32 to 194.49) | 2.07(1.35 to 3.03) |  | 818.49(487.77 to 1279.08) | 4.49(2.69 to 6.98) |  | 2.48(2.4 to 2.55) |
| **Kiribati** | Both | 1.43(0.91 to 2.15) | 5.37(3.44 to 8.02) |  | 3.49(2.08 to 5.64) | 7.36(4.39 to 11.87) |  | 0.99(0.93 to 1.05) |
| **Kuwait** | Both | 30.5(22.44 to 40.48) | 3.34(2.46 to 4.43) |  | 249.19(183.49 to 326.79) | 8.63(6.32 to 11.37) |  | 0.78(-0.11 to 1.68) |
| **Kyrgyzstan** | Both | 75.25(57.03 to 96.43) | 4.68(3.55 to 5.98) |  | 109.53(79.03 to 148.39) | 3.99(2.88 to 5.41) |  | -0.73(-1.01 to -0.45) |
| **Lao People's Democratic Republic** | Both | 26.28(11.81 to 50.06) | 2.07(0.94 to 3.93) |  | 123.24(72.47 to 199.67) | 4(2.36 to 6.46) |  | 2.25(2.17 to 2.33) |
| **Latvia** | Both | 50.55(39.23 to 64.15) | 4.97(3.86 to 6.31) |  | 25.76(18.64 to 34.47) | 3.75(2.71 to 5.02) |  | -1.22(-1.55 to -0.89) |
| **Lebanon** | Both | 59.81(32.98 to 97.93) | 5.95(3.28 to 9.76) |  | 264.44(166.69 to 396.15) | 9.9(6.27 to 14.79) |  | 1.92(1.73 to 2.12) |
| **Lesotho** | Both | 7.98(3.64 to 15.1) | 1.75(0.8 to 3.32) |  | 26.49(13.88 to 44.4) | 3.65(1.92 to 6.12) |  | 3.42(2.89 to 3.94) |
| **Liberia** | Both | 11.74(6.89 to 18.88) | 1.46(0.86 to 2.34) |  | 64.34(34.39 to 111.27) | 3.23(1.72 to 5.58) |  | 2.42(2.23 to 2.61) |
| **Libya** | Both | 32.99(19.73 to 51.12) | 2.6(1.56 to 4.02) |  | 195.17(110.49 to 316.38) | 5.8(3.28 to 9.41) |  | 2.54(2.2 to 2.89) |
| **Lithuania** | Both | 87.58(67.26 to 110.69) | 6.24(4.79 to 7.88) |  | 46.28(33.26 to 62.38) | 5.02(3.61 to 6.77) |  | -0.4(-0.74 to -0.06) |
| **Luxembourg** | Both | 16.3(13.41 to 19.71) | 9.75(8.02 to 11.79) |  | 17.61(13.83 to 22.31) | 6.65(5.22 to 8.43) |  | -1.49(-1.85 to -1.13) |
| **Madagascar** | Both | 110.49(65.49 to 173.6) | 3.05(1.82 to 4.78) |  | 431.63(255.73 to 673.63) | 4.47(2.66 to 6.97) |  | 1.09(0.89 to 1.28) |
| **Malawi** | Both | 57.29(35.45 to 88.17) | 2.02(1.25 to 3.09) |  | 280.81(157.63 to 471.43) | 4.32(2.45 to 7.2) |  | 2.62(2.27 to 2.98) |
| **Malaysia** | Both | 281.65(186.94 to 401.06) | 4.22(2.81 to 6) |  | 1061.77(704.91 to 1547.08) | 7.35(4.88 to 10.7) |  | 1.85(1.7 to 2) |
| **Maldives** | Both | 0.91(0.31 to 1.96) | 1.54(0.53 to 3.33) |  | 6.43(3.87 to 10.05) | 1.85(1.11 to 2.9) |  | 0.5(-0.22 to 1.23) |
| **Mali** | Both | 53.57(34.22 to 80.53) | 2.18(1.4 to 3.27) |  | 194.61(105.59 to 321.34) | 2.96(1.61 to 4.86) |  | 0.86(0.73 to 0.99) |
| **Malta** | Both | 13.05(10.28 to 16.26) | 8.41(6.62 to 10.5) |  | 13.75(10.41 to 18.05) | 8.31(6.28 to 10.92) |  | 0.91(0.5 to 1.33) |
| **Marshall Islands** | Both | 0.69(0.39 to 1.13) | 4.77(2.7 to 7.82) |  | 1.72(0.83 to 3.11) | 7.44(3.6 to 13.44) |  | 1.24(1.14 to 1.34) |
| **Mauritania** | Both | 9.64(5.23 to 15.94) | 1.51(0.82 to 2.49) |  | 47.72(27.7 to 77.76) | 3.41(1.99 to 5.54) |  | 2.51(2.37 to 2.64) |
| **Mauritius** | Both | 14.51(11.89 to 17.58) | 2.98(2.45 to 3.62) |  | 40.26(32.22 to 49.27) | 8.49(6.8 to 10.39) |  | 2.25(1.85 to 2.66) |
| **Mexico** | Both | 1402.64(1327.01 to 1485.58) | 4.88(4.62 to 5.16) |  | 3838.48(3129.61 to 4558.72) | 7.43(6.06 to 8.82) |  | 1.02(0.86 to 1.18) |
| **Micronesia (Federated States of)** | Both | 1.81(0.98 to 3.04) | 5.27(2.84 to 8.83) |  | 2.76(1.53 to 4.45) | 7.38(4.1 to 11.85) |  | 0.95(0.86 to 1.04) |
| **Monaco** | Both | 1.67(1.11 to 2.4) | 15.69(10.37 to 22.53) |  | 2.79(1.61 to 4.38) | 26.66(15.36 to 42.04) |  | 1.99(1.74 to 2.24) |
| **Mongolia** | Both | 7.63(4.92 to 11.26) | 1.13(0.73 to 1.67) |  | 25.63(16.62 to 37.22) | 1.79(1.16 to 2.6) |  | 1.53(1.37 to 1.69) |
| **Montenegro** | Both | 22.39(16.32 to 30.82) | 8.78(6.4 to 12.08) |  | 19.78(13.71 to 27.35) | 8.56(5.92 to 11.86) |  | 0.19(-0.17 to 0.55) |
| **Morocco** | Both | 166.62(95.03 to 269.22) | 1.91(1.09 to 3.08) |  | 746.95(429.51 to 1276) | 4.96(2.85 to 8.47) |  | 3(2.78 to 3.21) |
| **Mozambique** | Both | 121.15(75.08 to 186.66) | 2.98(1.85 to 4.57) |  | 487.53(267.11 to 822.04) | 5.06(2.81 to 8.48) |  | 1.85(1.74 to 1.97) |
| **Myanmar** | Both | 830.27(430.87 to 1399.33) | 5.59(2.92 to 9.39) |  | 1520.49(938.08 to 2321.7) | 7.02(4.34 to 10.71) |  | 0.43(0.29 to 0.57) |
| **Namibia** | Both | 11.76(7.27 to 18.21) | 2.78(1.72 to 4.29) |  | 53.02(29.08 to 91.42) | 5.63(3.1 to 9.67) |  | 2.34(2.25 to 2.44) |
| **Nauru** | Both | 0.28(0.13 to 0.52) | 7.58(3.54 to 14.12) |  | 0.42(0.19 to 0.79) | 9.88(4.52 to 18.55) |  | 0.69(0.51 to 0.86) |
| **Nepal** | Both | 85.54(48.61 to 139.17) | 1.37(0.78 to 2.23) |  | 335.95(188.76 to 573.79) | 2.84(1.61 to 4.82) |  | 2.5(2.34 to 2.66) |
| **Netherlands** | Both | 678.85(549.71 to 818.21) | 10.72(8.68 to 12.93) |  | 597.23(455.74 to 763.55) | 10.62(8.1 to 13.59) |  | 0.15(0 to 0.29) |
| **New Zealand** | Both | 153.18(125.03 to 186.08) | 11.03(9 to 13.39) |  | 172.51(138.86 to 210.95) | 8.98(7.24 to 10.98) |  | -0.4(-0.59 to -0.2) |
| **Nicaragua** | Both | 33.33(22.77 to 46.62) | 2.9(1.99 to 4.03) |  | 127.06(78.99 to 192.83) | 4.62(2.87 to 7) |  | 1.82(1.64 to 2.01) |
| **Niger** | Both | 26.77(15.13 to 44.26) | 1.22(0.69 to 2) |  | 93.44(48.63 to 164.37) | 1.45(0.76 to 2.54) |  | 0.62(0.52 to 0.73) |
| **Nigeria** | Both | 619.7(414.04 to 910.6) | 2.28(1.53 to 3.34) |  | 3171.79(1693.63 to 5347.48) | 4.54(2.43 to 7.61) |  | 2.18(2.01 to 2.36) |
| **Niue** | Both | 0.05(0.03 to 0.08) | 6.82(4.03 to 11.21) |  | 0.08(0.05 to 0.12) | 14.16(9.01 to 21.32) |  | 1.01(0.55 to 1.47) |
| **North Macedonia** | Both | 51.34(38.61 to 66.11) | 6.45(4.85 to 8.3) |  | 56.48(38.68 to 79.44) | 6.08(4.16 to 8.56) |  | 0.03(-0.29 to 0.36) |
| **Northern Mariana Islands** | Both | 2.01(1.11 to 3.22) | 8.15(4.52 to 13.01) |  | 1.41(0.9 to 2.06) | 8.2(5.24 to 12.02) |  | -0.45(-0.95 to 0.05) |
| **Norway** | Both | 117.69(102.8 to 136) | 7.03(6.14 to 8.13) |  | 102.46(84.96 to 122.46) | 5.19(4.3 to 6.2) |  | -0.98(-1.33 to -0.63) |
| **Oman** | Both | 4.95(2.83 to 8.09) | 0.58(0.33 to 0.95) |  | 34.5(21.13 to 54.42) | 1.12(0.69 to 1.77) |  | 2.36(1.6 to 3.13) |
| **Pakistan** | Both | 1122.34(731.31 to 1615.48) | 3.28(2.15 to 4.68) |  | 7065.22(4403.78 to 10804.13) | 7.61(4.76 to 11.59) |  | 2.39(2.23 to 2.56) |
| **Palau** | Both | 0.75(0.41 to 1.24) | 11.32(6.27 to 18.56) |  | 0.71(0.43 to 1.1) | 11.11(6.59 to 17.33) |  | -0.19(-0.35 to -0.03) |
| **Palestine** | Both | 34.72(19.14 to 57.21) | 6.35(3.53 to 10.36) |  | 195.33(130.52 to 284.04) | 10.61(7.13 to 15.33) |  | 1.6(1.36 to 1.84) |
| **Panama** | Both | 40.77(30.93 to 52.71) | 4.7(3.57 to 6.07) |  | 200.65(140.01 to 282.61) | 12.49(8.71 to 17.58) |  | 3.37(3.12 to 3.63) |
| **Papua New Guinea** | Both | 63.53(33.15 to 104.84) | 4.55(2.39 to 7.5) |  | 217.22(124.94 to 351.14) | 5.46(3.15 to 8.8) |  | 0.25(-0.01 to 0.51) |
| **Paraguay** | Both | 46.06(30.7 to 66.79) | 3.35(2.24 to 4.84) |  | 155.22(95.02 to 244.18) | 5.18(3.17 to 8.14) |  | 1.41(1.28 to 1.54) |
| **Peru** | Both | 219.5(153.54 to 308.87) | 2.98(2.09 to 4.17) |  | 661.01(406.2 to 1028.14) | 4.43(2.72 to 6.89) |  | 1.16(0.92 to 1.4) |
| **Philippines** | Both | 982.38(826.75 to 1164.47) | 4.55(3.83 to 5.38) |  | 2906.46(2182.07 to 3821.27) | 6.61(4.97 to 8.7) |  | 1.24(1.08 to 1.4) |
| **Poland** | Both | 859.86(752.58 to 972.61) | 5(4.38 to 5.66) |  | 960.52(774.06 to 1178.97) | 6.04(4.86 to 7.41) |  | 0.57(0.35 to 0.78) |
| **Portugal** | Both | 351.05(274.23 to 439.65) | 9.43(7.37 to 11.81) |  | 374.28(285.99 to 478.27) | 10.94(8.34 to 14) |  | 0.02(-0.33 to 0.36) |
| **Puerto Rico** | Both | 111.52(85.89 to 142.11) | 8.24(6.34 to 10.49) |  | 86.39(61.29 to 116.98) | 7.99(5.67 to 10.83) |  | 0.13(-0.26 to 0.53) |
| **Qatar** | Both | 12.87(7.87 to 19.67) | 4.34(2.65 to 6.65) |  | 144.1(86.09 to 232.69) | 6.02(3.57 to 9.79) |  | 0.24(-0.45 to 0.94) |
| **Republic of Korea** | Both | 608.91(439.04 to 800.86) | 3.03(2.19 to 3.98) |  | 1432.62(995.31 to 2009.63) | 7.5(5.2 to 10.55) |  | 3.05(2.65 to 3.46) |
| **Republic of Moldova** | Both | 107.73(88.64 to 128.92) | 5.53(4.55 to 6.61) |  | 73.18(57 to 93.05) | 4.4(3.42 to 5.59) |  | -0.63(-0.96 to -0.3) |
| **Romania** | Both | 501.93(392.6 to 632.04) | 5.5(4.3 to 6.93) |  | 400.06(293.85 to 525.78) | 6.28(4.61 to 8.27) |  | 0.68(0.41 to 0.95) |
| **Russian Federation** | Both | 3348.38(3203.81 to 3507.95) | 5.01(4.8 to 5.25) |  | 3753.1(3273.66 to 4200.5) | 6.01(5.24 to 6.72) |  | 0.55(0.27 to 0.84) |
| **Rwanda** | Both | 93.31(51.84 to 152.76) | 4.06(2.27 to 6.64) |  | 248.55(140.6 to 417.41) | 4.9(2.79 to 8.2) |  | 0.18(-0.08 to 0.45) |
| **Saint Kitts and Nevis** | Both | 1.78(1.45 to 2.17) | 11.56(9.42 to 14.07) |  | 1.26(0.82 to 1.74) | 5.03(3.28 to 6.92) |  | -2.7(-3.43 to -1.96) |
| **Saint Lucia** | Both | 3.06(2.51 to 3.74) | 6.93(5.68 to 8.45) |  | 5.6(4.16 to 7.38) | 7.86(5.84 to 10.36) |  | 0.67(0.32 to 1.03) |
| **Saint Vincent and the Grenadines** | Both | 2.81(2.26 to 3.47) | 7.73(6.22 to 9.52) |  | 3.96(3 to 5.05) | 9.42(7.13 to 12.04) |  | 0.1(-0.18 to 0.39) |
| **Samoa** | Both | 1.4(0.85 to 2.18) | 2.93(1.79 to 4.55) |  | 2.99(1.64 to 4.86) | 4.35(2.38 to 7.05) |  | 1.15(1.01 to 1.29) |
| **San Marino** | Both | 0.72(0.46 to 1.05) | 7.82(4.96 to 11.45) |  | 0.6(0.29 to 1.05) | 6.31(3.03 to 10.99) |  | 0.28(-0.22 to 0.78) |
| **Sao Tome and Principe** | Both | 0.56(0.3 to 0.91) | 1.86(1.01 to 2.97) |  | 3.14(1.65 to 5.49) | 3.88(2.05 to 6.76) |  | 2.21(1.85 to 2.56) |
| **Saudi Arabia** | Both | 97.88(59.26 to 153.56) | 1.73(1.05 to 2.71) |  | 1067.3(634.35 to 1671.84) | 4.49(2.66 to 7.06) |  | 3.32(3.14 to 3.5) |
| **Senegal** | Both | 41.36(24.45 to 65.01) | 1.94(1.15 to 3.03) |  | 175.21(95.73 to 298.83) | 3.3(1.81 to 5.59) |  | 1.68(1.5 to 1.85) |
| **Serbia** | Both | 246.4(171.66 to 345.48) | 6.42(4.47 to 9.01) |  | 236.15(156.94 to 335.3) | 6.81(4.51 to 9.71) |  | 0.27(0.11 to 0.43) |
| **Seychelles** | Both | 1.17(0.78 to 1.69) | 4.39(2.92 to 6.32) |  | 3.45(2.29 to 4.96) | 7.95(5.26 to 11.44) |  | 1.43(1.19 to 1.67) |
| **Sierra Leone** | Both | 20.14(10.29 to 35.12) | 1.49(0.76 to 2.58) |  | 91.26(50.16 to 155.55) | 2.97(1.64 to 5.06) |  | 2.52(2.38 to 2.65) |
| **Singapore** | Both | 96.81(75.67 to 123.11) | 6.3(4.93 to 8) |  | 162.78(125.12 to 207.09) | 6.3(4.81 to 8.06) |  | 0.03(-0.3 to 0.37) |
| **Slovakia** | Both | 111.97(80.77 to 150.95) | 4.91(3.53 to 6.63) |  | 117.23(77.23 to 166.62) | 5.33(3.5 to 7.61) |  | 0.29(0.13 to 0.45) |
| **Slovenia** | Both | 50.56(39.19 to 64.77) | 5.88(4.56 to 7.54) |  | 43.38(31.24 to 58.6) | 5.96(4.27 to 8.07) |  | 0.24(-0.07 to 0.54) |
| **Solomon Islands** | Both | 2.35(1.02 to 4.15) | 2.43(1.04 to 4.29) |  | 12.55(6.97 to 20.28) | 5.18(2.88 to 8.36) |  | 2.33(2.15 to 2.51) |
| **Somalia** | Both | 47.43(26.28 to 77.94) | 1.87(1.04 to 3.08) |  | 135.06(75.7 to 225.93) | 2.09(1.17 to 3.49) |  | 0.1(-0.06 to 0.27) |
| **South Africa** | Both | 726.89(593.66 to 881.81) | 5.45(4.46 to 6.59) |  | 1373.28(1045.18 to 1750.59) | 5.21(3.96 to 6.65) |  | 0.05(-0.76 to 0.87) |
| **South Sudan** | Both | 33.54(18.86 to 55.68) | 1.95(1.1 to 3.21) |  | 89.1(50.41 to 143.75) | 3.1(1.75 to 5) |  | 1.57(1.15 to 1.99) |
| **Spain** | Both | 1494.4(1202.22 to 1836.39) | 10.62(8.55 to 13.04) |  | 1238.2(969.46 to 1584.59) | 8.11(6.33 to 10.41) |  | -1.2(-1.37 to -1.04) |
| **Sri Lanka** | Both | 188.42(119.82 to 281.6) | 2.71(1.73 to 4.04) |  | 384.26(211.88 to 614.57) | 4.64(2.56 to 7.43) |  | 1.86(1.75 to 1.97) |
| **Sudan** | Both | 100.16(45.56 to 190.64) | 1.65(0.75 to 3.13) |  | 647.17(285.14 to 1161.96) | 4.11(1.82 to 7.37) |  | 3.19(3.05 to 3.34) |
| **Suriname** | Both | 5.1(3.27 to 7.28) | 3.95(2.55 to 5.6) |  | 12.42(7.66 to 18.68) | 5.7(3.52 to 8.58) |  | 1.32(0.98 to 1.66) |
| **Sweden** | Both | 251.47(205.69 to 305.61) | 7.98(6.52 to 9.71) |  | 206.33(155.18 to 267.48) | 5.58(4.2 to 7.23) |  | -0.55(-0.86 to -0.25) |
| **Switzerland** | Both | 206.59(159.02 to 261.81) | 7.2(5.55 to 9.13) |  | 196.86(149.27 to 252.26) | 5.94(4.49 to 7.63) |  | -0.91(-1.48 to -0.33) |
| **Syrian Arab Republic** | Both | 147.49(88.06 to 233.57) | 4.09(2.45 to 6.49) |  | 398.43(253.43 to 615.89) | 9.34(5.88 to 14.55) |  | 2.35(2.14 to 2.57) |
| **Taiwan (Province of China)** | Both | 517.2(408.12 to 631.41) | 5.46(4.31 to 6.67) |  | 726.44(548.68 to 940.66) | 7.97(6.01 to 10.34) |  | 0.96(0.66 to 1.26) |
| **Tajikistan** | Both | 65.63(45.36 to 89.54) | 3.94(2.73 to 5.35) |  | 136.43(68.92 to 238.37) | 3.34(1.7 to 5.82) |  | -0.9(-1.25 to -0.56) |
| **Thailand** | Both | 804.73(512.88 to 1178.36) | 3.44(2.2 to 5.02) |  | 2497.56(1591.22 to 3767.02) | 10.49(6.66 to 15.84) |  | 3.35(2.81 to 3.88) |
| **Timor-Leste** | Both | 3.11(1.49 to 5.63) | 1.13(0.54 to 2.04) |  | 11.47(6.65 to 18.45) | 2.69(1.57 to 4.31) |  | 3.01(2.37 to 3.65) |
| **Togo** | Both | 26.02(15.73 to 40.92) | 2.46(1.49 to 3.83) |  | 116.99(64.82 to 198.71) | 3.94(2.19 to 6.68) |  | 1.61(1.35 to 1.87) |
| **Tokelau** | Both | 0.04(0.02 to 0.06) | 7.12(3.93 to 11.63) |  | 0.07(0.04 to 0.1) | 14.18(8.98 to 21.21) |  | 1.16(0.73 to 1.6) |
| **Tonga** | Both | 2.3(1.44 to 3.46) | 8.58(5.44 to 12.8) |  | 3.43(2.08 to 5.34) | 10.21(6.19 to 15.82) |  | 0.27(0.09 to 0.45) |
| **Trinidad and Tobago** | Both | 26.47(21.79 to 32.11) | 5.66(4.66 to 6.86) |  | 55.96(38.16 to 79.17) | 9.27(6.32 to 13.11) |  | 1.32(1.13 to 1.5) |
| **Tunisia** | Both | 93.89(60.61 to 138.81) | 3.37(2.18 to 4.97) |  | 361.3(210.6 to 570.57) | 7.09(4.12 to 11.22) |  | 2.32(2.08 to 2.56) |
| **Turkmenistan** | Both | 52.94(44.04 to 63.18) | 4.08(3.4 to 4.86) |  | 93.63(66.02 to 131.43) | 4.52(3.18 to 6.34) |  | 0.68(0.32 to 1.04) |
| **Tuvalu** | Both | 0.19(0.1 to 0.32) | 5.46(2.87 to 9.26) |  | 0.3(0.17 to 0.5) | 6.68(3.77 to 10.95) |  | 0.32(0.19 to 0.46) |
| **Türkiye** | Both | 305.61(184.32 to 471.76) | 1.48(0.89 to 2.27) |  | 3270.68(2125.35 to 4717.73) | 9.64(6.25 to 13.93) |  | 8.67(7.5 to 9.85) |
| **Uganda** | Both | 109.94(62.23 to 178.29) | 2.37(1.35 to 3.81) |  | 692.68(401.86 to 1107.38) | 5.4(3.16 to 8.55) |  | 1.69(1.32 to 2.06) |
| **Ukraine** | Both | 1620.55(1286.34 to 2013.46) | 7.69(6.1 to 9.56) |  | 837.84(438.3 to 1414.33) | 4.52(2.36 to 7.63) |  | -2.29(-2.52 to -2.06) |
| **United Arab Emirates** | Both | 27.39(15.8 to 43.81) | 2.4(1.38 to 3.84) |  | 246.05(147.14 to 383.55) | 4.18(2.36 to 6.76) |  | 1.13(0.59 to 1.67) |
| **United Kingdom** | Both | 2350.79(2237.09 to 2468.02) | 11.12(10.58 to 11.67) |  | 2214.29(2080.66 to 2354.25) | 9.01(8.46 to 9.58) |  | -0.44(-0.68 to -0.19) |
| **United Republic of Tanzania** | Both | 267.4(166.6 to 403.08) | 3.48(2.18 to 5.21) |  | 1070.65(606.42 to 1764.96) | 5.43(3.1 to 8.91) |  | 1.37(1.27 to 1.46) |
| **United States Virgin Islands** | Both | 3.88(2.51 to 5.64) | 9.7(6.26 to 14.14) |  | 3.09(1.68 to 5.33) | 11.98(6.5 to 20.72) |  | 0.7(0.34 to 1.06) |
| **United States of America** | Both | 13863.64(13276.58 to 14482.02) | 12.3(11.78 to 12.85) |  | 11761.23(10963.78 to 12592.33) | 9.82(9.15 to 10.51) |  | -0.84(-0.99 to -0.68) |
| **Uruguay** | Both | 75.95(59.4 to 94.93) | 6.83(5.34 to 8.53) |  | 109.75(83.86 to 140.58) | 8.87(6.77 to 11.37) |  | 0.41(0.2 to 0.62) |
| **Uzbekistan** | Both | 303.59(234.43 to 381.35) | 4.14(3.2 to 5.2) |  | 691.55(502.27 to 942.08) | 4.65(3.38 to 6.33) |  | 0.41(0.14 to 0.68) |
| **Vanuatu** | Both | 1.48(0.84 to 2.37) | 2.95(1.68 to 4.69) |  | 5.38(3.12 to 8.36) | 4.88(2.84 to 7.56) |  | 0.96(0.74 to 1.19) |
| **Venezuela (Bolivarian Republic of)** | Both | 403.43(329.88 to 489.61) | 5.69(4.65 to 6.91) |  | 1246.86(840.09 to 1747.03) | 12.02(8.09 to 16.83) |  | 2.24(1.83 to 2.65) |
| **Viet Nam** | Both | 547.21(316.21 to 863.39) | 2.3(1.34 to 3.61) |  | 1953.71(1224.73 to 2967.09) | 4.41(2.76 to 6.71) |  | 2.11(1.98 to 2.24) |
| **Yemen** | Both | 35.8(17.35 to 64.13) | 0.95(0.46 to 1.69) |  | 286.16(157.3 to 488.95) | 2.31(1.27 to 3.94) |  | 3.15(2.87 to 3.43) |
| **Zambia** | Both | 84.35(46.61 to 137.56) | 3.81(2.12 to 6.2) |  | 639.19(262.12 to 1256.51) | 9.35(3.9 to 18.19) |  | 3.15(2.57 to 3.73) |
| **Zimbabwe** | Both | 66.07(42.73 to 97.79) | 2.25(1.46 to 3.32) |  | 295.82(168.64 to 485.4) | 5.46(3.11 to 8.96) |  | 3.82(2.94 to 4.71) |

ASIR: age-standardized incidence rate; EAPC: estimated annual percentage change; CI: confidence interval; UI: uncertainty interval.

**Table S5.** The number and age-standardized rate of mortality caused by breast cancer at the national level among adolescents and young adults in 1990 and

2021, as well as time trends.

| **Characteristics** | **Sex** | **1990** | |  | **2021** | |  | **1990-2021** |
| --- | --- | --- | --- | --- | --- | --- | --- | --- |
|  |  | **Number** | **ASMR** |  | **Number** | **ASMR** |  | **EAPC** |
|  |  | **(95% UI)** | **(per 100000）** |  | **(95% UI)** | **(per 100000）** |  | **(95% CI)** |
|  |  |  | **(95% CI)** |  |  | **(95% CI)** |  |  |
| **Afghanistan** | Both | 24.8(9.03 to 52.42) | 1.24(0.44 to 2.62) |  | 128.35(49.56 to 265.39) | 1.53(0.59 to 3.16) |  | 0.71(0.49 to 0.92) |
| **Albania** | Both | 11.2(8.22 to 14.95) | 0.89(0.66 to 1.18) |  | 7.39(5.03 to 10.37) | 0.77(0.53 to 1.08) |  | -0.22(-0.57 to 0.13) |
| **Algeria** | Both | 54.26(34.59 to 79.54) | 0.71(0.46 to 1.04) |  | 138.36(86.94 to 207.45) | 0.71(0.44 to 1.06) |  | -0.19(-0.28 to -0.11) |
| **American Samoa** | Both | 0.41(0.26 to 0.62) | 2.39(1.53 to 3.59) |  | 0.66(0.43 to 0.97) | 4.03(2.61 to 5.92) |  | 1.97(1.85 to 2.08) |
| **Andorra** | Both | 0.4(0.25 to 0.62) | 1.46(0.91 to 2.23) |  | 0.33(0.18 to 0.51) | 1.01(0.57 to 1.59) |  | -0.79(-0.98 to -0.59) |
| **Angola** | Both | 37.8(22.97 to 60.66) | 1.16(0.71 to 1.86) |  | 174.58(100.26 to 273.56) | 1.77(1.02 to 2.76) |  | 1.68(1.47 to 1.89) |
| **Antigua and Barbuda** | Both | 0.55(0.46 to 0.65) | 2.31(1.93 to 2.75) |  | 0.61(0.52 to 0.71) | 1.66(1.42 to 1.94) |  | -0.24(-0.54 to 0.06) |
| **Argentina** | Both | 261.29(214.68 to 316.45) | 2.2(1.81 to 2.67) |  | 282.39(228.48 to 340.93) | 1.55(1.26 to 1.88) |  | -0.99(-1.22 to -0.77) |
| **Armenia** | Both | 43.89(41.19 to 46.45) | 3.08(2.89 to 3.25) |  | 11.99(9.87 to 14.15) | 0.89(0.74 to 1.06) |  | -3.97(-4.4 to -3.53) |
| **Australia** | Both | 112.78(94.19 to 133.29) | 1.58(1.32 to 1.87) |  | 83.63(67.77 to 103.06) | 0.83(0.67 to 1.02) |  | -2.37(-2.53 to -2.21) |
| **Austria** | Both | 47.73(39.43 to 56.95) | 1.61(1.33 to 1.92) |  | 28.21(23.03 to 34.02) | 0.86(0.7 to 1.04) |  | -1.7(-2.05 to -1.35) |
| **Azerbaijan** | Both | 59.57(46.98 to 71.73) | 2.16(1.71 to 2.61) |  | 56.72(40.8 to 75.47) | 1.15(0.83 to 1.53) |  | -2.2(-2.37 to -2.03) |
| **Bahamas** | Both | 4.02(3.38 to 4.75) | 3.89(3.28 to 4.6) |  | 6.71(4.88 to 9.16) | 4.29(3.12 to 5.85) |  | -0.3(-0.56 to -0.05) |
| **Bahrain** | Both | 2.99(2 to 4.31) | 1.07(0.72 to 1.54) |  | 8.24(5.44 to 11.79) | 1.01(0.67 to 1.45) |  | -0.68(-1.15 to -0.21) |
| **Bangladesh** | Both | 318.93(177.38 to 535.62) | 0.89(0.5 to 1.48) |  | 825.95(499.91 to 1280.33) | 1.23(0.74 to 1.9) |  | 0.94(0.77 to 1.1) |
| **Barbados** | Both | 2.85(2.42 to 3.3) | 2.62(2.23 to 3.03) |  | 2.97(2.19 to 3.92) | 2.74(2.02 to 3.62) |  | -0.17(-0.38 to 0.04) |
| **Belarus** | Both | 68.9(55.32 to 84.67) | 1.61(1.29 to 1.98) |  | 38.51(26.2 to 52.99) | 0.96(0.66 to 1.33) |  | -2.47(-2.73 to -2.21) |
| **Belgium** | Both | 84.39(70.03 to 100.47) | 2.1(1.74 to 2.5) |  | 39.54(31.73 to 47.87) | 0.99(0.8 to 1.2) |  | -2.61(-2.8 to -2.42) |
| **Belize** | Both | 0.39(0.33 to 0.47) | 0.7(0.58 to 0.83) |  | 1.9(1.56 to 2.33) | 1.12(0.92 to 1.37) |  | 1.7(1.21 to 2.2) |
| **Benin** | Both | 12.65(7.54 to 19.77) | 0.91(0.54 to 1.42) |  | 47.38(26.04 to 78.53) | 1.14(0.63 to 1.87) |  | 0.48(0.32 to 0.64) |
| **Bermuda** | Both | 0.61(0.49 to 0.77) | 2.09(1.67 to 2.62) |  | 0.28(0.21 to 0.36) | 1.28(0.94 to 1.65) |  | -1.91(-2.16 to -1.65) |
| **Bhutan** | Both | 1.38(0.76 to 2.26) | 0.7(0.38 to 1.13) |  | 3.02(1.61 to 5.17) | 0.86(0.46 to 1.47) |  | 0.41(0.29 to 0.53) |
| **Bolivia (Plurinational State of)** | Both | 28.36(15 to 47.51) | 1.35(0.72 to 2.26) |  | 61.64(37.08 to 94.27) | 1.31(0.79 to 1.99) |  | -0.4(-0.52 to -0.29) |
| **Bosnia and Herzegovina** | Both | 20.15(15.12 to 25.97) | 1.04(0.78 to 1.34) |  | 8.96(5.81 to 12.37) | 0.76(0.49 to 1.04) |  | -1.01(-1.26 to -0.75) |
| **Botswana** | Both | 6.12(2.89 to 11.23) | 1.55(0.73 to 2.84) |  | 18.59(9.07 to 32.48) | 1.64(0.8 to 2.87) |  | 0.7(0.23 to 1.17) |
| **Brazil** | Both | 813.72(751.35 to 879.73) | 1.47(1.35 to 1.58) |  | 1625.94(1502.15 to 1759.47) | 1.73(1.6 to 1.87) |  | 0.34(0.22 to 0.45) |
| **Brunei Darussalam** | Both | 2.03(1.25 to 2.99) | 1.7(1.05 to 2.5) |  | 3.66(2.42 to 5.2) | 1.58(1.04 to 2.24) |  | 0.43(0.19 to 0.68) |
| **Bulgaria** | Both | 64.81(52.45 to 78.52) | 1.99(1.61 to 2.41) |  | 36.21(28 to 45.33) | 1.46(1.13 to 1.84) |  | -0.64(-0.84 to -0.43) |
| **Burkina Faso** | Both | 56.73(33.32 to 89.94) | 2.22(1.31 to 3.51) |  | 174.57(94.78 to 285.68) | 2.39(1.3 to 3.89) |  | 0.28(0.08 to 0.48) |
| **Burundi** | Both | 27.53(16.58 to 45.22) | 1.59(0.96 to 2.6) |  | 70.37(42.83 to 112.18) | 1.53(0.93 to 2.43) |  | -0.44(-0.63 to -0.25) |
| **Cabo Verde** | Both | 1.9(1.22 to 2.88) | 2.1(1.35 to 3.15) |  | 2.73(1.63 to 4.27) | 1.05(0.62 to 1.64) |  | -1.94(-2.24 to -1.63) |
| **Cambodia** | Both | 42.41(20.97 to 77.6) | 1.37(0.68 to 2.51) |  | 130.17(80.43 to 201.65) | 1.8(1.11 to 2.78) |  | 0.88(0.83 to 0.92) |
| **Cameroon** | Both | 41.21(25.26 to 64.27) | 1.37(0.84 to 2.13) |  | 184.84(103.5 to 293.57) | 1.7(0.96 to 2.68) |  | 0.55(0.47 to 0.64) |
| **Canada** | Both | 196.84(164.44 to 232.34) | 1.57(1.32 to 1.86) |  | 137.52(110.27 to 170.09) | 1.01(0.81 to 1.25) |  | -1.43(-1.67 to -1.19) |
| **Central African Republic** | Both | 10.17(5.91 to 16.15) | 1.23(0.72 to 1.96) |  | 28.3(15.68 to 45.37) | 1.58(0.88 to 2.53) |  | 0.71(0.54 to 0.89) |
| **Chad** | Both | 13.75(7.45 to 22.83) | 0.82(0.44 to 1.36) |  | 49.88(28.79 to 79.85) | 1.07(0.62 to 1.71) |  | 1.06(0.97 to 1.15) |
| **Chile** | Both | 61.71(49.27 to 75.96) | 1.19(0.95 to 1.46) |  | 65.37(52.04 to 80.9) | 0.86(0.68 to 1.06) |  | -0.76(-1.09 to -0.43) |
| **China** | Both | 5708.56(4455.75 to 7214) | 1.14(0.89 to 1.44) |  | 4929.2(3624.75 to 6465.5) | 0.86(0.63 to 1.12) |  | -1.18(-1.33 to -1.03) |
| **Colombia** | Both | 148.39(119.04 to 181.48) | 1.23(0.99 to 1.5) |  | 269.99(199.35 to 357.08) | 1.35(0.99 to 1.78) |  | 0.5(0.12 to 0.88) |
| **Comoros** | Both | 2.39(1.26 to 3.77) | 1.84(0.99 to 2.89) |  | 7.18(4.53 to 11.37) | 2.53(1.6 to 4) |  | 0.44(-0.07 to 0.95) |
| **Congo** | Both | 12.9(6.29 to 23.28) | 1.81(0.89 to 3.27) |  | 53.65(27.51 to 93.37) | 2.61(1.34 to 4.54) |  | 1.12(0.92 to 1.32) |
| **Cook Islands** | Both | 0.24(0.14 to 0.37) | 3.59(2.14 to 5.57) |  | 0.2(0.12 to 0.31) | 3.62(2.23 to 5.49) |  | 0.39(0.1 to 0.68) |
| **Costa Rica** | Both | 11.5(9.22 to 14.3) | 1.02(0.82 to 1.27) |  | 29.19(22.4 to 37.24) | 1.43(1.09 to 1.82) |  | 1.46(1.05 to 1.88) |
| **Coted'Ivoire** | Both | 43.36(26.16 to 66.95) | 1.16(0.7 to 1.78) |  | 171.59(97.37 to 273.86) | 1.7(0.97 to 2.71) |  | 1.39(1.26 to 1.52) |
| **Croatia** | Both | 29.04(23.73 to 35.14) | 1.41(1.15 to 1.71) |  | 12.74(9.89 to 15.98) | 0.84(0.65 to 1.06) |  | -1.36(-1.69 to -1.02) |
| **Cuba** | Both | 62.18(49.77 to 76.61) | 1.51(1.21 to 1.87) |  | 35.69(26.57 to 46.55) | 0.9(0.67 to 1.17) |  | -1.56(-1.77 to -1.35) |
| **Cyprus** | Both | 3.84(2.66 to 5.49) | 1.24(0.86 to 1.77) |  | 4.78(3.3 to 6.72) | 0.72(0.49 to 1.01) |  | -1.86(-2.3 to -1.42) |
| **Czechia** | Both | 53.28(43.58 to 64.64) | 1.28(1.05 to 1.55) |  | 35.59(28 to 44.41) | 0.95(0.75 to 1.19) |  | -0.94(-1.4 to -0.48) |
| **Democratic People's Republic of Korea** | Both | 89.78(50.6 to 146.91) | 1.2(0.68 to 1.96) |  | 135.2(78.58 to 215.49) | 1.23(0.72 to 1.96) |  | 0.08(0.02 to 0.14) |
| **Democratic Republic of the Congo** | Both | 128.05(74.94 to 205.12) | 1.13(0.66 to 1.8) |  | 404.36(243.15 to 642.13) | 1.37(0.83 to 2.17) |  | 0.51(0.16 to 0.87) |
| **Denmark** | Both | 41.45(34.38 to 49.09) | 2.06(1.71 to 2.44) |  | 16.7(13.52 to 20.25) | 0.89(0.72 to 1.08) |  | -2.67(-2.98 to -2.36) |
| **Djibouti** | Both | 1.75(0.98 to 2.85) | 1.33(0.75 to 2.15) |  | 9.98(5.23 to 17.03) | 1.83(0.96 to 3.12) |  | 1.03(0.79 to 1.26) |
| **Dominica** | Both | 0.35(0.25 to 0.5) | 1.5(1.04 to 2.11) |  | 0.57(0.37 to 0.84) | 2.18(1.4 to 3.21) |  | 1.82(1.47 to 2.17) |
| **Dominican Republic** | Both | 33.42(23.29 to 45.98) | 1.36(0.95 to 1.86) |  | 60.73(37.68 to 90.52) | 1.36(0.85 to 2.03) |  | 0.36(0.18 to 0.55) |
| **Ecuador** | Both | 32.92(26.48 to 41.02) | 0.96(0.77 to 1.19) |  | 74.8(51.1 to 104.08) | 1.06(0.73 to 1.48) |  | 0.68(0.4 to 0.96) |
| **Egypt** | Both | 228.27(163.41 to 313) | 1.23(0.88 to 1.68) |  | 608.67(400.06 to 857.55) | 1.48(0.97 to 2.08) |  | 0.58(0.48 to 0.69) |
| **El Salvador** | Both | 13.44(10.25 to 17.33) | 0.82(0.63 to 1.06) |  | 28.09(19.23 to 40.53) | 1.21(0.83 to 1.75) |  | 1.25(1.07 to 1.43) |
| **Equatorial Guinea** | Both | 1.61(0.95 to 2.64) | 1.32(0.78 to 2.16) |  | 10.73(5.29 to 19.39) | 1.92(0.96 to 3.47) |  | 1.07(0.83 to 1.3) |
| **Eritrea** | Both | 16.01(9.67 to 25.16) | 1.61(0.97 to 2.53) |  | 54.81(30.71 to 88.43) | 2.23(1.25 to 3.6) |  | 1.01(0.91 to 1.11) |
| **Estonia** | Both | 8.86(7.14 to 10.93) | 1.41(1.14 to 1.74) |  | 3.16(2.36 to 4.07) | 0.63(0.47 to 0.81) |  | -2.96(-3.35 to -2.56) |
| **Eswatini** | Both | 3.09(1.81 to 4.9) | 1.39(0.81 to 2.2) |  | 9.66(4.45 to 17.51) | 2.11(0.97 to 3.81) |  | 1.58(1.06 to 2.12) |
| **Ethiopia** | Both | 302.21(169.62 to 489.89) | 2.01(1.13 to 3.26) |  | 781.13(569.26 to 1049.63) | 2.08(1.52 to 2.77) |  | -0.17(-0.39 to 0.05) |
| **Fiji** | Both | 10.34(6.65 to 15.65) | 3.57(2.31 to 5.38) |  | 13.73(8.55 to 21.24) | 3.78(2.35 to 5.86) |  | 0.25(0.04 to 0.45) |
| **Finland** | Both | 31.71(25.87 to 38.37) | 1.48(1.2 to 1.79) |  | 14.42(11.56 to 17.55) | 0.75(0.6 to 0.92) |  | -2.23(-2.45 to -2.01) |
| **France** | Both | 383.85(317.97 to 457.02) | 1.62(1.34 to 1.93) |  | 236.88(194.52 to 284.91) | 1.09(0.89 to 1.31) |  | -1.41(-1.57 to -1.24) |
| **Gabon** | Both | 5.04(3.03 to 7.73) | 1.59(0.95 to 2.43) |  | 13.6(7.67 to 22.29) | 2.08(1.18 to 3.4) |  | 0.62(0.43 to 0.81) |
| **Gambia** | Both | 1.3(0.73 to 2.11) | 0.45(0.26 to 0.73) |  | 6.23(3.67 to 10.14) | 0.78(0.46 to 1.27) |  | 1.45(1.15 to 1.76) |
| **Georgia** | Both | 66.88(59.42 to 74.76) | 3.09(2.75 to 3.45) |  | 21.84(18.38 to 25.3) | 1.61(1.35 to 1.86) |  | -1.86(-2.32 to -1.4) |
| **Germany** | Both | 590.66(492.35 to 703.08) | 1.86(1.55 to 2.21) |  | 317.3(262.36 to 380.54) | 1.05(0.87 to 1.26) |  | -1.77(-2.1 to -1.43) |
| **Ghana** | Both | 97.24(59.7 to 151.23) | 2.04(1.26 to 3.15) |  | 279.28(162.84 to 438.74) | 2.17(1.27 to 3.38) |  | 0(-0.12 to 0.12) |
| **Greece** | Both | 66.66(58.59 to 75.08) | 1.74(1.53 to 1.96) |  | 34.27(29.74 to 39.35) | 1(0.87 to 1.15) |  | -1.7(-1.89 to -1.5) |
| **Greenland** | Both | 0.55(0.37 to 0.77) | 2.07(1.41 to 2.92) |  | 0.24(0.14 to 0.37) | 1.08(0.62 to 1.65) |  | -2.46(-2.68 to -2.24) |
| **Grenada** | Both | 0.83(0.64 to 1.05) | 2.89(2.25 to 3.67) |  | 0.93(0.68 to 1.23) | 2.54(1.85 to 3.33) |  | -0.67(-1 to -0.33) |
| **Guam** | Both | 0.71(0.51 to 0.98) | 1.17(0.84 to 1.62) |  | 1.08(0.8 to 1.42) | 2.01(1.5 to 2.65) |  | 2.13(1.67 to 2.6) |
| **Guatemala** | Both | 17.63(14.95 to 20.78) | 0.73(0.62 to 0.86) |  | 54.16(42.62 to 67.36) | 0.93(0.74 to 1.16) |  | 0.72(0.35 to 1.09) |
| **Guinea** | Both | 17.67(10.84 to 27.42) | 0.99(0.61 to 1.54) |  | 62.88(33.87 to 106.11) | 1.5(0.81 to 2.52) |  | 1.38(1.31 to 1.45) |
| **Guinea-Bissau** | Both | 5.18(3.06 to 8.2) | 1.78(1.05 to 2.81) |  | 16.08(9.5 to 25.33) | 2.26(1.34 to 3.55) |  | 0.85(0.81 to 0.88) |
| **Guyana** | Both | 4.6(3.34 to 6.04) | 1.65(1.2 to 2.16) |  | 6.94(4.39 to 10.21) | 2.57(1.62 to 3.77) |  | 1.54(1.14 to 1.94) |
| **Haiti** | Both | 47.75(21.49 to 89.14) | 2.25(1.02 to 4.19) |  | 124.53(63.32 to 212.67) | 2.34(1.19 to 3.99) |  | 0.32(0.23 to 0.4) |
| **Honduras** | Both | 10.57(6.78 to 15.81) | 0.77(0.5 to 1.15) |  | 29.22(15.2 to 50.06) | 0.79(0.41 to 1.34) |  | -0.38(-0.61 to -0.14) |
| **Hungary** | Both | 84.51(68.37 to 102.16) | 1.92(1.56 to 2.33) |  | 34.86(27.43 to 43.14) | 1.07(0.84 to 1.33) |  | -1.97(-2.33 to -1.62) |
| **Iceland** | Both | 1.63(1.33 to 1.99) | 1.56(1.26 to 1.89) |  | 1.11(0.88 to 1.37) | 0.85(0.68 to 1.05) |  | -1.33(-1.86 to -0.8) |
| **India** | Both | 3116.52(2590.59 to 3712.05) | 1.02(0.85 to 1.22) |  | 8209.59(6682.58 to 10005.27) | 1.38(1.12 to 1.68) |  | 1.02(0.91 to 1.13) |
| **Indonesia** | Both | 948.7(641.94 to 1381.5) | 1.42(0.96 to 2.05) |  | 1970.37(1405.37 to 2786.35) | 1.66(1.19 to 2.35) |  | 0.35(0.2 to 0.51) |
| **Iran (Islamic Republic of)** | Both | 154.83(126.67 to 189.74) | 0.9(0.74 to 1.11) |  | 489.19(425.69 to 568.09) | 1.09(0.94 to 1.26) |  | 0.96(0.69 to 1.23) |
| **Iraq** | Both | 61.51(39.53 to 91.76) | 1.13(0.73 to 1.68) |  | 215.42(135.83 to 329.98) | 1.39(0.88 to 2.12) |  | 0.54(0.37 to 0.7) |
| **Ireland** | Both | 26.07(21.46 to 31.02) | 1.96(1.62 to 2.34) |  | 15.65(12.53 to 18.96) | 0.86(0.69 to 1.05) |  | -2.12(-2.35 to -1.89) |
| **Israel** | Both | 42.09(35.19 to 49.32) | 2.24(1.87 to 2.62) |  | 32.59(27.21 to 38.6) | 0.96(0.8 to 1.14) |  | -2.32(-2.58 to -2.07) |
| **Italy** | Both | 413.21(390.6 to 435.69) | 1.96(1.85 to 2.07) |  | 157.94(146.93 to 169.87) | 0.85(0.79 to 0.92) |  | -2.77(-2.97 to -2.57) |
| **Jamaica** | Both | 10.83(8.59 to 13.39) | 1.4(1.11 to 1.73) |  | 27.76(18.17 to 40.19) | 2.36(1.55 to 3.42) |  | 1.3(0.84 to 1.76) |
| **Japan** | Both | 445.53(429.43 to 461.25) | 0.94(0.9 to 0.97) |  | 270.29(259.97 to 281.14) | 0.71(0.68 to 0.74) |  | -1.04(-1.19 to -0.89) |
| **Jordan** | Both | 12.01(7.48 to 18.45) | 1.19(0.74 to 1.82) |  | 48.33(27.67 to 77.76) | 0.98(0.56 to 1.57) |  | -0.78(-1.27 to -0.29) |
| **Kazakhstan** | Both | 146.77(129.87 to 166.5) | 2.2(1.95 to 2.49) |  | 74.1(63.12 to 85.13) | 0.93(0.79 to 1.06) |  | -2.29(-2.57 to -2.01) |
| **Kenya** | Both | 66.11(43.41 to 97.35) | 1.04(0.69 to 1.53) |  | 339.66(204.66 to 529.66) | 1.87(1.13 to 2.9) |  | 1.98(1.91 to 2.04) |
| **Kiribati** | Both | 0.79(0.5 to 1.18) | 2.97(1.91 to 4.41) |  | 1.85(1.07 to 2.99) | 3.9(2.27 to 6.31) |  | 1.02(0.98 to 1.07) |
| **Kuwait** | Both | 4.57(3.52 to 5.76) | 0.5(0.39 to 0.63) |  | 23.05(17.85 to 29.15) | 0.78(0.6 to 0.99) |  | -0.9(-1.77 to -0.02) |
| **Kyrgyzstan** | Both | 28.74(22.13 to 35.77) | 1.8(1.39 to 2.24) |  | 29.06(21.62 to 37.43) | 1.06(0.79 to 1.37) |  | -1.99(-2.19 to -1.79) |
| **Lao People's Democratic Republic** | Both | 15.76(7.14 to 29.34) | 1.25(0.57 to 2.31) |  | 52(31.03 to 84.27) | 1.69(1.01 to 2.74) |  | 1.03(0.9 to 1.15) |
| **Latvia** | Both | 15.77(12.72 to 19.54) | 1.55(1.25 to 1.92) |  | 5.62(4.16 to 7.22) | 0.81(0.6 to 1.04) |  | -2.41(-2.77 to -2.05) |
| **Lebanon** | Both | 13.83(7.82 to 22.41) | 1.38(0.78 to 2.24) |  | 30.64(20.12 to 43.15) | 1.15(0.76 to 1.61) |  | -0.39(-0.53 to -0.25) |
| **Lesotho** | Both | 4.33(2.03 to 8.12) | 0.95(0.45 to 1.79) |  | 13.44(7.1 to 22.36) | 1.86(0.98 to 3.09) |  | 3.29(2.74 to 3.84) |
| **Liberia** | Both | 6.82(3.91 to 11.11) | 0.85(0.49 to 1.38) |  | 27.61(15.05 to 47.61) | 1.39(0.76 to 2.39) |  | 1.35(1.13 to 1.56) |
| **Libya** | Both | 8.25(5.04 to 12.51) | 0.66(0.4 to 0.99) |  | 34.11(20.4 to 54.4) | 1.01(0.6 to 1.61) |  | 1.19(0.97 to 1.41) |
| **Lithuania** | Both | 20.79(16.65 to 25.28) | 1.49(1.19 to 1.8) |  | 8.03(5.86 to 10.34) | 0.87(0.63 to 1.12) |  | -1.31(-1.72 to -0.91) |
| **Luxembourg** | Both | 3.43(2.96 to 3.95) | 2.05(1.77 to 2.36) |  | 1.88(1.58 to 2.2) | 0.7(0.59 to 0.82) |  | -3.74(-3.98 to -3.51) |
| **Madagascar** | Both | 63.76(38.28 to 99.63) | 1.77(1.07 to 2.76) |  | 207.72(125.95 to 325.23) | 2.17(1.32 to 3.39) |  | 0.53(0.35 to 0.72) |
| **Malawi** | Both | 33.8(21.13 to 50.39) | 1.2(0.75 to 1.78) |  | 131.23(75.42 to 216) | 2.04(1.18 to 3.33) |  | 1.85(1.55 to 2.15) |
| **Malaysia** | Both | 114.65(76.69 to 159.83) | 1.72(1.16 to 2.4) |  | 269.27(183.77 to 377.63) | 1.86(1.27 to 2.61) |  | 0.22(0.01 to 0.42) |
| **Maldives** | Both | 0.42(0.14 to 0.88) | 0.72(0.25 to 1.52) |  | 1.41(0.86 to 2.18) | 0.4(0.24 to 0.62) |  | -2.18(-2.74 to -1.61) |
| **Mali** | Both | 31.21(20.46 to 47.21) | 1.28(0.84 to 1.92) |  | 92.31(51.29 to 154.12) | 1.42(0.79 to 2.35) |  | 0.22(0.08 to 0.37) |
| **Malta** | Both | 2.91(2.37 to 3.49) | 1.86(1.52 to 2.24) |  | 1.71(1.37 to 2.08) | 1.02(0.82 to 1.25) |  | -1.07(-1.53 to -0.61) |
| **Marshall Islands** | Both | 0.33(0.19 to 0.54) | 2.32(1.32 to 3.76) |  | 0.81(0.39 to 1.43) | 3.48(1.69 to 6.19) |  | 1.26(1.09 to 1.43) |
| **Mauritania** | Both | 5.38(2.92 to 8.67) | 0.85(0.46 to 1.36) |  | 17.65(10.57 to 28.16) | 1.27(0.77 to 2.02) |  | 1.21(1.07 to 1.35) |
| **Mauritius** | Both | 5.01(4.29 to 5.85) | 1.04(0.89 to 1.21) |  | 9.9(8.23 to 11.57) | 2.09(1.73 to 2.44) |  | 1.29(0.9 to 1.67) |
| **Mexico** | Both | 376.04(359.05 to 395.07) | 1.32(1.26 to 1.39) |  | 634.62(519.1 to 759.78) | 1.23(1 to 1.47) |  | -0.41(-0.56 to -0.25) |
| **Micronesia (Federated States of)** | Both | 0.89(0.49 to 1.44) | 2.6(1.44 to 4.2) |  | 1.2(0.68 to 1.88) | 3.21(1.82 to 5.03) |  | 0.64(0.57 to 0.72) |
| **Monaco** | Both | 0.27(0.18 to 0.37) | 2.48(1.68 to 3.45) |  | 0.3(0.18 to 0.46) | 2.83(1.68 to 4.36) |  | 0.61(0.47 to 0.75) |
| **Mongolia** | Both | 3.81(2.5 to 5.57) | 0.57(0.38 to 0.83) |  | 8.36(5.48 to 12.04) | 0.58(0.38 to 0.84) |  | -0.16(-0.37 to 0.05) |
| **Montenegro** | Both | 4.34(3.28 to 5.83) | 1.7(1.28 to 2.29) |  | 2.85(2.05 to 3.83) | 1.22(0.88 to 1.64) |  | -1.09(-1.56 to -0.63) |
| **Morocco** | Both | 55.78(32.45 to 90.24) | 0.64(0.38 to 1.04) |  | 160.71(93.45 to 275.29) | 1.07(0.62 to 1.83) |  | 1.64(1.4 to 1.88) |
| **Mozambique** | Both | 72.32(44.55 to 108.79) | 1.78(1.1 to 2.67) |  | 244.85(136.32 to 410.45) | 2.57(1.45 to 4.28) |  | 1.4(1.27 to 1.52) |
| **Myanmar** | Both | 458.44(239.87 to 770.42) | 3.1(1.63 to 5.19) |  | 570.63(358.91 to 851.57) | 2.64(1.66 to 3.94) |  | -0.89(-1.09 to -0.69) |
| **Namibia** | Both | 6.22(3.93 to 9.4) | 1.48(0.94 to 2.23) |  | 20.85(11.78 to 34.81) | 2.22(1.26 to 3.7) |  | 1.33(1.22 to 1.43) |
| **Nauru** | Both | 0.13(0.06 to 0.23) | 3.43(1.59 to 6.24) |  | 0.18(0.08 to 0.33) | 4.21(1.94 to 7.83) |  | 0.69(0.64 to 0.74) |
| **Nepal** | Both | 49.15(28.23 to 78.7) | 0.79(0.46 to 1.26) |  | 128.78(73.59 to 214.59) | 1.1(0.63 to 1.81) |  | 1.17(0.99 to 1.35) |
| **Netherlands** | Both | 130.23(109.11 to 151.81) | 2.06(1.72 to 2.4) |  | 60.91(49.94 to 73.38) | 1.08(0.89 to 1.3) |  | -2.32(-2.49 to -2.16) |
| **New Zealand** | Both | 32.02(26.99 to 37.29) | 2.31(1.94 to 2.69) |  | 22.27(18.77 to 25.94) | 1.16(0.98 to 1.35) |  | -2.03(-2.22 to -1.85) |
| **Nicaragua** | Both | 9.27(6.51 to 12.74) | 0.82(0.57 to 1.12) |  | 21.7(14.1 to 31.95) | 0.79(0.51 to 1.16) |  | 0.12(-0.04 to 0.29) |
| **Niger** | Both | 16.12(9.06 to 27) | 0.74(0.42 to 1.23) |  | 46.89(24.74 to 81.87) | 0.74(0.39 to 1.28) |  | -0.03(-0.16 to 0.09) |
| **Nigeria** | Both | 341.08(234.89 to 489.63) | 1.26(0.87 to 1.8) |  | 1321.75(743.9 to 2183.83) | 1.91(1.08 to 3.14) |  | 1.23(1.08 to 1.39) |
| **Niue** | Both | 0.02(0.01 to 0.03) | 2.54(1.54 to 4.01) |  | 0.02(0.02 to 0.04) | 4.49(2.92 to 6.55) |  | 0.73(0.33 to 1.13) |
| **North Macedonia** | Both | 16.06(12.44 to 20.26) | 2.02(1.57 to 2.55) |  | 10.35(7.49 to 14.17) | 1.11(0.8 to 1.52) |  | -1.94(-2.18 to -1.71) |
| **Northern Mariana Islands** | Both | 0.56(0.31 to 0.88) | 2.25(1.27 to 3.58) |  | 0.39(0.25 to 0.55) | 2.24(1.46 to 3.18) |  | -0.06(-0.52 to 0.4) |
| **Norway** | Both | 24.45(22.74 to 26.27) | 1.46(1.36 to 1.57) |  | 11.14(10.15 to 12.22) | 0.56(0.51 to 0.62) |  | -3.29(-3.51 to -3.08) |
| **Oman** | Both | 1.25(0.73 to 1.98) | 0.15(0.09 to 0.23) |  | 4.45(2.84 to 6.85) | 0.14(0.09 to 0.22) |  | 0.31(-0.4 to 1.02) |
| **Pakistan** | Both | 607.84(407.43 to 868.95) | 1.79(1.2 to 2.53) |  | 3010.29(1901.65 to 4494.67) | 3.26(2.07 to 4.86) |  | 1.58(1.35 to 1.8) |
| **Palau** | Both | 0.26(0.15 to 0.42) | 3.97(2.27 to 6.31) |  | 0.23(0.14 to 0.35) | 3.52(2.17 to 5.39) |  | -0.22(-0.42 to -0.02) |
| **Palestine** | Both | 9.04(5.11 to 14.8) | 1.69(0.96 to 2.75) |  | 31.55(22.11 to 43.35) | 1.74(1.22 to 2.37) |  | 0.14(-0.05 to 0.32) |
| **Panama** | Both | 8.26(6.54 to 10.4) | 0.96(0.76 to 1.21) |  | 24.37(17.48 to 33) | 1.52(1.09 to 2.06) |  | 1.81(1.58 to 2.03) |
| **Papua New Guinea** | Both | 29.53(15.58 to 48.01) | 2.13(1.13 to 3.46) |  | 103.3(59.57 to 163.17) | 2.61(1.51 to 4.11) |  | 0.55(0.39 to 0.71) |
| **Paraguay** | Both | 16.52(11.02 to 23.43) | 1.21(0.81 to 1.71) |  | 39.8(25.48 to 61.51) | 1.33(0.85 to 2.06) |  | 0.37(0.24 to 0.5) |
| **Peru** | Both | 91.04(64.79 to 125.83) | 1.24(0.89 to 1.71) |  | 136.14(84.82 to 206.61) | 0.91(0.57 to 1.39) |  | -1.24(-1.47 to -1.02) |
| **Philippines** | Both | 419.04(360.85 to 480.13) | 1.95(1.68 to 2.23) |  | 1023(774.23 to 1326.97) | 2.34(1.77 to 3.03) |  | 0.74(0.62 to 0.86) |
| **Poland** | Both | 258.4(244.4 to 272.65) | 1.49(1.41 to 1.57) |  | 152.1(129.05 to 174.25) | 0.94(0.8 to 1.08) |  | -1.63(-1.91 to -1.35) |
| **Portugal** | Both | 76.49(62.53 to 92.9) | 2.06(1.68 to 2.5) |  | 39.64(32.04 to 47.72) | 1.15(0.93 to 1.38) |  | -2.39(-2.64 to -2.15) |
| **Puerto Rico** | Both | 25.6(20.45 to 31.21) | 1.89(1.51 to 2.31) |  | 11.88(8.72 to 15.79) | 1.1(0.8 to 1.46) |  | -1.66(-2.07 to -1.24) |
| **Qatar** | Both | 2.65(1.67 to 3.93) | 0.89(0.56 to 1.32) |  | 13.51(8.33 to 21.24) | 0.56(0.34 to 0.88) |  | -2.37(-3.03 to -1.71) |
| **Republic of Korea** | Both | 175.63(130.73 to 226.77) | 0.88(0.66 to 1.14) |  | 143.18(105.07 to 192.22) | 0.74(0.54 to 1) |  | -0.72(-0.87 to -0.58) |
| **Republic of Moldova** | Both | 35.31(30.28 to 40.96) | 1.81(1.55 to 2.1) |  | 15.52(12.7 to 18.93) | 0.92(0.76 to 1.13) |  | -1.98(-2.24 to -1.71) |
| **Romania** | Both | 166.95(134.63 to 203.33) | 1.82(1.47 to 2.21) |  | 72.65(56.04 to 93.29) | 1.13(0.87 to 1.45) |  | -1.43(-1.73 to -1.12) |
| **Russian Federation** | Both | 1029.42(987.46 to 1073.57) | 1.54(1.47 to 1.6) |  | 744.17(647.47 to 834.93) | 1.17(1.02 to 1.32) |  | -1.11(-1.28 to -0.94) |
| **Rwanda** | Both | 58.62(33.02 to 96.14) | 2.56(1.45 to 4.19) |  | 112.01(64.55 to 185.33) | 2.22(1.28 to 3.65) |  | -1(-1.25 to -0.76) |
| **Saint Kitts and Nevis** | Both | 0.71(0.59 to 0.84) | 4.64(3.86 to 5.47) |  | 0.32(0.22 to 0.43) | 1.28(0.87 to 1.72) |  | -4.16(-5.02 to -3.3) |
| **Saint Lucia** | Both | 1.07(0.91 to 1.26) | 2.44(2.07 to 2.87) |  | 1.38(1.07 to 1.75) | 1.94(1.5 to 2.45) |  | -0.45(-0.84 to -0.06) |
| **Saint Vincent and the Grenadines** | Both | 0.96(0.8 to 1.14) | 2.67(2.24 to 3.17) |  | 1.09(0.87 to 1.34) | 2.6(2.06 to 3.19) |  | -0.6(-0.89 to -0.31) |
| **Samoa** | Both | 0.57(0.35 to 0.88) | 1.2(0.74 to 1.85) |  | 1.11(0.63 to 1.73) | 1.62(0.92 to 2.52) |  | 1.08(0.97 to 1.2) |
| **San Marino** | Both | 0.11(0.07 to 0.15) | 1.16(0.78 to 1.65) |  | 0.06(0.03 to 0.11) | 0.66(0.33 to 1.14) |  | -0.87(-1.26 to -0.48) |
| **Sao Tome and Principe** | Both | 0.29(0.16 to 0.47) | 0.97(0.53 to 1.54) |  | 1.16(0.63 to 2) | 1.44(0.78 to 2.47) |  | 1.07(0.69 to 1.44) |
| **Saudi Arabia** | Both | 38.45(22.93 to 58.56) | 0.68(0.41 to 1.04) |  | 208.66(125.83 to 321.35) | 0.87(0.52 to 1.35) |  | 0.82(0.6 to 1.04) |
| **Senegal** | Both | 22.75(13.56 to 35.21) | 1.07(0.64 to 1.65) |  | 76.11(43.08 to 128.62) | 1.45(0.82 to 2.43) |  | 0.98(0.81 to 1.16) |
| **Serbia** | Both | 70.49(51.01 to 97.15) | 1.83(1.33 to 2.53) |  | 37.95(26.4 to 51.74) | 1.09(0.75 to 1.49) |  | -1.82(-2 to -1.63) |
| **Seychelles** | Both | 0.45(0.3 to 0.64) | 1.71(1.15 to 2.41) |  | 0.93(0.63 to 1.29) | 2.12(1.43 to 2.96) |  | 0.19(0 to 0.39) |
| **Sierra Leone** | Both | 11.38(5.91 to 19.52) | 0.84(0.44 to 1.44) |  | 42.14(22.35 to 69.95) | 1.38(0.74 to 2.29) |  | 1.91(1.79 to 2.03) |
| **Singapore** | Both | 22.35(18.31 to 26.96) | 1.46(1.2 to 1.76) |  | 16.63(13.6 to 20.06) | 0.63(0.51 to 0.76) |  | -2.83(-3.2 to -2.47) |
| **Slovakia** | Both | 32.23(23.75 to 42.09) | 1.41(1.04 to 1.84) |  | 19.92(13.47 to 27.69) | 0.89(0.6 to 1.25) |  | -1.5(-1.67 to -1.34) |
| **Slovenia** | Both | 10.21(8.28 to 12.48) | 1.18(0.96 to 1.45) |  | 4.58(3.42 to 5.92) | 0.62(0.46 to 0.8) |  | -2.13(-2.47 to -1.79) |
| **Solomon Islands** | Both | 1.19(0.51 to 2.09) | 1.24(0.53 to 2.18) |  | 6.07(3.39 to 9.67) | 2.52(1.41 to 4) | |  |
| **Somalia** | Both | 29.9(17.22 to 50.71) | 1.18(0.68 to 2) |  | 78.54(44.05 to 131.7) | 1.22(0.68 to 2.05) |  | -0.15(-0.31 to 0.01) |
| **South Africa** | Both | 324.64(266.64 to 390.28) | 2.45(2.02 to 2.94) |  | 497.98(381.38 to 628.31) | 1.89(1.44 to 2.38) |  | -0.67(-1.51 to 0.18) |
| **South Sudan** | Both | 19.33(10.9 to 31.7) | 1.13(0.64 to 1.84) |  | 43.5(25.11 to 69.78) | 1.51(0.87 to 2.43) |  | 0.98(0.56 to 1.4) |
| **Spain** | Both | 284.92(237.68 to 335.5) | 2.03(1.7 to 2.39) |  | 132.8(109.49 to 158.76) | 0.86(0.71 to 1.03) |  | -3.11(-3.36 to -2.87) |
| **Sri Lanka** | Both | 72.16(46.88 to 106.86) | 1.04(0.68 to 1.54) |  | 80.89(46.9 to 127.65) | 0.97(0.56 to 1.54) |  | -0.24(-0.35 to -0.13) |
| **Sudan** | Both | 38.51(17.87 to 72.89) | 0.64(0.3 to 1.21) |  | 159.62(71.66 to 278.38) | 1.02(0.46 to 1.78) |  | 1.87(1.71 to 2.02) |
| **Suriname** | Both | 2.01(1.31 to 2.83) | 1.57(1.03 to 2.2) |  | 3.92(2.44 to 5.78) | 1.8(1.12 to 2.65) |  | 0.53(0.19 to 0.87) |
| **Sweden** | Both | 44.45(37.34 to 51.84) | 1.41(1.18 to 1.64) |  | 21.98(16.94 to 27.73) | 0.59(0.46 to 0.75) |  | -2.24(-2.44 to -2.03) |
| **Switzerland** | Both | 34.16(27.71 to 41.01) | 1.19(0.97 to 1.43) |  | 19.24(15.46 to 23.57) | 0.57(0.46 to 0.7) |  | -2.64(-3.03 to -2.25) |
| **Syrian Arab Republic** | Both | 40.67(24.71 to 63.38) | 1.15(0.7 to 1.79) |  | 60.68(39.28 to 91.58) | 1.39(0.89 to 2.12) |  | 0.29(-0.06 to 0.65) |
| **Taiwan (Province of China)** | Both | 122.1(99.63 to 145.13) | 1.29(1.05 to 1.53) |  | 92.65(74.27 to 114.15) | 1.01(0.8 to 1.24) |  | -1.1(-1.3 to -0.91) |
| **Tajikistan** | Both | 27.69(19.66 to 37.04) | 1.68(1.2 to 2.24) |  | 48.44(24.98 to 82.66) | 1.19(0.62 to 2.03) |  | -1.41(-1.68 to -1.15) |
| **Thailand** | Both | 289.17(187.89 to 412.55) | 1.24(0.81 to 1.77) |  | 500.93(330.7 to 732.29) | 2.08(1.37 to 3.05) |  | 1.29(0.77 to 1.82) |
| **Timor-Leste** | Both | 1.69(0.86 to 3.05) | 0.62(0.32 to 1.11) |  | 4.65(2.78 to 7.39) | 1.11(0.67 to 1.75) |  | 1.93(1.29 to 2.57) |
| **Togo** | Both | 14.11(8.75 to 22.32) | 1.34(0.83 to 2.1) |  | 51.78(28.11 to 88.55) | 1.75(0.95 to 2.99) |  | 0.95(0.76 to 1.15) |
| **Tokelau** | Both | 0.02(0.01 to 0.03) | 3.07(1.7 to 4.85) |  | 0.02(0.01 to 0.03) | 4.74(3.01 to 6.97) |  | 0.58(0.23 to 0.94) |
| **Tonga** | Both | 0.85(0.54 to 1.26) | 3.2(2.07 to 4.71) |  | 1.19(0.73 to 1.8) | 3.55(2.19 to 5.37) |  | 0.31(0.2 to 0.42) |
| **Trinidad and Tobago** | Both | 9.11(7.85 to 10.61) | 1.96(1.69 to 2.28) |  | 13.84(9.53 to 19.15) | 2.27(1.56 to 3.13) |  | 0.02(-0.19 to 0.23) |
| **Tunisia** | Both | 22.32(14.96 to 32.28) | 0.81(0.54 to 1.17) |  | 50.18(30.52 to 77.99) | 0.98(0.59 to 1.52) |  | 0.58(0.4 to 0.77) |
| **Turkmenistan** | Both | 21.3(18.3 to 24.5) | 1.66(1.42 to 1.91) |  | 28.2(20.61 to 38.59) | 1.36(1 to 1.87) |  | -0.3(-0.65 to 0.04) |
| **Tuvalu** | Both | 0.09(0.05 to 0.16) | 2.73(1.43 to 4.55) |  | 0.13(0.07 to 0.21) | 2.77(1.58 to 4.52) |  | -0.06(-0.11 to -0.01) |
| **Türkiye** | Both | 90.11(55.61 to 136.53) | 0.44(0.27 to 0.66) |  | 429.27(286.85 to 603.71) | 1.26(0.84 to 1.77) |  | 5.62(4.55 to 6.7) |
| **Uganda** | Both | 61.41(34.84 to 99.37) | 1.33(0.76 to 2.14) |  | 309.73(186.53 to 483.31) | 2.44(1.49 to 3.76) |  | 0.93(0.52 to 1.34) |
| **Ukraine** | Both | 497.98(407.61 to 595.56) | 2.36(1.93 to 2.82) |  | 216.89(114.87 to 356.83) | 1.15(0.61 to 1.9) |  | -3(-3.3 to -2.71) |
| **United Arab Emirates** | Both | 7.29(4.39 to 11.47) | 0.64(0.38 to 1) |  | 41.18(25.46 to 62.79) | 0.64(0.38 to 1.02) |  | -0.49(-0.93 to -0.05) |
| **United Kingdom** | Both | 469.29(456.82 to 481.12) | 2.23(2.17 to 2.28) |  | 267.37(257.94 to 277.28) | 1.08(1.04 to 1.12) |  | -2.25(-2.5 to -2) |
| **United Republic of Tanzania** | Both | 145.68(91.65 to 219.53) | 1.91(1.21 to 2.87) |  | 468.25(265.9 to 777.27) | 2.4(1.37 to 3.95) |  | 0.7(0.62 to 0.78) |
| **United States Virgin Islands** | Both | 1.11(0.74 to 1.58) | 2.76(1.84 to 3.94) |  | 0.66(0.37 to 1.13) | 2.55(1.43 to 4.35) |  | -0.21(-0.56 to 0.15) |
| **United States of America** | Both | 2144.78(2080.28 to 2217.26) | 1.9(1.84 to 1.96) |  | 1290.7(1215.27 to 1363.75) | 1.07(1.01 to 1.14) |  | -1.93(-2.18 to -1.68) |
| **Uruguay** | Both | 25.17(20.42 to 30.62) | 2.26(1.84 to 2.75) |  | 23.49(18.75 to 28.86) | 1.9(1.51 to 2.33) |  | -1(-1.24 to -0.76) |
| **Uzbekistan** | Both | 114.69(91.12 to 140.91) | 1.58(1.25 to 1.94) |  | 206.74(154.5 to 271.02) | 1.39(1.04 to 1.82) |  | -0.49(-0.71 to -0.26) |
| **Vanuatu** | Both | 0.69(0.39 to 1.09) | 1.38(0.79 to 2.18) |  | 2.56(1.51 to 3.94) | 2.34(1.39 to 3.59) |  | 1.2(1.02 to 1.39) |
| **Venezuela (Bolivarian Republic of)** | Both | 104.52(90.23 to 121.31) | 1.48(1.28 to 1.72) |  | 210.2(146.4 to 292.34) | 2(1.4 to 2.78) |  | 0.72(0.26 to 1.19) |
| **Viet Nam** | Both | 228.21(135.95 to 352.21) | 0.97(0.58 to 1.49) |  | 466.15(296.92 to 698.8) | 1.05(0.67 to 1.57) |  | 0.21(0.09 to 0.34) |
| **Yemen** | Both | 13.95(6.82 to 24.7) | 0.37(0.18 to 0.66) |  | 82.45(46.01 to 137.83) | 0.67(0.37 to 1.11) |  | 2.06(1.81 to 2.31) |
| **Zambia** | Both | 49.1(27.33 to 78.56) | 2.23(1.25 to 3.56) |  | 287.61(117.79 to 560.88) | 4.25(1.77 to 8.19) |  | 2.28(1.8 to 2.76) |
| **Zimbabwe** | Both | 32.37(20.91 to 47.96) | 1.11(0.72 to 1.64) |  | 144.39(84.66 to 234.51) | 2.67(1.57 to 4.34) |  | 4.09(3.19 to 5) |

ASMR: age-standardized mortality rate; EAPC: estimated annual percentage change; CI: confidence interval; UI: uncertainty interval.

**Table S6.** The numbers of DALYs, YLDs and YLLs for breast cancer among adolescents and young adults at the national level in 1990 and 2021.

| **C****haracteristics** | **Sex** | **DALYs (95% UI)** | |  | **YLDs (95% UI)** | |  | **YLLs (95% UI)** | |
| --- | --- | --- | --- | --- | --- | --- | --- | --- | --- |
|  |  | **1990** | **2021** |  | **1990** | **2021** |  | **1990** | **2021** |
| **Afghanistan** | Both | 1411.56(514.2 to 2984.42) | 7508.39(2897.86 to 15546.19) |  | 35.17(10.27 to 84.1) | 241.9(74.49 to 547.14) |  | 1376.39(500.13 to 2911.61) | 7266.49(2813.21 to 15031.29) |
| **Albania** | Both | 648.76(473.34 to 866) | 436.28(297.06 to 612.96) |  | 23.35(12.44 to 37.84) | 30.29(15.92 to 50.45) |  | 625.41(457.93 to 836.71) | 405.99(275.79 to 571.46) |
| **Algeria** | Both | 3157.39(2003.74 to 4659.08) | 8167.76(5134.66 to 12359.42) |  | 132.95(70.16 to 231.83) | 589.12(282.4 to 1059.09) |  | 3024.44(1924.49 to 4437.2) | 7578.64(4754.16 to 11379.67) |
| **American Samoa** | Both | 24.17(15.37 to 36.78) | 38.37(24.84 to 56.57) |  | 0.8(0.4 to 1.38) | 1.29(0.67 to 2.15) |  | 23.37(14.83 to 35.49) | 37.08(23.92 to 54.71) |
| **Andorra** | Both | 24.1(14.87 to 36.93) | 20.14(11.47 to 31.62) |  | 1.84(0.88 to 3.25) | 2.36(1.14 to 4.26) |  | 22.26(13.81 to 34.24) | 17.78(10.07 to 27.88) |
| **Angola** | Both | 2159.63(1308.71 to 3465.56) | 10088.83(5754.57 to 15906.34) |  | 39.51(17.9 to 71.22) | 253.48(114.92 to 477.34) |  | 2120.12(1282.68 to 3407.96) | 9835.35(5626.98 to 15452.39) |
| **Antigua and Barbuda** | Both | 31.73(26.36 to 37.69) | 35.66(30.35 to 41.68) |  | 1.24(0.73 to 1.92) | 1.89(1.08 to 2.84) |  | 30.48(25.49 to 36.26) | 33.77(28.74 to 39.34) |
| **Argentina** | Both | 14945.58(12235.39 to 18149.67) | 16529.52(13316.58 to 20077.96) |  | 500.55(284.79 to 784.15) | 863.34(490.51 to 1352.41) |  | 14445.02(11860.44 to 17506) | 15666.18(12657.96 to 18944.62) |
| **Armenia** | Both | 2541.25(2378.55 to 2702.2) | 704.18(581.91 to 828.82) |  | 94.62(55.71 to 149.51) | 40.28(24.14 to 63.26) |  | 2446.63(2294 to 2590.97) | 663.9(546.82 to 783.52) |
| **Australia** | Both | 6643.66(5543.24 to 7884.25) | 5211.42(4221.07 to 6414.08) |  | 444.01(265.84 to 658.91) | 626.33(375.03 to 960.61) |  | 6199.65(5175.43 to 7329.65) | 4585.09(3711.5 to 5656.12) |
| **Austria** | Both | 2823.27(2312.35 to 3367.76) | 1748.65(1418.93 to 2127.09) |  | 194.37(110.93 to 304.2) | 197.1(111.96 to 314.8) |  | 2628.91(2169.86 to 3139.24) | 1551.54(1264.43 to 1874.09) |
| **Azerbaijan** | Both | 3442.16(2694.59 to 4130.19) | 3292.95(2374.17 to 4369.08) |  | 104.21(60.04 to 166.61) | 148.71(78.93 to 249.71) |  | 3337.96(2632.33 to 4021.33) | 3144.24(2259.66 to 4187.68) |
| **Bahamas** | Both | 233.17(195.8 to 276.64) | 389.42(282.53 to 532.04) |  | 8.16(4.86 to 12.8) | 17.08(9.94 to 27.44) |  | 225.01(189.14 to 266.23) | 372.33(270.75 to 508.4) |
| **Bahrain** | Both | 177.84(118.77 to 256.45) | 504(333.13 to 725.92) |  | 8.74(4.45 to 15.04) | 46.89(24.77 to 79.34) |  | 169.11(112.52 to 244.42) | 457.12(300.64 to 654.55) |
| **Bangladesh** | Both | 19123.71(10552.97 to 32204.39) | 50003.69(30233.35 to 77962.41) |  | 380.89(162.8 to 738.04) | 1772.82(847.16 to 3164.52) |  | 18742.83(10366.38 to 31587.47) | 48230.87(29121.65 to 75087.53) |
| **Barbados** | Both | 165.54(140.62 to 191.37) | 172.45(127.7 to 226.93) |  | 6.67(3.88 to 10.33) | 9.36(5.02 to 15.39) |  | 158.86(135.06 to 183.88) | 163.09(120.11 to 215.7) |
| **Belarus** | Both | 3947.93(3164.87 to 4849.3) | 2262.32(1529.81 to 3127.66) |  | 172.88(101.46 to 272.85) | 163.14(88.01 to 273.96) |  | 3775.05(3029.8 to 4640.68) | 2099.18(1426.74 to 2890.5) |
| **Belgium** | Both | 4973.8(4126.13 to 5904.26) | 2433.31(1920.58 to 2961.23) |  | 335.37(197.3 to 527.12) | 263.77(151.39 to 410.12) |  | 4638.43(3846.49 to 5528.62) | 2169.54(1739.21 to 2629.65) |
| **Belize** | Both | 23.07(19.04 to 27.38) | 109.88(89.82 to 135.96) |  | 0.76(0.42 to 1.24) | 4.59(2.8 to 7.21) |  | 22.32(18.51 to 26.46) | 105.3(86.36 to 129.16) |
| **Benin** | Both | 724.82(428.5 to 1137.03) | 2748.34(1502.82 to 4580.69) |  | 14.71(7.13 to 26.26) | 70.28(31.44 to 129.8) |  | 710.11(421.6 to 1111.85) | 2678.06(1467.96 to 4460.14) |
| **Bermuda** | Both | 36.01(28.77 to 45.16) | 17.07(12.63 to 22.14) |  | 1.81(1 to 2.9) | 1.6(0.89 to 2.57) |  | 34.21(27.27 to 42.86) | 15.47(11.35 to 19.9) |
| **Bhutan** | Both | 79.89(44.03 to 130.89) | 174.92(92.63 to 302.18) |  | 1.63(0.69 to 3.1) | 5.79(2.38 to 11.23) |  | 78.26(42.83 to 128.34) | 169.13(89.72 to 292.38) |
| **Bolivia (Plurinational State of)** | Both | 1625.37(856.06 to 2724.57) | 3564.46(2131.3 to 5459.05) |  | 33.43(14.62 to 63.82) | 115.2(54.87 to 205.86) |  | 1591.94(838.82 to 2670.8) | 3449.26(2067.48 to 5294.01) |
| **Bosnia and Herzegovina** | Both | 1153.55(865.65 to 1486.27) | 528.64(341.72 to 732.79) |  | 46.49(26.49 to 75.91) | 36.5(19.28 to 60.02) |  | 1107.06(830.56 to 1428.58) | 492.15(318.9 to 679.17) |
| **Botswana** | Both | 351.36(166.52 to 646.82) | 1064.06(518.91 to 1863.08) |  | 7.8(3.05 to 15.82) | 30.83(12.38 to 59.33) |  | 343.56(162.47 to 631.77) | 1033.24(503.94 to 1808.62) |
| **Brazil** | Both | 46882.16(43245.1 to 50723.81) | 94128.31(86933.6 to 102160.37) |  | 1378.51(944.72 to 1889.73) | 4163.17(2892.16 to 5653.68) |  | 45503.65(42014.31 to 49202.55) | 89965.14(83085.33 to 97386.54) |
| **Brunei Darussalam** | Both | 116.35(72.4 to 171.99) | 210.27(139.76 to 300.73) |  | 4.2(1.92 to 7.18) | 11.04(5.75 to 18.3) |  | 112.15(69.26 to 165.62) | 199.24(131.98 to 283.55) |
| **Bulgaria** | Both | 3744.5(3029.48 to 4533.48) | 2120.57(1624.66 to 2656.26) |  | 187.79(111.19 to 288.37) | 144.29(81.49 to 230.85) |  | 3556.7(2876.91 to 4311.35) | 1976.28(1526.41 to 2476.36) |
| **Burkina Faso** | Both | 3299.31(1929.1 to 5245.98) | 10248.9(5532.63 to 16776.92) |  | 66.96(32.2 to 120.15) | 247.61(110.82 to 450.82) |  | 3232.35(1890.98 to 5142.14) | 10001.3(5411.63 to 16414.81) |
| **Burundi** | Both | 1588.73(953.79 to 2608.57) | 4084.64(2480.6 to 6527.93) |  | 29.6(14.13 to 53.86) | 91.09(43.44 to 167.9) |  | 1559.13(938.02 to 2564.42) | 3993.54(2424.28 to 6377.22) |
| **Cabo Verde** | Both | 110.83(70.85 to 168.76) | 158(93.47 to 247.65) |  | 2.92(1.5 to 5.06) | 6.33(2.98 to 11.49) |  | 107.91(68.9 to 163.93) | 151.67(89.96 to 238.45) |
| **Cambodia** | Both | 2431.86(1196.79 to 4460.41) | 7473.21(4602.64 to 11638.66) |  | 50.73(19.92 to 102.4) | 236.15(108.14 to 405.92) |  | 2381.13(1172.42 to 4363.9) | 7237.06(4453.07 to 11256.1) |
| **Cameroon** | Both | 2367.79(1445.38 to 3699.15) | 10691.38(5938.7 to 17041.34) |  | 49.59(24.32 to 89.36) | 283.86(127.35 to 516.7) |  | 2318.2(1417.57 to 3624.06) | 10407.52(5793.63 to 16596.95) |
| **Canada** | Both | 11748.86(9779.49 to 13795.97) | 8548.39(6818.24 to 10552.31) |  | 927.24(559.42 to 1423.64) | 951.12(528.58 to 1501.69) |  | 10821.62(9036.4 to 12777.37) | 7597.26(6086.66 to 9403.23) |
| **Central African Republic** | Both | 583.13(338.5 to 927.76) | 1619.03(894.25 to 2602.36) |  | 10.28(4.77 to 18.64) | 30.69(12.97 to 56.28) |  | 572.85(333.16 to 909.9) | 1588.33(877.93 to 2549.7) |
| **Chad** | Both | 781.27(422.4 to 1300.6) | 2874.8(1656.37 to 4603.84) |  | 15.13(6.52 to 28.97) | 63.53(26.83 to 116.88) |  | 766.13(414.65 to 1275.45) | 2811.27(1616.97 to 4512.46) |
| **Chile** | Both | 3551.87(2820.89 to 4378.5) | 3911.42(3108.9 to 4874.15) |  | 128.23(73.75 to 203.49) | 282.44(161.73 to 450.2) |  | 3423.64(2730.55 to 4216.2) | 3628.98(2887.33 to 4492.74) |
| **China** | Both | 329424.94(256745.2 to 417078.3) | 297531.98(218407.09 to 391156.1) |  | 11072.63(7092.54 to 15979.09) | 24467.46(15079 to 36876.06) |  | 318352.3(248417.37 to 402773.37) | 273064.52(200786.06 to 358342.81) |
| **Colombia** | Both | 8679.04(6924.5 to 10581.43) | 16341.26(12068.82 to 21513.12) |  | 370.45(222.42 to 564.97) | 1325.33(725.08 to 2173.43) |  | 8308.59(6661.55 to 10165.83) | 15015.92(11083.89 to 19873) |
| **Comoros** | Both | 137.55(71.97 to 218.18) | 415.27(261.55 to 660.03) |  | 2.79(1.2 to 5.21) | 10.51(4.97 to 19.18) |  | 134.76(70.45 to 213.58) | 404.76(254.59 to 642.88) |
| **Congo** | Both | 735.99(358.63 to 1326.75) | 3071.49(1566.67 to 5345.33) |  | 14.45(5.52 to 28.39) | 81.2(33.01 to 160.16) |  | 721.53(351.97 to 1301.58) | 2990.29(1529.41 to 5205.87) |
| **Cook Islands** | Both | 14.06(8.23 to 21.95) | 11.99(7.38 to 18.32) |  | 0.54(0.25 to 0.97) | 0.65(0.32 to 1.14) |  | 13.52(7.96 to 21.08) | 11.34(6.95 to 17.28) |
| **Costa Rica** | Both | 680.57(545.12 to 847.07) | 1791.23(1373.56 to 2298.77) |  | 42.74(24.14 to 67.46) | 164.65(90.16 to 254.39) |  | 637.84(511.3 to 793.62) | 1626.58(1247.45 to 2077.17) |
| **Coted'Ivoire** | Both | 2489.06(1500.01 to 3844.44) | 9841.22(5605.5 to 15701.08) |  | 51.93(26 to 91.78) | 266.4(124.65 to 481.27) |  | 2437.13(1467.99 to 3774.28) | 9574.82(5419.91 to 15323.36) |
| **Croatia** | Both | 1692.9(1377.83 to 2060.72) | 775.07(593.89 to 972.81) |  | 104.23(59.82 to 161.69) | 77.28(42.45 to 124.11) |  | 1588.67(1297.72 to 1924.37) | 697.78(540.96 to 876.07) |
| **Cuba** | Both | 3653.53(2918.67 to 4494.26) | 2120.27(1584.53 to 2764.48) |  | 173.45(100.46 to 271.8) | 143.38(77.31 to 233.5) |  | 3480.08(2784.31 to 4290.01) | 1976.89(1470.31 to 2580.17) |
| **Cyprus** | Both | 223.34(152.46 to 320.39) | 299.31(206.68 to 423.2) |  | 13.19(6.5 to 22.84) | 37.17(19.49 to 64.24) |  | 210.15(144.62 to 301.24) | 262.15(180.79 to 369.89) |
| **Czechia** | Both | 3070.55(2514.75 to 3705.61) | 2166(1700.64 to 2705.74) |  | 162.59(90.36 to 251.91) | 206.01(121.34 to 325.26) |  | 2907.96(2379.13 to 3526.47) | 1959.99(1540.91 to 2448.35) |
| **Democratic People's Republic of Korea** | Both | 5197.52(2937.45 to 8509.4) | 7847.06(4563.77 to 12531.27) |  | 158.84(69.35 to 285.43) | 342.58(161.15 to 599.79) |  | 5038.68(2836.68 to 8257.88) | 7504.48(4366.6 to 11965.11) |
| **Democratic Republic of the Congo** | Both | 7338.02(4272.73 to 11780.62) | 23269.39(13915.34 to 36903.17) |  | 141.25(65.35 to 254.68) | 557.15(269.56 to 984.74) |  | 7196.78(4202.19 to 11547.01) | 22712.24(13597.58 to 36127.4) |
| **Denmark** | Both | 2405.05(1982.39 to 2855.19) | 1042.9(842.67 to 1283.73) |  | 142.91(84.45 to 219.09) | 113.14(65.42 to 177.5) |  | 2262.14(1874.79 to 2680.39) | 929.76(751.61 to 1129.16) |
| **Djibouti** | Both | 100.31(56.27 to 164.29) | 569.52(298.03 to 974.71) |  | 2.1(0.99 to 3.97) | 15.19(6.74 to 29.32) |  | 98.21(55.11 to 160.85) | 554.33(289.69 to 949.16) |
| **Dominica** | Both | 20.51(14.15 to 29.05) | 32.89(21.24 to 48.56) |  | 0.67(0.37 to 1.11) | 1.24(0.63 to 2.22) |  | 19.84(13.72 to 28.21) | 31.65(20.35 to 46.68) |
| **Dominican Republic** | Both | 1934.88(1343.97 to 2667.31) | 3530(2166.16 to 5270.1) |  | 54.42(29.16 to 90.38) | 137.95(68.08 to 235.67) |  | 1880.46(1307.45 to 2595.81) | 3392.04(2099.78 to 5063.43) |
| **Ecuador** | Both | 1899.46(1522.2 to 2362.19) | 4374.71(2995.8 to 6086.75) |  | 50.22(28.38 to 81.5) | 194.47(106.98 to 323.68) |  | 1849.24(1486.19 to 2304.03) | 4180.24(2856.08 to 5823.34) |
| **Egypt** | Both | 13115.6(9310.67 to 18017.99) | 36371.55(23909.33 to 51450) |  | 461.13(256.68 to 759.13) | 2160.1(1114.25 to 3654.99) |  | 12654.47(9024.87 to 17398.38) | 34211.46(22437.76 to 48309.15) |
| **El Salvador** | Both | 783.2(594.09 to 1010.65) | 1689.07(1158.92 to 2441.25) |  | 28.17(15.46 to 46.06) | 117.22(61.5 to 200.68) |  | 755.03(574.61 to 975.17) | 1571.86(1075.18 to 2268.45) |
| **Equatorial Guinea** | Both | 91.78(53.86 to 150.18) | 621.71(305.48 to 1127.09) |  | 1.67(0.76 to 3.09) | 20(8.05 to 39.94) |  | 90.1(53.15 to 147.55) | 601.7(294.63 to 1090.47) |
| **Eritrea** | Both | 910.45(550.89 to 1432.06) | 3140.58(1756.26 to 5069.1) |  | 17.08(8.1 to 31.35) | 70.13(33.68 to 124.15) |  | 893.37(539.64 to 1404.03) | 3070.45(1717.1 to 4958.55) |
| **Estonia** | Both | 509.09(412.75 to 626.43) | 189.48(142.03 to 245.23) |  | 24.34(14.06 to 38.61) | 16.43(9.04 to 26.36) |  | 484.75(390.71 to 598.53) | 173.05(129.23 to 222.93) |
| **Eswatini** | Both | 178.22(104.39 to 282.83) | 555.15(255.47 to 1001.38) |  | 4(1.9 to 7.46) | 14.41(5.44 to 28.89) |  | 174.22(101.98 to 276.34) | 540.74(248.45 to 980.25) |
| **Ethiopia** | Both | 17534.21(9857.26 to 28422.97) | 45887.52(33196.76 to 61874.31) |  | 305.54(141.71 to 563.63) | 1191.5(719.3 to 1841.92) |  | 17228.67(9665.74 to 27936.23) | 44696.02(32407.34 to 60293.55) |
| **Fiji** | Both | 603.17(386.75 to 916.23) | 792.28(489.39 to 1231.81) |  | 17.61(9.11 to 30.83) | 22.59(11.06 to 40.04) |  | 585.56(375.18 to 889.17) | 769.68(477.27 to 1194.01) |
| **Finland** | Both | 1856.75(1510.62 to 2249.94) | 907.97(721.48 to 1115.41) |  | 126.7(73.28 to 196.77) | 113.04(63.8 to 184.71) |  | 1730.05(1410.2 to 2094.54) | 794.94(636.2 to 968.95) |
| **France** | Both | 22596.71(18736.9 to 27074.24) | 14970.58(12114.31 to 18164.85) |  | 1515.32(886.5 to 2337.22) | 1966.81(1151.6 to 3044.74) |  | 21081.39(17452.37 to 25112.33) | 13003.77(10663.86 to 15660.68) |
| **Gabon** | Both | 289.02(173.45 to 443.62) | 785.09(439.79 to 1288.84) |  | 6.18(2.99 to 10.87) | 23.6(10.34 to 45.12) |  | 282.84(169.67 to 434.25) | 761.49(426.77 to 1252.48) |
| **Gambia** | Both | 74.33(41.7 to 120.73) | 357.59(209.72 to 587.47) |  | 1.65(0.76 to 3.05) | 9.77(4.29 to 18.33) |  | 72.67(40.77 to 118.09) | 347.82(203.79 to 567.52) |
| **Georgia** | Both | 3870.78(3438.13 to 4336.92) | 1272.3(1066.44 to 1475.33) |  | 155.27(90.95 to 236.82) | 61.77(37.35 to 95.63) |  | 3715.51(3299.28 to 4154.63) | 1210.53(1018.11 to 1402.64) |
| **Germany** | Both | 34435.39(28691.18 to 41111) | 19483.24(15984.23 to 23377.07) |  | 2011.58(1184.32 to 3028.74) | 2066.57(1189.47 to 3174.86) |  | 32423.8(27022.26 to 38613.9) | 17416.66(14384.43 to 20911.21) |
| **Ghana** | Both | 5634.63(3446.76 to 8801.29) | 16231.5(9421.81 to 25568.06) |  | 121.83(58.06 to 214.68) | 454.71(211.27 to 838.95) |  | 5512.8(3370.49 to 8604.7) | 15776.79(9158.48 to 24968.39) |
| **Greece** | Both | 3991.45(3476.16 to 4545.53) | 2077.99(1789.03 to 2393.56) |  | 298.79(173.42 to 457.26) | 208.78(120.97 to 314.46) |  | 3692.65(3243.77 to 4162.73) | 1869.21(1621.24 to 2146.81) |
| **Greenland** | Both | 31.47(21.31 to 44.45) | 13.87(7.94 to 21.66) |  | 1.05(0.52 to 1.74) | 0.7(0.32 to 1.29) |  | 30.42(20.57 to 42.95) | 13.17(7.53 to 20.35) |
| **Grenada** | Both | 48.1(37.18 to 61.02) | 54.21(39.44 to 71.86) |  | 1.45(0.8 to 2.36) | 2.32(1.24 to 3.79) |  | 46.65(36.18 to 59.35) | 51.89(37.79 to 68.43) |
| **Guam** | Both | 41.96(29.88 to 58.08) | 61.45(45.59 to 81.45) |  | 1.94(1.07 to 3.09) | 2.72(1.56 to 4.51) |  | 40.02(28.59 to 55.52) | 58.74(43.74 to 77.66) |
| **Guatemala** | Both | 1027.74(869.58 to 1210.3) | 3186.38(2506.17 to 3984.62) |  | 29.62(17.19 to 45.68) | 154.81(90.77 to 240.35) |  | 998.12(846.52 to 1175.59) | 3031.57(2385.64 to 3769.13) |
| **Guinea** | Both | 1003.1(612.7 to 1562.28) | 3599.18(1937.3 to 6093.47) |  | 19.35(9.14 to 34.03) | 83.4(36.99 to 161.03) |  | 983.75(602.14 to 1532.53) | 3515.79(1889.45 to 5941.92) |
| **Guinea-Bissau** | Both | 295.03(174.14 to 466.52) | 921.99(543.69 to 1453.76) |  | 5.31(2.64 to 9.37) | 20.4(9.74 to 37.51) |  | 289.72(170.51 to 459.21) | 901.59(530.17 to 1420.69) |
| **Guyana** | Both | 263.93(191.09 to 347.47) | 400.06(254.3 to 589.91) |  | 6.43(3.76 to 10.18) | 12.36(6.24 to 20.33) |  | 257.51(186.58 to 339.08) | 387.71(245.07 to 570.45) |
| **Haiti** | Both | 2721.58(1215.82 to 5100.47) | 7102.8(3582.12 to 12218.03) |  | 51.24(19.64 to 104.89) | 161.58(62.8 to 320.65) |  | 2670.34(1194.44 to 4998.68) | 6941.22(3506.82 to 11901.05) |
| **Honduras** | Both | 605.41(388.91 to 908.72) | 1687.08(869.4 to 2896.56) |  | 18.75(9.09 to 31.81) | 73.12(29.41 to 140.8) |  | 586.67(375.09 to 878.49) | 1613.96(836.21 to 2772.8) |
| **Hungary** | Both | 4826.03(3905.24 to 5859.69) | 2083.45(1634.09 to 2595) |  | 216.88(123.14 to 336.79) | 166.67(94.06 to 261.22) |  | 4609.15(3726.87 to 5573.9) | 1916.78(1506.04 to 2374.36) |
| **Iceland** | Both | 96.99(78.17 to 118.94) | 69.66(54.92 to 87.28) |  | 7.46(4.43 to 11.53) | 8.24(4.69 to 13.39) |  | 89.53(72.59 to 109.08) | 61.42(48.75 to 75.79) |
| **India** | Both | 179774.35(149044.54 to 214333.73) | 479274.14(388590.11 to 586582.35) |  | 3894.42(2606.51 to 5451.99) | 15607.16(10039.85 to 22489.99) |  | 175879.93(145829.95 to 209898.2) | 463666.98(375982.66 to 567173.69) |
| **Indonesia** | Both | 54503.75(36659.08 to 79562.45) | 113037.68(80710.13 to 159440.19) |  | 1316.93(725.42 to 2146.83) | 3805.11(2342.04 to 5780.72) |  | 53186.81(35879.71 to 77663.43) | 109232.57(77778.11 to 154850.78) |
| **Iran (Islamic Republic of)** | Both | 9273.52(7578.75 to 11387.12) | 30212(26185.92 to 34975.45) |  | 545.21(345.6 to 804.35) | 3233.73(2139.06 to 4659.87) |  | 8728.31(7137.48 to 10701.38) | 26978.26(23445.37 to 31354.38) |
| **Iraq** | Both | 3571.59(2290.34 to 5330.92) | 12827.19(8140.12 to 19692.54) |  | 147.72(74.04 to 261.7) | 887.61(451.49 to 1553.15) |  | 3423.86(2193.99 to 5113.61) | 11939.58(7506.2 to 18338.96) |
| **Ireland** | Both | 1530.34(1263.19 to 1824.58) | 973.48(778.84 to 1189.76) |  | 97.41(58.45 to 150.07) | 117.51(67.57 to 182.28) |  | 1432.93(1177.85 to 1707.6) | 855.96(683.91 to 1038.23) |
| **Israel** | Both | 2442.14(2044.95 to 2869.23) | 1986.47(1652.84 to 2371.44) |  | 136.41(80.58 to 203.32) | 196.52(109.56 to 318.39) |  | 2305.73(1925.11 to 2705.69) | 1789.95(1491.59 to 2123.86) |
| **Italy** | Both | 24702.78(23155.52 to 26254.23) | 9884.3(9045.43 to 10754.42) |  | 1865.02(1243.03 to 2664.13) | 1229.23(828.2 to 1742.63) |  | 22837.76(21578.63 to 24091.69) | 8655.07(8046.07 to 9315.35) |
| **Jamaica** | Both | 627.47(498.32 to 776.74) | 1615.7(1057.53 to 2345.69) |  | 24.31(14.66 to 37.34) | 78.1(38.95 to 135.74) |  | 603.16(477.87 to 746.49) | 1537.6(1004.47 to 2227.09) |
| **Japan** | Both | 26643.77(25456.49 to 27981.68) | 17110.71(16096.54 to 18309.09) |  | 2286.48(1529.53 to 3176.94) | 2223.2(1481.63 to 3156.77) |  | 24357.29(23473.1 to 25222.9) | 14887.52(14313.77 to 15492.32) |
| **Jordan** | Both | 703.26(435.75 to 1084.28) | 2942.41(1681.8 to 4723.9) |  | 33.33(15.31 to 59.38) | 253.91(114.61 to 456.97) |  | 669.93(416.34 to 1029.23) | 2688.5(1538.04 to 4337.75) |
| **Kazakhstan** | Both | 8450.35(7458.65 to 9602.25) | 4318.97(3676.39 to 4980.39) |  | 286.57(169.9 to 432.49) | 227.1(134.26 to 354.9) |  | 8163.78(7221.73 to 9262.8) | 4091.87(3485.02 to 4700.26) |
| **Kenya** | Both | 3832.4(2516.04 to 5647.21) | 19582.07(11721.19 to 30741.67) |  | 89.81(52.14 to 144.21) | 559.35(291.98 to 955.43) |  | 3742.58(2455.21 to 5513.59) | 19022.72(11409.42 to 29830.65) |
| **Kiribati** | Both | 46.07(29.31 to 69.16) | 107.32(62.26 to 174.32) |  | 0.94(0.47 to 1.65) | 2.31(1.1 to 4.08) |  | 45.13(28.64 to 67.75) | 105.01(60.94 to 170.59) |
| **Kuwait** | Both | 275.37(211.34 to 349.7) | 1450.23(1102.39 to 1863.56) |  | 21.68(12.29 to 35.19) | 173.88(96.35 to 281.01) |  | 253.68(195.55 to 320.06) | 1276.34(987.01 to 1616.92) |
| **Kyrgyzstan** | Both | 1661.19(1279.95 to 2064.08) | 1698.64(1262.65 to 2192.39) |  | 52.23(31.54 to 81.36) | 77.7(41.37 to 126.42) |  | 1608.96(1238.22 to 2003.8) | 1620.95(1206.43 to 2088.85) |
| **Lao People's Democratic Republic** | Both | 897.66(405.95 to 1676.39) | 2990.67(1772.28 to 4857.36) |  | 17.3(6.09 to 35.99) | 83.48(39.95 to 153.01) |  | 880.35(397.51 to 1644.73) | 2907.19(1727.65 to 4724.51) |
| **Latvia** | Both | 901.26(724.79 to 1115.62) | 324.68(239.88 to 420.08) |  | 35.07(20.05 to 55.36) | 18.19(10.45 to 30.23) |  | 866.19(697.79 to 1073.1) | 306.49(226.57 to 394.09) |
| **Lebanon** | Both | 805.28(453.34 to 1303.45) | 1871.41(1221.28 to 2684.47) |  | 41.13(18.31 to 76.93) | 182.84(95.41 to 326.19) |  | 764.16(431.11 to 1237.41) | 1688.57(1104.48 to 2390.57) |
| **Lesotho** | Both | 247.14(116.16 to 463.83) | 769.42(407.31 to 1277.52) |  | 5.27(1.94 to 10.75) | 17.79(8.08 to 33.38) |  | 241.87(113.66 to 454.04) | 751.62(397.16 to 1251.31) |
| **Liberia** | Both | 391.68(224.91 to 639.71) | 1596.61(870.02 to 2754.6) |  | 7.69(3.87 to 13.52) | 43.22(18.5 to 81.69) |  | 383.99(219.86 to 626.62) | 1553.4(844.91 to 2685.01) |
| **Libya** | Both | 480.54(294.57 to 731.23) | 2001.85(1190.75 to 3219.61) |  | 23.06(11.16 to 38.26) | 133.86(60.06 to 249.63) |  | 457.47(279.03 to 694.96) | 1867.99(1115.06 to 2977.67) |
| **Lithuania** | Both | 1200.43(958.9 to 1466.77) | 471.18(341.81 to 607.77) |  | 60.67(34.67 to 97.68) | 32.51(17.02 to 52.69) |  | 1139.76(912.16 to 1386.51) | 438.66(319.73 to 565.22) |
| **Luxembourg** | Both | 200.94(173.09 to 232.19) | 117.34(97.09 to 138.26) |  | 12.18(7.19 to 18.58) | 13.32(7.71 to 20.86) |  | 188.76(162.94 to 217.52) | 104.02(87.41 to 122) |
| **Madagascar** | Both | 3678.54(2199.48 to 5756.3) | 12077.33(7305.35 to 18913.34) |  | 72.62(34.39 to 129.94) | 290.58(144.58 to 508.62) |  | 3605.92(2158.52 to 5644.57) | 11786.75(7120.43 to 18473.16) |
| **Malawi** | Both | 1941.89(1210.05 to 2897.42) | 7690.86(4381.65 to 12715.15) |  | 38.03(19.16 to 66.21) | 190.02(89.31 to 358.98) |  | 1903.86(1188.21 to 2843.98) | 7500.85(4280.29 to 12409.2) |
| **Malaysia** | Both | 6510.64(4346.81 to 9075.43) | 15512.64(10525.38 to 21891.98) |  | 194.26(102.77 to 322.66) | 736.47(374.06 to 1249.76) |  | 6316.38(4215.72 to 8819.33) | 14776.17(10057.47 to 20757.11) |
| **Maldives** | Both | 24.31(8.51 to 51.53) | 82.16(49.95 to 126.9) |  | 0.62(0.16 to 1.47) | 4.54(2.28 to 8.15) |  | 23.69(8.24 to 50.26) | 77.62(47.37 to 120.15) |
| **Mali** | Both | 1771.91(1160.06 to 2683.89) | 5297.64(2935.96 to 8888.67) |  | 35.06(17.71 to 61.41) | 129.39(57.95 to 238.91) |  | 1736.85(1135.35 to 2634.45) | 5168.25(2869.16 to 8658.77) |
| **Malta** | Both | 169.78(138.66 to 204) | 104.69(83.69 to 128.34) |  | 9.78(5.82 to 15.32) | 10.27(5.79 to 16.5) |  | 160(130.31 to 192.61) | 94.42(75.7 to 114.97) |
| **Marshall Islands** | Both | 19.18(10.83 to 31.17) | 46.22(22.54 to 82.68) |  | 0.45(0.2 to 0.82) | 1.15(0.47 to 2.26) |  | 18.73(10.6 to 30.45) | 45.06(21.85 to 80.26) |
| **Mauritania** | Both | 309.29(167.56 to 498.74) | 1025.8(615.07 to 1644.95) |  | 6.38(2.61 to 11.84) | 32.84(15.74 to 60.35) |  | 302.91(164.23 to 488.78) | 992.96(592.66 to 1589.01) |
| **Mauritius** | Both | 291.14(247.8 to 341.2) | 575.73(477.96 to 675.48) |  | 10.21(5.88 to 15.47) | 27.85(16.85 to 41.71) |  | 280.93(240.16 to 328.04) | 547.88(455.55 to 640.76) |
| **Mexico** | Both | 22018.08(21003.04 to 23115.51) | 37749.34(30868.26 to 45130.98) |  | 893.06(642.35 to 1201.65) | 2451.64(1641.55 to 3545.08) |  | 21125.02(20166.9 to 22195.05) | 35297.7(28898.23 to 42216.56) |
| **Micronesia (Federated States of)** | Both | 51.31(28.37 to 83.31) | 69.17(39 to 109.3) |  | 1.19(0.55 to 2.21) | 1.87(0.87 to 3.37) |  | 50.12(27.58 to 81.53) | 67.3(37.95 to 105.88) |
| **Monaco** | Both | 15.9(10.8 to 22.25) | 18.48(11.01 to 28.67) |  | 1.26(0.65 to 2.09) | 2.07(1.07 to 3.73) |  | 14.64(9.96 to 20.41) | 16.41(9.77 to 25.27) |
| **Mongolia** | Both | 219.48(143.89 to 321.7) | 478.78(314.93 to 689.41) |  | 5.2(2.6 to 8.8) | 17.7(8.98 to 30.26) |  | 214.28(140.52 to 313.2) | 461.08(302.13 to 664.12) |
| **Montenegro** | Both | 255.26(192.5 to 341.44) | 170.75(123.23 to 229.35) |  | 15.83(9.09 to 25.37) | 13.85(7.67 to 22.16) |  | 239.43(180.56 to 321.91) | 156.9(112.78 to 210.9) |
| **Morocco** | Both | 3242.83(1877.7 to 5246.23) | 9395.34(5488.98 to 16164.14) |  | 113.65(52.27 to 205.58) | 511.16(241.7 to 980.79) |  | 3129.18(1816.65 to 5066.84) | 8884.18(5157.83 to 15249.69) |
| **Mozambique** | Both | 4204.84(2573.04 to 6352.21) | 14520.56(8056.18 to 24393.69) |  | 79.01(37.5 to 139.1) | 327.79(151.73 to 618.5) |  | 4125.83(2531.4 to 6221.26) | 14192.77(7849.48 to 23867.76) |
| **Myanmar** | Both | 26613.11(13875.83 to 44857.74) | 33249.24(20865.73 to 49896.22) |  | 540.5(212.51 to 1026.67) | 1040.64(526.19 to 1881.56) |  | 26072.6(13578.28 to 43937.81) | 32208.6(20194.46 to 48209.56) |
| **Namibia** | Both | 356.86(225.08 to 540.7) | 1193.26(670.95 to 1998.13) |  | 7.85(3.79 to 14.12) | 35.79(16.08 to 68.92) |  | 349.01(220.06 to 528.03) | 1157.46(652.77 to 1937.77) |
| **Nauru** | Both | 7.26(3.33 to 13.25) | 10.24(4.68 to 19.09) |  | 0.19(0.07 to 0.37) | 0.28(0.1 to 0.58) |  | 7.08(3.25 to 12.91) | 9.96(4.56 to 18.59) |
| **Nepal** | Both | 2829.15(1617.5 to 4542.19) | 7480.16(4227.78 to 12515.82) |  | 56.43(25.32 to 105.38) | 231.3(100.85 to 435.47) |  | 2772.73(1585.7 to 4451.99) | 7248.85(4111.96 to 12166.54) |
| **Netherlands** | Both | 7658.72(6412.74 to 8941.91) | 3810.39(3079.42 to 4626.99) |  | 503.28(301.04 to 775.81) | 448.85(271.42 to 718.56) |  | 7155.44(5991.62 to 8347.23) | 3361.53(2751.75 to 4054.25) |
| **New Zealand** | Both | 1881.69(1579.27 to 2191.27) | 1350.13(1136.75 to 1576.52) |  | 112.97(69.9 to 169.85) | 128.5(82.56 to 186.14) |  | 1768.72(1488.82 to 2063.42) | 1221.63(1027.56 to 1426.26) |
| **Nicaragua** | Both | 543.23(381.64 to 747.4) | 1293.97(838.06 to 1922.24) |  | 21.24(11 to 35.58) | 83.2(43.7 to 143.27) |  | 521.99(365.77 to 719.17) | 1210.77(785.14 to 1786.44) |
| **Niger** | Both | 926.36(517.41 to 1558.21) | 2727.36(1428.82 to 4775.31) |  | 17.61(8.58 to 33.7) | 63.08(25.46 to 128.67) |  | 908.75(509.28 to 1527.59) | 2664.28(1401.41 to 4663.61) |
| **Nigeria** | Both | 19459.31(13358.65 to 27966.96) | 75979.25(42585.12 to 125818.89) |  | 402.92(231.09 to 657.15) | 2094.08(1019.66 to 3607.85) |  | 19056.39(13096.67 to 27412.02) | 73885.17(41463.23 to 122480.43) |
| **Niue** | Both | 1.06(0.64 to 1.69) | 1.46(0.94 to 2.15) |  | 0.03(0.02 to 0.06) | 0.05(0.03 to 0.09) |  | 1.03(0.62 to 1.63) | 1.41(0.91 to 2.06) |
| **North Macedonia** | Both | 917.86(708.97 to 1163.95) | 606.97(438.78 to 834.32) |  | 35.51(20.15 to 56.14) | 39.41(21.28 to 65.24) |  | 882.35(682.12 to 1115.22) | 567.57(410.3 to 778.06) |
| **Northern Mariana Islands** | Both | 32.6(18.3 to 51.81) | 22.49(14.69 to 32.09) |  | 1.41(0.61 to 2.51) | 0.99(0.5 to 1.65) |  | 31.19(17.52 to 49.63) | 21.51(14.02 to 30.59) |
| **Norway** | Both | 1429.33(1323.54 to 1537.96) | 691.77(625.2 to 766.09) |  | 87.95(60.03 to 125.64) | 78.31(51.32 to 114.36) |  | 1341.38(1246.81 to 1441.65) | 613.46(558.84 to 673.28) |
| **Oman** | Both | 73.24(42.9 to 115.66) | 270.57(172.26 to 417.85) |  | 3.59(1.61 to 6.59) | 24.77(12.6 to 43.82) |  | 69.65(40.64 to 110.18) | 245.8(156.77 to 379.55) |
| **Pakistan** | Both | 36388.97(24273.19 to 52204.05) | 181641.04(114367.12 to 272163.23) |  | 742.86(415.43 to 1209.4) | 4744.58(2547.49 to 7892.9) |  | 35646.11(23808.62 to 51204.38) | 176896.46(111359.14 to 264962.74) |
| **Palau** | Both | 15.54(8.76 to 25.07) | 13.55(8.29 to 20.74) |  | 0.51(0.22 to 0.93) | 0.5(0.24 to 0.87) |  | 15.04(8.46 to 24.31) | 13.06(8.05 to 19.95) |
| **Palestine** | Both | 534.18(300.72 to 877.25) | 1904.8(1319.34 to 2654.09) |  | 23.67(10.72 to 42.31) | 133.18(70.5 to 226.14) |  | 510.5(287.83 to 837.49) | 1771.62(1237.41 to 2446.87) |
| **Panama** | Both | 488.92(386.15 to 618.78) | 1487.66(1060.23 to 2021.15) |  | 26.37(15.93 to 40.55) | 128.96(71.47 to 209.52) |  | 462.55(366.24 to 582.98) | 1358.7(973.1 to 1841.38) |
| **Papua New Guinea** | Both | 1724.2(902.42 to 2802.92) | 6015.6(3464.15 to 9518.36) |  | 42.63(18.5 to 77.46) | 144.71(64.76 to 265.15) |  | 1681.57(883.1 to 2739.18) | 5870.88(3375.48 to 9308.31) |
| **Paraguay** | Both | 958.52(641.42 to 1362.3) | 2315.46(1487.01 to 3585.12) |  | 29.62(15.6 to 48.44) | 98.88(47.15 to 174.05) |  | 928.9(618.12 to 1321.11) | 2216.58(1413.6 to 3434.52) |
| **Peru** | Both | 5267.75(3740.68 to 7308.96) | 8057.94(5015.81 to 12246.32) |  | 138.04(76.18 to 225.42) | 431.09(219.95 to 735.18) |  | 5129.71(3640.14 to 7117.26) | 7626.85(4745.07 to 11581.19) |
| **Philippines** | Both | 24104.64(20757.92 to 27700.44) | 59060.5(44676.47 to 76875.82) |  | 651.73(429.92 to 906.91) | 1962.95(1216.4 to 3004.45) |  | 23452.91(20185.86 to 26901.57) | 57097.56(43227.38 to 74007.33) |
| **Poland** | Both | 14688.23(13850.99 to 15526.97) | 8998.08(7661.2 to 10360.72) |  | 592.35(402.86 to 833.41) | 678.54(426.82 to 980.94) |  | 14095.88(13332.24 to 14871.59) | 8319.54(7059.14 to 9532.58) |
| **Portugal** | Both | 4472.67(3633.32 to 5426.68) | 2439.72(1962.45 to 2962.35) |  | 260.52(154.23 to 394.25) | 279.23(161.82 to 439.91) |  | 4212.16(3440.62 to 5118.2) | 2160.49(1744.42 to 2603.63) |
| **Puerto Rico** | Both | 1493.65(1189.8 to 1827.67) | 714.97(523.93 to 949.3) |  | 72.03(40.66 to 113.14) | 56.59(30.9 to 92.23) |  | 1421.62(1135.37 to 1734.23) | 658.39(482.62 to 876.32) |
| **Qatar** | Both | 156.37(97.6 to 234.13) | 853.15(524.43 to 1347.97) |  | 8.72(4.1 to 14.81) | 99.4(46.66 to 183.2) |  | 147.64(92.59 to 219.54) | 753.74(463.97 to 1186.78) |
| **Republic of Korea** | Both | 10345.56(7635.37 to 13351.06) | 8911.41(6529.84 to 11977.33) |  | 464.15(253.36 to 772.21) | 1078.75(571.57 to 1773.84) |  | 9881.4(7337.72 to 12756.01) | 7832.66(5738.09 to 10542.16) |
| **Republic of Moldova** | Both | 2003.35(1726.28 to 2330.8) | 906.99(742.37 to 1107.29) |  | 73.7(43.58 to 114.64) | 51.83(30.66 to 81.66) |  | 1929.65(1654.41 to 2238.4) | 855.17(699.65 to 1043.45) |
| **Romania** | Both | 9499.8(7607.02 to 11554.92) | 4263.31(3297.98 to 5432.15) |  | 343.1(203.89 to 538.27) | 281.09(159.57 to 444.2) |  | 9156.7(7381.94 to 11154.56) | 3982.22(3069.97 to 5117.12) |
| **Russian Federation** | Both | 58957.63(56340.89 to 61709.77) | 43291.77(37771.9 to 48573.27) |  | 2334.74(1623.92 to 3212.4) | 2633.49(1836.18 to 3597.19) |  | 56622.9(54316.89 to 59050.08) | 40658.27(35381.94 to 45613.45) |
| **Rwanda** | Both | 3372.13(1889.64 to 5555.95) | 6478.37(3710.92 to 10768.68) |  | 60.66(27.74 to 113.17) | 167.59(81.26 to 305.32) |  | 3311.47(1858.64 to 5435.95) | 6310.79(3612.69 to 10510.65) |
| **Saint Kitts and Nevis** | Both | 41.1(34.35 to 48.54) | 18.69(12.7 to 25.3) |  | 1.12(0.68 to 1.72) | 0.82(0.46 to 1.35) |  | 39.99(33.33 to 47.15) | 17.86(12.22 to 23.99) |
| **Saint Lucia** | Both | 61.9(52.48 to 72.72) | 80.07(61.73 to 101.72) |  | 1.94(1.19 to 2.91) | 3.6(2.15 to 5.82) |  | 59.97(50.79 to 70.52) | 76.46(59.33 to 96.73) |
| **Saint Vincent and the Grenadines** | Both | 56.14(47.02 to 66.5) | 62.81(49.8 to 77.48) |  | 1.81(1.08 to 2.73) | 2.5(1.43 to 3.94) |  | 54.33(45.55 to 64.38) | 60.31(47.76 to 74.11) |
| **Samoa** | Both | 32.63(19.93 to 50.5) | 63.35(35.69 to 98.84) |  | 0.97(0.49 to 1.7) | 2.07(0.92 to 3.92) |  | 31.66(19.35 to 48.87) | 61.29(34.67 to 95.62) |
| **San Marino** | Both | 6.44(4.27 to 9.18) | 3.92(2 to 6.75) |  | 0.54(0.28 to 0.92) | 0.45(0.18 to 0.87) |  | 5.9(3.92 to 8.42) | 3.47(1.77 to 6.03) |
| **Sao Tome and Principe** | Both | 16.67(8.85 to 26.9) | 66.7(35.81 to 115.78) |  | 0.38(0.17 to 0.7) | 2.18(0.99 to 4.24) |  | 16.29(8.66 to 26.37) | 64.52(34.64 to 111.81) |
| **Saudi Arabia** | Both | 2207.07(1320.22 to 3377.31) | 12178.61(7320.89 to 18738.02) |  | 68.64(33.37 to 118.86) | 744.72(357.07 to 1328.09) |  | 2138.43(1273.4 to 3265.71) | 11433.89(6887.25 to 17642.3) |
| **Senegal** | Both | 1308.38(777.05 to 2031.54) | 4408.73(2490.69 to 7486.65) |  | 27.28(12.98 to 49.92) | 119.68(53.26 to 225.58) |  | 1281.11(761.5 to 1987.19) | 4289.05(2415.5 to 7281.27) |
| **Serbia** | Both | 4033.06(2900.6 to 5590) | 2239.63(1564.81 to 3056.62) |  | 170.05(91.13 to 281.02) | 166.47(88.39 to 275.46) |  | 3863.01(2785.92 to 5336.23) | 2073.16(1439.77 to 2835.08) |
| **Seychelles** | Both | 26.29(17.68 to 37.5) | 53.41(35.63 to 74.76) |  | 0.82(0.43 to 1.4) | 2.39(1.31 to 3.96) |  | 25.47(17.07 to 36.22) | 51.01(34.32 to 71.23) |
| **Sierra Leone** | Both | 651.5(338.74 to 1120.79) | 2443.14(1296.21 to 4081.01) |  | 13.2(5.27 to 25.84) | 61.33(26.72 to 120.13) |  | 638.3(330.19 to 1098.59) | 2381.81(1260.72 to 3961.93) |
| **Singapore** | Both | 1306.02(1061 to 1577.58) | 1043.76(846.66 to 1254.81) |  | 73.64(42.1 to 116.7) | 123.55(67.14 to 197.96) |  | 1232.38(1008.78 to 1488.43) | 920.21(751.31 to 1111.58) |
| **Slovakia** | Both | 1837.35(1364.21 to 2406.08) | 1169.82(787.75 to 1631.5) |  | 76.91(41.6 to 122.55) | 81.64(41.95 to 136.84) |  | 1760.43(1294.93 to 2303.53) | 1088.18(734.33 to 1515.29) |
| **Slovenia** | Both | 594.68(481.2 to 726.24) | 280.28(209.57 to 369.83) |  | 35.73(19.34 to 56.28) | 30.26(17.02 to 49.94) |  | 558.95(453.07 to 683.9) | 250.02(186.53 to 323.71) |
| **Solomon Islands** | Both | 69.37(29.98 to 120.95) | 348.54(193.5 to 556.98) |  | 1.61(0.61 to 3.31) | 8.42(3.61 to 15.46) |  | 67.76(29.27 to 118.38) | 340.11(189.37 to 543.12) |
| **Somalia** | Both | 1701.88(979.73 to 2884.17) | 4519.09(2538.92 to 7556.6) |  | 30.7(14.12 to 56.69) | 88.82(39.41 to 158.71) |  | 1671.18(961.68 to 2831.62) | 4430.27(2482.85 to 7425.53) |
| **South Africa** | Both | 18747.45(15354.33 to 22536.16) | 28390.7(21681.66 to 35974.16) |  | 487.58(325.35 to 707.68) | 929.37(593.25 to 1365.43) |  | 18259.86(14964.44 to 21977.52) | 27461.34(20955.06 to 34809.02) |
| **South Sudan** | Both | 1115.72(625.75 to 1833.02) | 2506.75(1439.07 to 4033.75) |  | 22.2(10.07 to 41.07) | 59.58(26.7 to 105.15) |  | 1093.52(614.81 to 1797.56) | 2447.17(1407.65 to 3935.53) |
| **Spain** | Both | 16915.12(14031.49 to 20042.85) | 8135.92(6704.02 to 9772.3) |  | 1110.86(643.96 to 1727.76) | 926.18(537.82 to 1429.9) |  | 15804.25(13175.05 to 18629.2) | 7209.75(5937.74 to 8630.48) |
| **Sri Lanka** | Both | 4194.06(2725.57 to 6248.65) | 4759.46(2762.72 to 7471.61) |  | 130.81(67.03 to 223.72) | 275.22(125.14 to 494.69) |  | 4063.25(2629.61 to 6028.99) | 4484.23(2596.69 to 7081.53) |
| **Sudan** | Both | 2228.74(1036.41 to 4228.7) | 9373.91(4194.84 to 16422.36) |  | 67.37(24.63 to 144.78) | 445.81(171.82 to 894.78) |  | 2161.37(1000.77 to 4095.07) | 8928.1(3995.66 to 15592.19) |
| **Suriname** | Both | 116.04(75.14 to 164.07) | 225.52(141.47 to 332.41) |  | 3.25(1.7 to 5.32) | 7.94(3.99 to 13.66) |  | 112.78(73.13 to 159.43) | 217.58(135.3 to 321.09) |
| **Sweden** | Both | 2614.78(2204.13 to 3059.11) | 1370.85(1048.43 to 1736.3) |  | 189.22(120.56 to 282.2) | 156.37(92.07 to 251.73) |  | 2425.56(2035.5 to 2832.61) | 1214.47(935.28 to 1534.21) |
| **Switzerland** | Both | 2034.07(1645.96 to 2465.33) | 1223.76(971.43 to 1511.09) |  | 155.8(89.51 to 243.6) | 151.47(87.65 to 233.88) |  | 1878.27(1522.53 to 2256.94) | 1072.29(860.5 to 1315.17) |
| **Syrian Arab Republic** | Both | 2403.38(1455.8 to 3729) | 3632.32(2342.05 to 5499.93) |  | 103.03(50.54 to 181.93) | 274.66(134.66 to 493.89) |  | 2300.35(1395.61 to 3580.98) | 3357.66(2174.01 to 5067.8) |
| **Taiwan (Province of China)** | Both | 7145.56(5795.29 to 8509.41) | 5592.35(4472.9 to 6922.59) |  | 364.54(209.92 to 582.31) | 512.49(286.08 to 819.2) |  | 6781.02(5532.07 to 8062.14) | 5079.86(4066.66 to 6264.64) |
| **Tajikistan** | Both | 1598.92(1134.34 to 2144.04) | 2807.12(1441.44 to 4811.48) |  | 44.98(24.93 to 72.39) | 95.13(40.03 to 187.8) |  | 1553.94(1101.61 to 2083.37) | 2711.99(1391.03 to 4643.38) |
| **Thailand** | Both | 16674.87(10773.7 to 23878.46) | 29412.37(19414.15 to 43058.83) |  | 556.11(297.79 to 944.24) | 1735.45(888.82 to 2934.27) |  | 16118.77(10448.23 to 23070.51) | 27676.92(18230.85 to 40502.52) |
| **Timor-Leste** | Both | 96.92(49.25 to 175.56) | 271.07(161.23 to 430.58) |  | 2.1(0.78 to 4.18) | 7.89(3.44 to 14.86) |  | 94.82(48.15 to 171.76) | 263.18(156.99 to 419.15) |
| **Togo** | Both | 811.83(501.29 to 1290.52) | 2977.7(1610.39 to 5096.25) |  | 17.21(8.44 to 31.78) | 79.36(36.76 to 151.29) |  | 794.62(491.49 to 1262.33) | 2898.34(1570.81 to 4961.41) |
| **Tokelau** | Both | 0.91(0.5 to 1.44) | 1.32(0.84 to 1.96) |  | 0.02(0.01 to 0.05) | 0.05(0.02 to 0.08) |  | 0.89(0.49 to 1.4) | 1.28(0.81 to 1.89) |
| **Tonga** | Both | 49.3(31.49 to 73.56) | 69.01(42.26 to 105.02) |  | 1.56(0.78 to 2.71) | 2.36(1.16 to 4.14) |  | 47.74(30.42 to 71.07) | 66.66(40.9 to 101.24) |
| **Trinidad and Tobago** | Both | 525.49(452.3 to 612.32) | 795.79(546.27 to 1104.33) |  | 16.92(10.4 to 25.8) | 35.49(18.55 to 57.23) |  | 508.57(438.17 to 592.45) | 760.3(523.08 to 1051.86) |
| **Tunisia** | Both | 1305.69(873.6 to 1888.51) | 2994.45(1806.27 to 4620.21) |  | 66.39(34.74 to 114.81) | 248.06(123.78 to 446) |  | 1239.3(829.15 to 1794.35) | 2746.39(1667.73 to 4274.11) |
| **Turkmenistan** | Both | 1233.99(1063.94 to 1421.05) | 1638.89(1196.06 to 2239.45) |  | 36.61(21.61 to 58.18) | 65.54(36.75 to 110.3) |  | 1197.38(1028.87 to 1377.48) | 1573.35(1150.24 to 2151.89) |
| **Tuvalu** | Both | 5.41(2.83 to 9.06) | 7.28(4.11 to 11.91) |  | 0.13(0.05 to 0.24) | 0.21(0.1 to 0.38) |  | 5.29(2.76 to 8.84) | 7.07(3.99 to 11.57) |
| **Türkiye** | Both | 5290.38(3262.99 to 8021.17) | 26052.81(17346.67 to 36685.06) |  | 215.71(100.89 to 381.21) | 2253.34(1113.9 to 3824.53) |  | 5074.66(3125.66 to 7690.35) | 23799.47(15865.79 to 33544.8) |
| **Uganda** | Both | 3535.91(2001.42 to 5758.07) | 17946.87(10707.44 to 28199.21) |  | 74.76(34.91 to 139.36) | 462.42(212.03 to 844.11) |  | 3461.15(1958.46 to 5618.7) | 17484.45(10461.98 to 27427.14) |
| **Ukraine** | Both | 28516.16(23433.81 to 34371.94) | 12468.39(6544.49 to 20442) |  | 1099.18(654.74 to 1724.56) | 587.05(257.5 to 1087.64) |  | 27416.97(22445.56 to 32796.06) | 11881.34(6285.64 to 19556.58) |
| **United Arab Emirates** | Both | 430.51(259.3 to 679.6) | 2406.51(1470.74 to 3654.23) |  | 19.02(9.27 to 33.51) | 166.97(79.68 to 300.15) |  | 411.49(246.72 to 648.99) | 2239.53(1382.46 to 3422.93) |
| **United Kingdom** | Both | 27578.16(26703.1 to 28519.56) | 16389.85(15647.17 to 17307.62) |  | 1742.98(1223.16 to 2397.98) | 1659.15(1142.97 to 2295.5) |  | 25835.18(25147.57 to 26489.65) | 14730.71(14210.45 to 15276.15) |
| **United Republic of Tanzania** | Both | 8546.63(5367.65 to 12937.14) | 27666.76(15591.58 to 46045.84) |  | 175.82(91.3 to 314.46) | 730.3(346.91 to 1352.45) |  | 8370.81(5250.55 to 12650.47) | 26936.47(15199.23 to 44921.39) |
| **United States Virgin Islands** | Both | 63.54(42.31 to 90.64) | 38.43(21.64 to 65.99) |  | 2.48(1.25 to 4.24) | 1.98(0.93 to 3.81) |  | 61.05(40.61 to 87.35) | 36.45(20.5 to 62.21) |
| **United States of America** | Both | 128388.12(123398.55 to 134116.46) | 79973.23(74540.87 to 85502.21) |  | 10162(7136.36 to 13847.99) | 8691.1(6118.59 to 11969.33) |  | 118226.12(114633.05 to 122243.98) | 71282.13(67105.76 to 75321.74) |
| **Uruguay** | Both | 1448.85(1166.88 to 1766.5) | 1385.19(1104.33 to 1702.19) |  | 55.2(31.77 to 85.31) | 81.51(47.41 to 131.26) |  | 1393.65(1129.53 to 1697.2) | 1303.69(1039.01 to 1603.14) |
| **Uzbekistan** | Both | 6640.22(5277.75 to 8156.64) | 12013.4(8933.58 to 15784.84) |  | 209.99(126.46 to 326.77) | 485.28(277.13 to 763.05) |  | 6430.24(5110.13 to 7897.24) | 11528.12(8603.01 to 15121.36) |
| **Vanuatu** | Both | 40.17(22.88 to 63.77) | 147.87(87.31 to 228.6) |  | 1.01(0.48 to 1.89) | 3.64(1.81 to 6.36) |  | 39.16(22.27 to 62.22) | 144.23(84.9 to 222.88) |
| **Venezuela (Bolivarian Republic of)** | Both | 6100.51(5257.53 to 7112.83) | 12464.11(8636.12 to 17210.89) |  | 258.63(153.15 to 399.5) | 787.44(424.99 to 1290.83) |  | 5841.87(5042.15 to 6775.9) | 11676.68(8136.04 to 16223.99) |
| **Viet Nam** | Both | 13123.38(7775.33 to 20290.17) | 27056.8(17099.67 to 40869.97) |  | 375.38(173.04 to 670.91) | 1385.43(688.35 to 2448.47) |  | 12748.01(7555.16 to 19750.14) | 25671.37(16329.71 to 38541.62) |
| **Yemen** | Both | 794.74(386.2 to 1417.8) | 4759(2644.5 to 7984.94) |  | 24.41(9.08 to 48.59) | 194.95(88.04 to 375.47) |  | 770.32(375.19 to 1365.96) | 4564.05(2537.66 to 7647.9) |
| **Zambia** | Both | 2889.76(1601.63 to 4623.15) | 16966.42(6866.93 to 33350.99) |  | 55.88(25.05 to 102.15) | 424.45(148.87 to 911.06) |  | 2833.88(1571.81 to 4539.56) | 16541.97(6690.76 to 32494.79) |
| **Zimbabwe** | Both | 1852.79(1197.35 to 2746.2) | 8228.94(4835.54 to 13361.84) |  | 44.99(23.15 to 76.13) | 192.72(89.46 to 362.86) |  | 1807.8(1165.74 to 2685.38) | 8036.22(4711.36 to 13041.4) |

DALYs: disability-adjusted life years; YLDs: years lived with disability; YLLs: years of life lost; UI: uncertainty interval.

**Table S7.** Age-standardized DALYs, YLDs, and YLLs rates for breast cancer among adolescents and young adults at the national level in 1990 and 2021, as well as time trends.

| **Characteristics** | **Sex** | **Age-standardized**  **DALYs rates  (per 100,000)**  **(95% CI)** | |  | **EAPC**  **(95% CI)** |  | **Age-standardized**  **YLDs rates  (per 100,000)**  **(95% CI)** | |  | **EAPC**  **(95% CI)** |  | **Age-standardized**  **YLLs rates   (per 100,000)**  **(95% CI)** | |  | **EAPC**  **(95% CI)** |  |
| --- | --- | --- | --- | --- | --- | --- | --- | --- | --- | --- | --- | --- | --- | --- | --- | --- |
|  |  |  |  |  |  |  |  |  |  |  |  |  |  |  |  |  |
|  |  | **1990** | **2021** |  | **1990-2021** |  | **1990** | **2021** |  | **1990-2021** |  | **1990** | **2021** |  | **1990-2021** |  |
| **Afghanistan** | Both | 70.25(25.34 to 149.26) | 87.98(33.73 to 181.92) |  | 0.74(0.53 to 0.95) |  | 1.76(0.51 to 4.26) | 2.82(0.89 to 6.39) |  | 1.58(1.24 to 1.91) |  | 68.49(24.6 to 145.53) | 85.15(32.73 to 175.91) |  | 0.72(0.51 to 0.93) |  |
| **Albania** | Both | 51.25(37.57 to 68.08) | 45.58(31.08 to 63.94) |  | -0.13(-0.48 to 0.22) |  | 1.84(0.99 to 2.96) | 3.16(1.67 to 5.25) |  | 2.26(1.71 to 2.81) |  | 49.41(36.34 to 65.79) | 42.42(28.85 to 59.62) |  | -0.26(-0.6 to 0.08) |  |
| **Algeria** | Both | 41.13(26.2 to 60.59) | 41.82(26.24 to 63.36) |  | -0.12(-0.21 to -0.03) |  | 1.72(0.91 to 3) | 3.02(1.44 to 5.44) |  | 1.56(1.42 to 1.7) |  | 39.41(25.17 to 57.73) | 38.79(24.3 to 58.33) |  | -0.22(-0.3 to -0.13) |  |
| **American Samoa** | Both | 138.48(88.67 to 209.56) | 234.17(151.74 to 344.77) |  | 1.96(1.85 to 2.07) |  | 4.59(2.33 to 7.87) | 7.89(4.1 to 13.16) |  | 1.74(1.6 to 1.88) |  | 133.89(85.55 to 202.2) | 226.28(146.09 to 333.34) |  | 1.97(1.85 to 2.08) |  |
| **Andorra** | Both | 87.07(53.84 to 133.23) | 63.26(35.87 to 99.64) |  | -0.64(-0.84 to -0.44) |  | 6.62(3.18 to 11.71) | 7.48(3.59 to 13.53) |  | 0.82(0.48 to 1.16) |  | 80.45(50 to 123.57) | 55.78(31.53 to 87.7) |  | -0.8  (-0.99 to -0.6) |  |
| **Angola** | Both | 65.93(40.2 to 105.56) | 101.22(58.08 to 159) |  | 1.71(1.5 to 1.92) |  | 1.21(0.55 to 2.17) | 2.54(1.16 to 4.77) |  | 2.74(2.51 to 2.98) |  | 64.72(39.39 to 103.81) | 98.67(56.78 to 154.42) |  | 1.69(1.48 to 1.9) |  |
| **Antigua and Barbuda** | Both | 133.69(111.04 to 158.8) | 97.12(82.65 to 113.54) |  | -0.23(-0.54 to 0.08) |  | 5.22(3.04 to 8.05) | 5.15(2.93 to 7.76) |  | 0.73(0.44 to 1.03) |  | 128.47(107.41 to 152.76) | 91.96(78.27 to 107.15) |  | -0.28(-0.58 to 0.03) |  |
| **Argentina** | Both | 126(103.16 to 153) | 91.02(73.3 to 110.6) |  | -0.92(-1.14 to -0.7) |  | 4.22(2.4 to 6.61) | 4.76(2.7 to 7.45) |  | 0.45(0.25 to 0.65) |  | 121.78(100 to 147.58) | 86.26(69.67 to 104.36) |  | -0.98(-1.2 to -0.76) |  |
| **Armenia** | Both | 177.27(166.15 to 188.28) | 52.84(43.7 to 62.19) |  | -3.87(-4.29 to -3.45) |  | 6.58(3.89 to 10.4) | 3.03(1.81 to 4.77) |  | -2.36(-2.75 to -1.97) |  | 170.68(160.27 to 180.56) | 49.81(41.05 to 58.77) |  | -3.94(-4.36 to -3.52) |  |
| **Australia** | Both | 93.16(77.72 to 110.57) | 51.95(42.02 to 63.99) |  | -2.15(-2.29 to -2) |  | 6.23(3.73 to 9.25) | 6.27(3.75 to 9.62) |  | -0.04(-0.21 to 0.13) |  | 86.92(72.55 to 102.78) | 45.68(36.94 to 56.4) |  | -2.36(-2.52 to -2.21) |  |
| **Austria** | Both | 94.89(77.76 to 113.08) | 53.56(43.42 to 65.21) |  | -1.53(-1.87 to -1.2) |  | 6.51(3.71 to 10.18) | 6.05(3.43 to 9.66) |  | 0.09(-0.12 to 0.31) |  | 88.38(72.98 to 105.47) | 47.51(38.68 to 57.44) |  | -1.7(-2.04 to -1.35) |  |
| **Azerbaijan** | Both | 123.84(96.92 to 148.49) | 66.74(48.2 to 88.45) |  | -2.15(-2.33 to -1.98) |  | 3.73(2.15 to 5.95) | 3.01(1.6 to 5.06) |  | -0.72(-0.98 to -0.46) |  | 120.12(94.75 to 144.61) | 63.72(45.88 to 84.79) |  | -2.21(-2.38 to -2.03) |  |
| **Bahamas** | Both | 223.96(188.29 to 265.33) | 249.1(180.73 to 340.34) |  | -0.27(-0.52 to -0.01) |  | 7.79(4.66 to 12.21) | 10.93(6.36 to 17.56) |  | 0.65(0.44 to 0.86) |  | 216.17(181.91 to 255.53) | 238.17(173.19 to 325.21) |  | -0.3(-0.56 to -0.05) |  |
| **Bahrain** | Both | 63.63(42.5 to 91.55) | 62.1(40.95 to 89.75) |  | -0.54(-0.99 to -0.09) |  | 3.1(1.58 to 5.35) | 5.78(3.05 to 9.82) |  | 1.63(1.16 to 2.1) |  | 60.53(40.28 to 87.3) | 56.33(36.95 to 80.92) |  | -0.7(-1.15 to -0.25) |  |
| **Bangladesh** | Both | 52.36(29.17 to 87.62) | 74.03(44.78 to 115.24) |  | 1.01(0.85 to 1.17) |  | 1.05(0.45 to 2.02) | 2.62(1.25 to 4.68) |  | 2.96(2.78 to 3.15) |  | 51.32(28.67 to 85.93) | 71.41(43.14 to 111) |  | 0.95(0.8 to 1.11) |  |
| **Barbados** | Both | 151.87(129 to 175.52) | 159.74(118.23 to 210.37) |  | -0.13(-0.34 to 0.08) |  | 6.11(3.55 to 9.45) | 8.69(4.66 to 14.31) |  | 0.82(0.59 to 1.05) |  | 145.75(123.91 to 168.67) | 151.05(111.2 to 199.93) |  | -0.17(-0.38 to 0.04) |  |
| **Belarus** | Both | 92.07(73.81 to 113.1) | 57.09(38.53 to 79.09) |  | -2.35(-2.61 to -2.1) |  | 4.03(2.36 to 6.36) | 4.14(2.23 to 6.98) |  | -0.54(-0.79 to -0.29) |  | 88.04(70.65 to 108.23) | 52.94(35.92 to 73.03) |  | -2.46(-2.72 to -2.2) |  |
| **Belgium** | Both | 123.72(102.6 to 146.93) | 61.36(48.4 to 74.78) |  | -2.44(-2.63 to -2.25) |  | 8.34(4.9 to 13.13) | 6.67(3.83 to 10.38) |  | -0.86(-1.1 to -0.62) |  | 115.38(95.65 to 137.57) | 54.69(43.81 to 66.37) |  | -2.6(-2.78 to -2.41) |  |
| **Belize** | Both | 40.54(33.43 to 48.09) | 64.5(52.72 to 79.83) |  | 1.71(1.22 to 2.19) |  | 1.32(0.74 to 2.17) | 2.69(1.64 to 4.23) |  | 2.57(2.17 to 2.98) |  | 39.22(32.5 to 46.48) | 61.81(50.69 to 75.82) |  | 1.68(1.19 to 2.16) |  |
| **Benin** | Both | 51.52(30.64 to 80.61) | 65.09(35.77 to 107.59) |  | 0.5(0.34 to 0.67) |  | 1.05(0.51 to 1.87) | 1.66(0.75 to 3.04) |  | 1.26(1.11 to 1.41) |  | 50.47(30.15 to 78.81) | 63.44(34.94 to 104.76) |  | 0.49(0.33 to 0.65) |  |
| **Bermuda** | Both | 123.2(98.35 to 154.54) | 78.74(58.1 to 102.57) |  | -1.74(-1.99 to -1.49) |  | 6.18(3.41 to 9.92) | 7.45(4.15 to 11.99) |  | 0.32(0.16 to 0.48) |  | 117.02(93.23 to 146.66) | 71.28(52.19 to 92.1) |  | -1.9(-2.16 to -1.64) |  |
| **Bhutan** | Both | 39.63(22.02 to 64.62) | 49.85(26.43 to 85.99) |  | 0.47(0.34 to 0.59) |  | 0.81(0.34 to 1.54) | 1.65(0.68 to 3.19) |  | 2.15(2.05 to 2.26) |  | 38.82(21.42 to 63.38) | 48.2(25.6 to 83.2) |  | 0.42(0.3 to 0.55) |  |
| **Bolivia (Plurinational State of)** | Both | 77(40.7 to 128.89) | 75.32(45.11 to 115.16) |  | -0.36(-0.47 to -0.25) |  | 1.59(0.7 to 3.02) | 2.43(1.16 to 4.34) |  | 1.13(1.02 to 1.24) |  | 75.42(39.88 to 126.33) | 72.89(43.77 to 111.67) |  | -0.4(-0.51 to -0.29) |  |
| **Bosnia and Herzegovina** | Both | 59.67(44.78 to 76.88) | 44.88(29.03 to 62.25) |  | -0.88(-1.13 to -0.63) |  | 2.4(1.37 to 3.92) | 3.11(1.63 to 5.13) |  | 1.19(0.83 to 1.54) |  | 57.27(42.97 to 73.89) | 41.77(27.08 to 57.68) |  | -1(-1.25 to -0.74) |  |
| **Botswana** | Both | 88.19(41.83 to 161.92) | 94.18(45.92 to 165.08) |  | 0.73(0.27 to 1.2) |  | 1.96(0.77 to 3.96) | 2.73(1.1 to 5.26) |  | 1.54(1.02 to 2.07) |  | 86.23(40.8 to 158.13) | 91.45(44.59 to 160.25) |  | 0.71(0.25 to 1.18) |  |
| **Brazil** | Both | 83.95(77.44 to 90.82) | 100.52(92.8 to 109.13) |  | 0.39(0.27 to 0.5) |  | 2.46(1.69 to 3.37) | 4.46(3.1 to 6.06) |  | 1.71(1.57 to 1.84) |  | 81.49(75.24 to 88.1) | 96.07(88.69 to 104.03) |  | 0.34(0.22 to 0.45) |  |
| **Brunei Darussalam** | Both | 97.23(60.51 to 143.59) | 90.77(60.31 to 129.89) |  | 0.46(0.22 to 0.71) |  | 3.5(1.59 to 5.99) | 4.77(2.49 to 7.91) |  | 1.72(1.51 to 1.93) |  | 93.73(57.88 to 138.25) | 86.01(56.95 to 122.46) |  | 0.4(0.16 to 0.65) |  |
| **Bulgaria** | Both | 115.49(93.38 to 139.97) | 86.45(66.06 to 108.48) |  | -0.58(-0.78 to -0.37) |  | 5.81(3.44 to 8.92) | 5.92(3.34 to 9.47) |  | 0.4(0.12 to 0.69) |  | 109.69(88.67 to 133.04) | 80.53(62.03 to 101.07) |  | -0.64(-0.84 to -0.43) |  |
| **Burkina Faso** | Both | 127.77(75.17 to 202) | 138.6(75.13 to 225.97) |  | 0.32(0.12 to 0.52) |  | 2.6(1.26 to 4.65) | 3.35(1.51 to 6.08) |  | 0.9(0.74 to 1.05) |  | 125.17(73.68 to 197.95) | 135.24(73.48 to 221.08) |  | 0.3(0.1 to 0.5) |  |
| **Burundi** | Both | 90.52(54.47 to 148.19) | 88.06(53.62 to 140.5) |  | -0.4(-0.59 to -0.21) |  | 1.69(0.81 to 3.07) | 1.97(0.94 to 3.62) |  | 0.19(-0.01 to 0.38) |  | 88.83(53.56 to 145.69) | 86.09(52.4 to 137.25) |  | -0.41(-0.6 to -0.22) |  |
| **Cabo Verde** | Both | 119.87(77.04 to 180.74) | 60.58(35.87 to 94.92) |  | -1.89(-2.2 to -1.59) |  | 3.15(1.64 to 5.39) | 2.43(1.14 to 4.4) |  | -0.38(-0.76 to 0.01) |  | 116.73(74.91 to 175.59) | 58.15(34.53 to 91.41) |  | -1.94(-2.25 to -1.64) |  |
| **Cambodia** | Both | 78.03(38.67 to 142.73) | 102.94(63.48 to 160.12) |  | 0.92(0.87 to 0.96) |  | 1.63(0.65 to 3.28) | 3.25(1.49 to 5.57) |  | 2.41(2.31 to 2.5) |  | 76.4(37.89 to 139.62) | 99.69(61.43 to 154.87) |  | 0.88(0.83 to 0.92) |  |
| **Cameroon** | Both | 77.95(47.81 to 121.26) | 97.28(54.44 to 154.24) |  | 0.58(0.5 to 0.66) |  | 1.63(0.8 to 2.93) | 2.58(1.17 to 4.67) |  | 1.33(1.19 to 1.47) |  | 76.32(46.87 to 118.81) | 94.7(53.12 to 150.21) |  | 0.56(0.48 to 0.64) |  |
| **Canada** | Both | 94.09(78.3 to 110.52) | 62.99(50.2 to 77.82) |  | -1.27(-1.5 to -1.04) |  | 7.43(4.48 to 11.41) | 7.03(3.9 to 11.12) |  | -0.07(-0.22 to 0.07) |  | 86.66(72.35 to 102.35) | 55.96(44.79 to 69.31) |  | -1.39(-1.63 to -1.16) |  |
| **Central African Republic** | Both | 69.88(40.52 to 111.14) | 89.86(49.78 to 144.29) |  | 0.72(0.54 to 0.89) |  | 1.23(0.57 to 2.24) | 1.71(0.72 to 3.13) |  | 0.98(0.81 to 1.15) |  | 68.64(39.88 to 108.98) | 88.15(48.88 to 141.34) |  | 0.71(0.54 to 0.89) |  |
| **Chad** | Both | 46.2(25.01 to 76.67) | 61.1(35.46 to 97.33) |  | 1.1(1 to 1.19) |  | 0.9(0.38 to 1.72) | 1.35(0.57 to 2.48) |  | 1.53(1.4 to 1.66) |  | 45.31(24.55 to 75.19) | 59.75(34.61 to 95.41) |  | 1.09(0.99 to 1.18) |  |
| **Chile** | Both | 68.1(54.16 to 83.91) | 51.43(40.87 to 64.1) |  | -0.62(-0.94 to -0.29) |  | 2.45(1.42 to 3.87) | 3.71(2.13 to 5.91) |  | 1.74(1.49 to 1.98) |  | 65.65(52.43 to 80.82) | 47.72(37.95 to 59.09) |  | -0.75(-1.07 to -0.42) |  |
| **China** | Both | 65.65(51.15 to 83.15) | 52.1(38.32 to 68.46) |  | -0.99(-1.15 to -0.84) |  | 2.21(1.41 to 3.19) | 4.3(2.65 to 6.49) |  | 2.23(2.09 to 2.38) |  | 63.44(49.49 to 80.29) | 47.8(35.23 to 62.68) |  | -1.18(-1.33 to -1.03) |  |
| **Colombia** | Both | 71.38(56.99 to 87) | 81.41(60.12 to 107.16) |  | 0.63(0.26 to 1) |  | 3.03(1.83 to 4.62) | 6.59(3.61 to 10.8) |  | 2.72(2.43 to 3.02) |  | 68.35(54.84 to 83.59) | 74.82(55.22 to 99.01) |  | 0.5(0.13 to 0.87) |  |
| **Comoros** | Both | 104.74(55.85 to 164.99) | 145.54(91.95 to 230.8) |  | 0.48(-0.04 to 1) |  | 2.13(0.93 to 3.98) | 3.68(1.74 to 6.7) |  | 1.27(0.81 to 1.74) |  | 102.61(54.68 to 161.48) | 141.86(89.49 to 224.82) |  | 0.46(-0.06 to 0.98) |  |
| **Congo** | Both | 102.4(49.96 to 184.7) | 149.14(76.11 to 259.54) |  | 1.16(0.96 to 1.36) |  | 2.01(0.77 to 3.96) | 3.94(1.6 to 7.78) |  | 2.15(1.92 to 2.37) |  | 100.39(49.04 to 181.25) | 145.2(74.3 to 252.77) |  | 1.14(0.94 to 1.34) |  |
| **Cook Islands** | Both | 209.03(123.04 to 325.13) | 213.86(131.73 to 326.67) |  | 0.43(0.14 to 0.72) |  | 8.06(3.67 to 14.4) | 11.64(5.74 to 20.23) |  | 1.3(1.06 to 1.54) |  | 200.97(118.9 to 312.31) | 202.21(124.09 to 308.19) |  | 0.38(0.09 to 0.68) |  |
| **Costa Rica** | Both | 60.16(48.22 to 74.81) | 87.68(67.22 to 112.56) |  | 1.6(1.18 to 2.02) |  | 3.76(2.11 to 5.94) | 8.07(4.42 to 12.47) |  | 2.88(2.46 to 3.29) |  | 56.41(45.25 to 70.14) | 79.62(61.05 to 101.7) |  | 1.49(1.08 to 1.91) |  |
| **Coted'Ivoire** | Both | 65.92(39.89 to 101.14) | 97.32(55.54 to 154.95) |  | 1.42(1.28 to 1.55) |  | 1.37(0.69 to 2.41) | 2.63(1.23 to 4.74) |  | 2.24(2.11 to 2.38) |  | 64.55(39.04 to 99.29) | 94.69(53.7 to 151.24) |  | 1.4(1.26 to 1.53) |  |
| **Croatia** | Both | 82.39(67.02 to 100.38) | 51.78(39.57 to 65.14) |  | -1.21(-1.55 to -0.87) |  | 5.09(2.92 to 7.9) | 5.22(2.86 to 8.4) |  | 0.49(0.13 to 0.85) |  | 77.3(63.1 to 93.71) | 46.57(35.99 to 58.58) |  | -1.35(-1.69 to -1.02) |  |
| **Cuba** | Both | 88.29(70.57 to 108.56) | 53.55(40.01 to 69.82) |  | -1.52(-1.72 to -1.31) |  | 4.16(2.41 to 6.51) | 3.63(1.95 to 5.91) |  | -0.4(-0.59 to -0.2) |  | 84.13(67.35 to 103.67) | 49.92(37.12 to 65.15) |  | -1.58(-1.79 to -1.38) |  |
| **Cyprus** | Both | 71.94(49.21 to 103.04) | 45.26(31.05 to 64.4) |  | -1.57(-2.02 to -1.12) |  | 4.24(2.1 to 7.34) | 5.66(2.96 to 9.82) |  | 1.17(0.58 to 1.76) |  | 67.69(46.69 to 96.88) | 39.6(27.15 to 56.23) |  | -1.84(-2.29 to -1.4) |  |
| **Czechia** | Both | 74.21(60.84 to 89.49) | 58.27(45.68 to 73.02) |  | -0.75(-1.2 to -0.3) |  | 3.95(2.19 to 6.13) | 5.59(3.28 to 8.84) |  | 1.06(0.77 to 1.35) |  | 70.26(57.54 to 85.08) | 52.68(41.36 to 65.99) |  | -0.9(-1.36 to -0.44) |  |
| **Democratic People's Republic of Korea** | Both | 69.11(39.09 to 112.9) | 71.58(41.63 to 114.37) |  | 0.12(0.05 to 0.18) |  | 2.1(0.93 to 3.76) | 3.13(1.47 to 5.48) |  | 1.43(1.29 to 1.57) |  | 67.01(37.76 to 109.61) | 68.45(39.83 to 109.2) |  | 0.07(0.01 to 0.13) |  |
| **Democratic Republic of the Congo** | Both | 64.1(37.47 to 102.62) | 78.26(47.09 to 123.81) |  | 0.53(0.18 to 0.89) |  | 1.23(0.57 to 2.22) | 1.87(0.91 to 3.3) |  | 1.24(0.81 to 1.66) |  | 62.86(36.85 to 100.58) | 76.39(46.02 to 121.23) |  | 0.52(0.16 to 0.87) |  |
| **Denmark** | Both | 119.66(98.61 to 142.05) | 55.45(44.82 to 68.2) |  | -2.43(-2.74 to -2.12) |  | 7.12(4.21 to 10.91) | 6(3.47 to 9.4) |  | -0.21(-0.44 to 0.02) |  | 112.54(93.25 to 133.35) | 49.45(39.99 to 60.01) |  | -2.63(-2.94 to -2.32) |  |
| **Djibouti** | Both | 75.16(42.5 to 122.28) | 104.21(54.51 to 178.36) |  | 1.05(0.81 to 1.29) |  | 1.57(0.75 to 2.97) | 2.78(1.23 to 5.37) |  | 1.85(1.68 to 2.03) |  | 73.59(41.64 to 119.72) | 101.43(52.99 to 173.67) |  | 1.03(0.79 to 1.27) |  |
| **Dominica** | Both | 86.02(59.53 to 121.37) | 125.75(81.18 to 185.7) |  | 1.83(1.49 to 2.17) |  | 2.81(1.55 to 4.61) | 4.75(2.42 to 8.48) |  | 2.21(1.89 to 2.52) |  | 83.21(57.72 to 117.88) | 121(77.81 to 178.51) |  | 1.82(1.47 to 2.16) |  |
| **Dominican Republic** | Both | 77.99(54.4 to 106.93) | 79(48.53 to 117.88) |  | 0.4(0.22 to 0.58) |  | 2.19(1.18 to 3.62) | 3.08(1.52 to 5.26) |  | 1.38(1.13 to 1.62) |  | 75.8(52.92 to 104.07) | 75.92(47.04 to 113.27) |  | 0.36(0.18 to 0.55) |  |
| **Ecuador** | Both | 54.8(43.96 to 68.14) | 61.99(42.45 to 86.17) |  | 0.73(0.46 to 1.01) |  | 1.45(0.82 to 2.35) | 2.75(1.52 to 4.58) |  | 2.39(2.03 to 2.74) |  | 53.35(42.92 to 66.47) | 59.24(40.47 to 82.46) |  | 0.67(0.4 to 0.95) |  |
| **Egypt** | Both | 70.21(50.01 to 96.18) | 88.21(57.99 to 124.75) |  | 0.74(0.64 to 0.84) |  | 2.46(1.38 to 4.05) | 5.24(2.7 to 8.87) |  | 2.24(2.11 to 2.36) |  | 67.74(48.49 to 92.87) | 82.97(54.42 to 117.13) |  | 0.67(0.57 to 0.77) |  |
| **El Salvador** | Both | 47.53(36.14 to 61.17) | 72.29(49.71 to 104.41) |  | 1.36(1.17 to 1.54) |  | 1.7(0.94 to 2.78) | 4.99(2.63 to 8.53) |  | 3.52(3.13 to 3.92) |  | 45.83(34.98 to 59.04) | 67.3(46.12 to 97.08) |  | 1.24(1.06 to 1.42) |  |
| **Equatorial Guinea** | Both | 74.65(43.91 to 122.06) | 110.49(54.96 to 199.36) |  | 1.12(0.88 to 1.36) |  | 1.36(0.62 to 2.52) | 3.54(1.43 to 7.03) |  | 3.28(3 to 3.56) |  | 73.29(43.34 to 119.91) | 106.95(53.01 to 192.97) |  | 1.06(0.82 to 1.31) |  |
| **Eritrea** | Both | 90.55(54.81 to 142.46) | 127.05(71.19 to 204.83) |  | 1.05(0.95 to 1.15) |  | 1.7(0.8 to 3.14) | 2.84(1.37 to 5.02) |  | 1.63(1.52 to 1.73) |  | 88.84(53.69 to 139.65) | 124.21(69.6 to 200.4) |  | 1.04(0.94 to 1.14) |  |
| **Estonia** | Both | 81.3(65.89 to 100.07) | 38.11(28.52 to 49.41) |  | -2.76(-3.15 to -2.37) |  | 3.89(2.25 to 6.18) | 3.32(1.82 to 5.36) |  | -0.52(-0.89 to -0.15) |  | 77.41(62.37 to 95.61) | 34.79(25.94 to 44.88) |  | -2.92(-3.31 to -2.53) |  |
| **Eswatini** | Both | 79.19(46.41 to 125.47) | 120.48(55.62 to 217.09) |  | 1.59(1.07 to 2.12) |  | 1.78(0.85 to 3.32) | 3.12(1.18 to 6.23) |  | 1.97(1.53 to 2.41) |  | 77.41(45.34 to 122.57) | 117.36(54.1 to 212.57) |  | 1.58(1.06 to 2.11) |  |
| **Ethiopia** | Both | 115.27(64.85 to 186.84) | 120.19(87.68 to 161.01) |  | -0.14(-0.36 to 0.08) |  | 2.02(0.94 to 3.74) | 3.12(1.9 to 4.8) |  | 1.22(0.94 to 1.49) |  | 113.24(63.57 to 183.63) | 117.07(85.61 to 156.88) |  | -0.17(-0.38 to 0.05) |  |
| **Fiji** | Both | 206.74(133.13 to 312.78) | 218.7(134.91 to 340.39) |  | 0.23(0.02 to 0.45) |  | 6.02(3.12 to 10.52) | 6.24(3.05 to 11.07) |  | -0.14(-0.44 to 0.15) |  | 200.72(129.19 to 303.6) | 212.46(131.57 to 329.96) |  | 0.24(0.03 to 0.46) |  |
| **Finland** | Both | 86.9(70.65 to 105.38) | 47.66(37.8 to 58.67) |  | -1.96(-2.17 to -1.75) |  | 5.95(3.45 to 9.24) | 5.95(3.35 to 9.74) |  | 0.12(-0.03 to 0.27) |  | 80.95(65.92 to 98.07) | 41.71(33.32 to 50.91) |  | -2.18(-2.4 to -1.96) |  |
| **France** | Both | 95.71(79.32 to 114.7) | 69.15(55.87 to 84.05) |  | -1.15(-1.31 to -0.99) |  | 6.43(3.77 to 9.92) | 9.12(5.33 to 14.16) |  | 1.25(1.02 to 1.47) |  | 89.28(73.88 to 106.39) | 60.03(49.15 to 72.4) |  | -1.41(-1.57 to -1.24) |  |
| **Gabon** | Both | 90.29(54.28 to 138.53) | 119.46(67.39 to 195.33) |  | 0.64(0.45 to 0.84) |  | 1.93(0.93 to 3.39) | 3.58(1.58 to 6.83) |  | 1.77(1.62 to 1.91) |  | 88.36(53.09 to 135.59) | 115.88(65.4 to 189.85) |  | 0.62(0.42 to 0.81) |  |
| **Gambia** | Both | 25.66(14.49 to 41.5) | 44.43(26.25 to 72.67) |  | 1.48(1.17 to 1.78) |  | 0.57(0.26 to 1.05) | 1.21(0.53 to 2.27) |  | 2.16(1.86 to 2.47) |  | 25.09(14.17 to 40.59) | 43.22(25.51 to 70.2) |  | 1.46(1.15 to 1.76) |  |
| **Georgia** | Both | 178.51(158.63 to 199.94) | 94.24(78.97 to 109.32) |  | -1.83(-2.29 to -1.38) |  | 7.15(4.19 to 10.89) | 4.59(2.77 to 7.11) |  | -1.41(-1.87 to -0.94) |  | 171.36(152.24 to 191.57) | 89.65(75.37 to 103.89) |  | -1.85(-2.31 to -1.4) |  |
| **Germany** | Both | 108.15(90.08 to 129.12) | 64.94(53.21 to 78) |  | -1.58(-1.9 to -1.26) |  | 6.3(3.71 to 9.47) | 6.92(3.98 to 10.65) |  | 0.31(0.15 to 0.46) |  | 101.85(84.86 to 121.3) | 58.01(47.85 to 69.71) |  | -1.75(-2.09 to -1.41) |  |
| **Ghana** | Both | 116.83(71.9 to 181.53) | 125.17(72.96 to 195.77) |  | 0.02(-0.09 to 0.14) |  | 2.53(1.21 to 4.43) | 3.5(1.63 to 6.42) |  | 0.81(0.69 to 0.94) |  | 114.3(70.32 to 177.46) | 121.67(70.93 to 191.16) |  | 0(-0.11 to 0.12) |  |
| **Greece** | Both | 104.13(90.68 to 118.62) | 61.3(52.69 to 70.64) |  | -1.63(-1.82 to -1.43) |  | 7.8(4.53 to 11.94) | 6.21(3.6 to 9.36) |  | -0.68(-0.86 to -0.5) |  | 96.33(84.61 to 108.62) | 55.09(47.73 to 63.33) |  | -1.72(-1.92 to -1.52) |  |
| **Greenland** | Both | 118.86(80.79 to 167.47) | 62.18(35.67 to 96.92) |  | -2.43(-2.65 to -2.22) |  | 3.94(1.98 to 6.5) | 3.14(1.42 to 5.78) |  | -0.91(-1.13 to -0.7) |  | 114.92(78 to 161.87) | 59.03(33.81 to 91.06) |  | -2.5(-2.72 to -2.28) |  |
| **Grenada** | Both | 167.24(129.56 to 212.02) | 146.33(106.68 to 193.55) |  | -0.65(-0.97 to -0.32) |  | 5.02(2.79 to 8.15) | 6.23(3.34 to 10.16) |  | 0.27(-0.01 to 0.55) |  | 162.22(126.07 to 206.26) | 140.1(102.25 to 184.37) |  | -0.68(-1.01 to -0.36) |  |
| **Guam** | Both | 68.93(49.11 to 95.44) | 114.39(84.87 to 151.58) |  | 2.09(1.63 to 2.55) |  | 3.18(1.76 to 5.06) | 5.05(2.9 to 8.37) |  | 1.86(1.27 to 2.45) |  | 65.75(47 to 91.27) | 109.34(81.44 to 144.54) |  | 2.1(1.64 to 2.55) |  |
| **Guatemala** | Both | 42.04(35.55 to 49.57) | 54.65(42.98 to 68.35) |  | 0.75(0.39 to 1.12) |  | 1.21(0.7 to 1.87) | 2.65(1.55 to 4.1) |  | 2.42(2.09 to 2.76) |  | 40.83(34.61 to 48.14) | 52.01(40.93 to 64.68) |  | 0.68(0.32 to 1.05) |  |
| **Guinea** | Both | 56.15(34.39 to 87.12) | 85.13(45.99 to 143.83) |  | 1.4(1.33 to 1.47) |  | 1.08(0.51 to 1.9) | 1.97(0.88 to 3.79) |  | 1.94(1.87 to 2.02) |  | 55.07(33.8 to 85.44) | 83.16(44.86 to 140.24) |  | 1.39(1.32 to 1.46) |  |
| **Guinea-Bissau** | Both | 100.21(59.38 to 158.17) | 128.27(76.04 to 202.09) |  | 0.87(0.84 to 0.9) |  | 1.81(0.9 to 3.17) | 2.84(1.37 to 5.2) |  | 1.5(1.43 to 1.56) |  | 98.41(58.14 to 155.71) | 125.44(74.17 to 197.51) |  | 0.86(0.83 to 0.89) |  |
| **Guyana** | Both | 93.76(68.07 to 123.11) | 146.83(93.46 to 216.5) |  | 1.53(1.14 to 1.93) |  | 2.28(1.34 to 3.59) | 4.51(2.28 to 7.4) |  | 2.13(1.72 to 2.54) |  | 91.48(66.47 to 120.11) | 142.32(90.06 to 209.35) |  | 1.52(1.12 to 1.91) |  |
| **Haiti** | Both | 127.48(57.24 to 238.37) | 133.3(67.36 to 229) |  | 0.33(0.25 to 0.42) |  | 2.4(0.93 to 4.9) | 3.03(1.18 to 6.01) |  | 0.9(0.83 to 0.97) |  | 125.08(56.23 to 233.62) | 130.27(65.95 to 223.06) |  | 0.32(0.24 to 0.41) |  |
| **Honduras** | Both | 44.07(28.4 to 65.98) | 45.11(23.36 to 77.17) |  | -0.34(-0.57 to -0.11) |  | 1.36(0.66 to 2.3) | 1.95(0.79 to 3.74) |  | 0.69(0.41 to 0.96) |  | 42.7(27.39 to 63.8) | 43.16(22.47 to 73.89) |  | -0.38(-0.61 to -0.15) |  |
| **Hungary** | Both | 110.63(89.46 to 134.45) | 64.47(50.49 to 80.42) |  | -1.83(-2.17 to -1.48) |  | 5(2.84 to 7.77) | 5.18(2.92 to 8.13) |  | -0.08(-0.32 to 0.16) |  | 105.63(85.31 to 127.85) | 59.29(46.5 to 73.54) |  | -1.94(-2.29 to -1.59) |  |
| **Iceland** | Both | 92.29(74.39 to 113.15) | 53.31(42.03 to 66.8) |  | -1.2(-1.71 to -0.69) |  | 7.09(4.22 to 10.96) | 6.31(3.59 to 10.25) |  | 0.15(-0.24 to 0.54) |  | 85.2(69.08 to 103.77) | 47(37.3 to 58.01) |  | -1.35(-1.87 to -0.83) |  |
| **India** | Both | 58.62(48.7 to 69.77) | 80.36(65.21 to 98.27) |  | 1.06(0.95 to 1.16) |  | 1.27(0.85 to 1.78) | 2.62(1.68 to 3.77) |  | 2.45(2.35 to 2.55) |  | 57.35(47.65 to 68.33) | 77.74(63.1 to 95.02) |  | 1.02(0.91 to 1.13) |  |
| **Indonesia** | Both | 80.75(54.53 to 117.43) | 95.58(68.21 to 134.9) |  | 0.38(0.23 to 0.54) |  | 1.95(1.08 to 3.17) | 3.22(1.98 to 4.89) |  | 1.45(1.33 to 1.57) |  | 78.8(53.37 to 114.64) | 92.36(65.73 to 131.01) |  | 0.35(0.19 to 0.51) |  |
| **Iran (Islamic Republic of)** | Both | 53.59(43.86 to 65.76) | 67.84(58.63 to 78.76) |  | 1.12(0.84 to 1.39) |  | 3.13(1.99 to 4.61) | 7.33(4.83 to 10.61) |  | 3(2.71 to 3.29) |  | 50.46(41.3 to 61.81) | 60.51(52.43 to 70.47) |  | 0.95(0.68 to 1.22) |  |
| **Iraq** | Both | 65.13(41.97 to 97.05) | 82.21(52.36 to 125.83) |  | 0.65(0.48 to 0.81) |  | 2.68(1.36 to 4.73) | 5.66(2.89 to 9.88) |  | 2.51(2.37 to 2.64) |  | 62.45(40.21 to 93.09) | 76.54(48.27 to 117.24) |  | 0.54(0.38 to 0.71) |  |
| **Ireland** | Both | 115.29(95.15 to 137.48) | 54.12(43.08 to 66.39) |  | -1.85(-2.07 to -1.63) |  | 7.35(4.41 to 11.32) | 6.59(3.79 to 10.24) |  | 0.51(0.24 to 0.78) |  | 107.95(88.72 to 128.66) | 47.53(37.82 to 57.85) |  | -2.08(-2.31 to -1.86) |  |
| **Israel** | Both | 130.06(108.83 to 152.93) | 58.84(48.89 to 70.33) |  | -2.15(-2.41 to -1.9) |  | 7.28(4.3 to 10.86) | 5.83(3.25 to 9.44) |  | -0.18(-0.41 to 0.06) |  | 122.78(102.47 to 144.17) | 53.01(44.11 to 62.96) |  | -2.32(-2.57 to -2.06) |  |
| **Italy** | Both | 117.09(109.76 to 124.43) | 53.82(49.21 to 58.61) |  | -2.6(-2.79 to -2.42) |  | 8.82(5.88 to 12.61) | 6.73(4.53 to 9.55) |  | -0.99(-1.17 to -0.8) |  | 108.26(102.3 to 114.2) | 47.09(43.73 to 50.74) |  | -2.79(-2.98 to -2.59) |  |
| **Jamaica** | Both | 80.31(63.92 to 99.18) | 137.22(89.91 to 199.21) |  | 1.32(0.86 to 1.79) |  | 3.1(1.88 to 4.75) | 6.61(3.3 to 11.49) |  | 2(1.56 to 2.45) |  | 77.21(61.32 to 95.33) | 130.61(85.41 to 189.14) |  | 1.29(0.82 to 1.76) |  |
| **Japan** | Both | 56.37(53.83 to 59.21) | 45.25(42.54 to 48.45) |  | -0.83(-0.99 to -0.68) |  | 4.85(3.25 to 6.75) | 5.92(3.94 to 8.4) |  | 0.64(0.41 to 0.86) |  | 51.51(49.63 to 53.36) | 39.33(37.79 to 40.97) |  | -1.01(-1.17 to -0.86) |  |
| **Jordan** | Both | 68.49(42.67 to 105.61) | 59.27(33.88 to 95.04) |  | -0.6(-1.09 to -0.12) |  | 3.22(1.48 to 5.69) | 5.11(2.31 to 9.19) |  | 1.59(1.08 to 2.11) |  | 65.27(40.79 to 100.32) | 54.16(30.98 to 87.3) |  | -0.75(-1.24 to -0.27) |  |
| **Kazakhstan** | Both | 126.19(111.4 to 143.36) | 54.14(46.1 to 62.42) |  | -2.24(-2.52 to -1.96) |  | 4.27(2.53 to 6.43) | 2.85(1.68 to 4.45) |  | -0.55(-0.78 to -0.33) |  | 121.92(107.88 to 138.29) | 51.29(43.7 to 58.9) |  | -2.31(-2.6 to -2.03) |  |
| **Kenya** | Both | 59.54(39.18 to 87.67) | 107.2(64.49 to 167.25) |  | 1.99(1.92 to 2.05) |  | 1.39(0.81 to 2.23) | 3.06(1.61 to 5.21) |  | 2.47(2.39 to 2.55) |  | 58.15(38.24 to 85.58) | 104.13(62.79 to 162.27) |  | 1.97(1.9 to 2.04) |  |
| **Kiribati** | Both | 171.51(110.02 to 256.23) | 225.83(131.07 to 366.15) |  | 1.02(0.97 to 1.07) |  | 3.49(1.74 to 6.15) | 4.86(2.32 to 8.57) |  | 1.05(0.99 to 1.11) |  | 168.02(107.48 to 250.95) | 220.97(128.32 to 358.3) |  | 1.02(0.97 to 1.07) |  |
| **Kuwait** | Both | 30.21(23.2 to 38.34) | 49.96(37.8 to 64.48) |  | -0.7(-1.56 to 0.17) |  | 2.37(1.35 to 3.84) | 6.03(3.34 to 9.77) |  | 0.82(-0.04 to 1.69) |  | 27.84(21.47 to 35.1) | 43.93(33.82 to 55.95) |  | -0.86(-1.73 to 0.01) |  |
| **Kyrgyzstan** | Both | 103.21(79.56 to 128.1) | 62(46.1 to 79.99) |  | -1.93(-2.13 to -1.73) |  | 3.24(1.97 to 5.02) | 2.83(1.51 to 4.6) |  | -0.64(-0.92 to -0.37) |  | 99.97(76.98 to 124.37) | 59.17(44.04 to 76.22) |  | -1.98(-2.18 to -1.78) |  |
| **Lao People's Democratic Republic** | Both | 70.41(32.04 to 130.85) | 96.96(57.59 to 157.19) |  | 1.08(0.96 to 1.2) |  | 1.36(0.48 to 2.82) | 2.7(1.3 to 4.94) |  | 2.37(2.29 to 2.45) |  | 69.05(31.37 to 128.38) | 94.26(56.14 to 152.9) |  | 1.05(0.93 to 1.18) |  |
| **Latvia** | Both | 88.68(71.32 to 109.78) | 47.13(34.79 to 61.06) |  | -2.34(-2.7 to -1.99) |  | 3.45(1.97 to 5.45) | 2.65(1.52 to 4.41) |  | -1.12(-1.45 to -0.8) |  | 85.23(68.66 to 105.61) | 44.48(32.85 to 57.28) |  | -2.4(-2.76 to -2.04) |  |
| **Lebanon** | Both | 80.23(45.12 to 129.95) | 70.18(45.97 to 100.43) |  | -0.23(-0.37 to -0.09) |  | 4.09(1.83 to 7.65) | 6.84(3.58 to 12.19) |  | 1.94(1.76 to 2.13) |  | 76.13(42.9 to 123.38) | 63.34(41.6 to 89.4) |  | -0.4(-0.54 to -0.26) |  |
| **Lesotho** | Both | 54.21(25.43 to 101.7) | 105.91(56.07 to 175.72) |  | 3.29(2.74 to 3.83) |  | 1.16(0.42 to 2.36) | 2.45(1.11 to 4.59) |  | 3.36(2.86 to 3.87) |  | 53.06(24.88 to 99.55) | 103.46(54.68 to 172.14) |  | 3.28(2.74 to 3.83) |  |
| **Liberia** | Both | 48.49(27.92 to 79.13) | 79.96(43.61 to 137.97) |  | 1.39(1.18 to 1.6) |  | 0.95(0.48 to 1.67) | 2.16(0.92 to 4.09) |  | 2.55(2.37 to 2.72) |  | 47.54(27.29 to 77.52) | 77.8(42.35 to 134.47) |  | 1.37(1.16 to 1.57) |  |
| **Libya** | Both | 37.94(23.34 to 57.53) | 59.42(35.24 to 95.51) |  | 1.28(1.05 to 1.51) |  | 1.81(0.88 to 3) | 3.99(1.79 to 7.44) |  | 2.46(2.12 to 2.8) |  | 36.12(22.11 to 54.7) | 55.43(32.99 to 88.28) |  | 1.2(0.98 to 1.43) |  |
| **Lithuania** | Both | 85.55(68.35 to 104.5) | 51.08(37.06 to 65.9) |  | -1.24(-1.63 to -0.84) |  | 4.31(2.46 to 6.96) | 3.53(1.85 to 5.72) |  | -0.33(-0.66 to -0.01) |  | 81.23(65.04 to 98.79) | 47.55(34.66 to 61.28) |  | -1.3(-1.7 to -0.9) |  |
| **Luxembourg** | Both | 120.18(103.54 to 138.85) | 44.14(36.51 to 52.07) |  | -3.54(-3.78 to -3.29) |  | 7.29(4.3 to 11.11) | 5.04(2.91 to 7.88) |  | -1.41(-1.76 to -1.06) |  | 112.9(97.47 to 130.08) | 39.1(32.83 to 45.91) |  | -3.74(-3.98 to -3.5) |  |
| **Madagascar** | Both | 100.96(60.69 to 157.4) | 124.58(75.78 to 194.72) |  | 0.55(0.37 to 0.74) |  | 2(0.96 to 3.56) | 3(1.5 to 5.24) |  | 1.16(0.95 to 1.36) |  | 98.96(59.57 to 154.39) | 121.58(73.86 to 190.25) |  | 0.54(0.36 to 0.73) |  |
| **Malawi** | Both | 67.98(42.54 to 101.04) | 117.74(67.84 to 193.05) |  | 1.92(1.61 to 2.23) |  | 1.33(0.67 to 2.31) | 2.92(1.38 to 5.48) |  | 2.68(2.34 to 3.01) |  | 66.65(41.77 to 99.17) | 114.83(66.26 to 188.37) |  | 1.9(1.59 to 2.22) |  |
| **Malaysia** | Both | 97.52(65.24 to 135.74) | 107.36(72.89 to 151.47) |  | 0.27(0.07 to 0.47) |  | 2.9(1.54 to 4.81) | 5.09(2.59 to 8.64) |  | 1.9(1.74 to 2.06) |  | 94.62(63.28 to 131.91) | 102.27(69.66 to 143.63) |  | 0.21(0.01 to 0.4) |  |
| **Maldives** | Both | 41.1(14.32 to 87.15) | 23.64(14.36 to 36.51) |  | -2.08(-2.65 to -1.51) |  | 1.05(0.26 to 2.49) | 1.31(0.66 to 2.35) |  | 0.64(-0.09 to 1.38) |  | 40.05(13.87 to 85.01) | 22.33(13.61 to 34.58) |  | -2.19  (-2.76 to -1.62) |  |
| **Mali** | Both | 72.02(47.31 to 108.72) | 80.39(44.61 to 134.06) |  | 0.25(0.11 to 0.39) |  | 1.42(0.72 to 2.49) | 1.96(0.88 to 3.59) |  | 0.96(0.83 to 1.08) |  | 70.59(46.31 to 106.71) | 78.43(43.6 to 130.59) |  | 0.23(0.09 to 0.37) |  |
| **Malta** | Both | 109.26(89.14 to 131.44) | 63.14(50.39 to 77.52) |  | -0.92(-1.36 to -0.48) |  | 6.32(3.75 to 9.91) | 6.22(3.49 to 10) |  | 0.91  (0.51 to 1.3) |  | 102.94(83.77 to 124.06) | 56.92(45.58 to 69.4) |  | -1.07(-1.51 to -0.63) |  |
| **Marshall Islands** | Both | 132.54(75.05 to 215.39) | 199.55(97.27 to 357.09) |  | 1.26(1.1 to 1.43) |  | 3.12(1.37 to 5.66) | 4.98(2.02 to 9.79) |  | 1.27(1.16 to 1.38) |  | 129.42(73.5 to 210.4) | 194.57(94.32 to 346.64) |  | 1.26(1.09 to 1.43) |  |
| **Mauritania** | Both | 48.15(26.11 to 77.49) | 73.32(44.2 to 117.07) |  | 1.25(1.11 to 1.39) |  | 0.99(0.4 to 1.84) | 2.34(1.13 to 4.29) |  | 2.64(2.5 to 2.78) |  | 47.16(25.6 to 75.94) | 70.97(42.59 to 113.1) |  | 1.21(1.08 to 1.35) |  |
| **Mauritius** | Both | 59.95(51.05 to 70.23) | 121.48(100.87 to 142.51) |  | 1.31(0.93 to 1.7) |  | 2.1(1.21 to 3.18) | 5.88(3.56 to 8.8) |  | 2.26(1.88 to 2.64) |  | 57.86(49.48 to 67.51) | 115.61(96.14 to 135.19) |  | 1.27(0.89 to 1.66) |  |
| **Mexico** | Both | 76.64(73.12 to 80.45) | 73.04(59.73 to 87.33) |  | -0.34(-0.49 to -0.18) |  | 3.09(2.23 to 4.16) | 4.75(3.18 to 6.86) |  | 1.06(0.9 to 1.21) |  | 73.54(70.23 to 77.27) | 68.3(55.92 to 81.68) |  | -0.41(-0.57 to -0.26) |  |
| **Micronesia (Federated States of)** | Both | 148.72(82.54 to 240.65) | 184.46(104.29 to 290.65) |  | 0.65(0.57 to 0.72) |  | 3.46(1.59 to 6.41) | 4.98(2.33 to 8.96) |  | 1.04(0.95 to 1.13) |  | 145.27(80.2 to 235.52) | 179.48(101.5 to 281.55) |  | 0.64(0.56 to 0.71) |  |
| **Monaco** | Both | 148.86(100.77 to 208.81) | 175.88(104.76 to 273.86) |  | 0.72(0.57 to 0.87) |  | 11.81(6.06 to 19.67) | 19.81(10.24 to 35.79) |  | 1.94(1.7 to 2.18) |  | 137.05(92.92 to 191.56) | 156.07(92.94 to 241.11) |  | 0.59(0.45 to 0.74) |  |
| **Mongolia** | Both | 32.43(21.33 to 47.46) | 33.47(22.01 to 48.19) |  | -0.12(-0.32 to 0.08) |  | 0.77(0.38 to 1.3) | 1.24(0.63 to 2.11) |  | 1.61(1.45 to 1.77) |  | 31.67(20.83 to 46.18) | 32.23(21.12 to 46.43) |  | -0.17(-0.38 to 0.04) |  |
| **Montenegro** | Both | 100.09(75.48 to 133.88) | 73.68(53.09 to 99.16) |  | -0.99(-1.44 to -0.53) |  | 6.21(3.57 to 9.95) | 6.01(3.32 to 9.64) |  | 0.21(-0.14 to 0.56) |  | 93.88(70.8 to 126.22) | 67.68(48.59 to 91.1) |  | -1.08(-1.54 to -0.61) |  |
| **Morocco** | Both | 37.09(21.54 to 59.95) | 62.35(36.41 to 107.32) |  | 1.67(1.43 to 1.91) |  | 1.3(0.6 to 2.35) | 3.39(1.6 to 6.52) |  | 3.07(2.86 to 3.27) |  | 35.8(20.84 to 57.9) | 58.96(34.21 to 101.25) |  | 1.61(1.37 to 1.85) |  |
| **Mozambique** | Both | 102.72(63.12 to 154.68) | 149.6(84.05 to 249.74) |  | 1.43(1.31 to 1.55) |  | 1.94(0.92 to 3.41) | 3.39(1.59 to 6.38) |  | 1.98(1.87 to 2.1) |  | 100.78(62.1 to 151.48) | 146.21(81.89 to 244.38) |  | 1.42(1.29 to 1.54) |  |
| **Myanmar** | Both | 178.44(93.65 to 299.66) | 153.56(96.42 to 230.27) |  | -0.86(-1.05 to -0.66) |  | 3.63(1.42 to 6.88) | 4.81(2.43 to 8.68) |  | 0.62(0.48 to 0.75) |  | 174.82(91.63 to 293.48) | 148.75(93.32 to 222.48) |  | -0.89(-1.09 to -0.7) |  |
| **Namibia** | Both | 83.95(53.08 to 126.89) | 126.57(71.34 to 211.35) |  | 1.33(1.23 to 1.44) |  | 1.85(0.89 to 3.32) | 3.79(1.71 to 7.27) |  | 2.36(2.25 to 2.46) |  | 82.1(51.89 to 123.94) | 122.79(69.41 to 205.02) |  | 1.31(1.21 to 1.41) |  |
| **Nauru** | Both | 196.41(90.47 to 357.95) | 241.74(110.76 to 450.08) |  | 0.7(0.65 to 0.74) |  | 5.05(1.89 to 10.01) | 6.71(2.4 to 13.56) |  | 0.71(0.51 to 0.91) |  | 191.37(88.3 to 348.62) | 235.04(108.03 to 438.19) |  | 0.69(0.65 to 0.74) |  |
| **Nepal** | Both | 45.23(25.97 to 72.37) | 63.19(36 to 104.93) |  | 1.21(1.03 to 1.38) |  | 0.9(0.41 to 1.68) | 1.95(0.85 to 3.65) |  | 2.69(2.52 to 2.85) |  | 44.33(25.46 to 70.93) | 61.24(35.02 to 102) |  | 1.17(1 to 1.34) |  |
| **Netherlands** | Both | 121(101.3 to 141.29) | 67.74(54.72 to 82.29) |  | -2.08(-2.23 to -1.93) |  | 7.95(4.75 to 12.26) | 7.99(4.82 to 12.79) |  | 0.17(0.02 to 0.31) |  | 113.05(94.65 to 131.89) | 59.75(48.89 to 72.09) |  | -2.3(-2.47 to -2.14) |  |
| **New Zealand** | Both | 135.47(113.72 to 157.73) | 70.35(59.27 to 82.1) |  | -1.93(-2.11 to -1.75) |  | 8.13(5.03 to 12.22) | 6.69(4.3 to 9.68) |  | -0.36(-0.56 to -0.16) |  | 127.34(107.21 to 148.53) | 63.66(53.58 to 74.28) |  | -2.06(-2.24 to -1.88) |  |
| **Nicaragua** | Both | 47.28(33.34 to 64.73) | 47.09(30.52 to 69.87) |  | 0.21(0.05 to 0.37) |  | 1.84(0.96 to 3.07) | 3.02(1.59 to 5.2) |  | 1.87(1.69 to 2.05) |  | 45.44(31.95 to 62.3) | 44.07(28.6 to 64.96) |  | 0.12(-0.04 to 0.28) |  |
| **Niger** | Both | 41.87(23.53 to 69.95) | 42.07(22.17 to 73.29) |  | -0.01(-0.13 to 0.11) |  | 0.8(0.39 to 1.53) | 0.97(0.39 to 1.99) |  | 0.71(0.61 to 0.81) |  | 41.08(23.17 to 68.58) | 41.1(21.75 to 71.57) |  | -0.03(-0.15 to 0.09) |  |
| **Nigeria** | Both | 71.28(49.09 to 102.09) | 108.65(61.18 to 178.92) |  | 1.25(1.1 to 1.41) |  | 1.48(0.85 to 2.4) | 2.98(1.46 to 5.1) |  | 2.24(2.07 to 2.42) |  | 69.8(48.13 to 100.08) | 105.67(59.58 to 174.23) |  | 1.23(1.07 to 1.38) |  |
| **Niue** | Both | 146.65(88.57 to 233.12) | 266.62(172.46 to 391.6) |  | 0.77(0.35 to 1.19) |  | 4.66(2.17 to 8.43) | 9.76(4.91 to 16.93) |  | 1.06(0.61 to 1.52) |  | 141.99(85.43 to 224.91) | 256.86(166.72 to 376.18) |  | 0.76(0.34 to 1.18) |  |
| **North Macedonia** | Both | 115.35(89.12 to 146.18) | 65.17(47.04 to 89.69) |  | -1.84(-2.07 to -1.6) |  | 4.46(2.53 to 7.05) | 4.25(2.29 to 7.04) |  | 0.11(-0.21 to 0.43) |  | 110.89(85.76 to 140.07) | 60.93(43.99 to 83.6) |  | -1.94(-2.18 to -1.71) |  |
| **Northern Mariana Islands** | Both | 132(74.15 to 209.8) | 130.27(84.95 to 186.35) |  | -0.08(-0.53 to 0.37) |  | 5.7(2.46 to 10.16) | 5.74(2.88 to 9.64) |  | -0.43(-0.91 to 0.06) |  | 126.3(70.99 to 200.97) | 124.52(81.05 to 177.62) |  | -0.07(-0.51 to 0.38) |  |
| **Norway** | Both | 85.36(79.03 to 91.86) | 34.97(31.59 to 38.73) |  | -3.07(-3.29 to -2.86) |  | 5.26(3.59 to 7.51) | 3.96(2.6 to 5.79) |  | -0.92(-1.26 to -0.57) |  | 80.1(74.44 to 86.1) | 31(28.24 to 34.03) |  | -3.28(-3.49 to -3.07) |  |
| **Oman** | Both | 8.6(5.05 to 13.57) | 8.8(5.6 to 13.59) |  | 0.49(-0.21 to 1.19) |  | 0.42(0.19 to 0.77) | 0.81(0.41 to 1.43) |  | 2.33(1.6 to 3.07) |  | 8.19(4.78 to 12.93) | 7.99(5.1 to 12.35) |  | 0.35(-0.35 to 1.06) |  |
| **Pakistan** | Both | 105.18(70.6 to 149.59) | 195.18(123.31 to 291.64) |  | 1.63(1.4 to 1.86) |  | 2.16(1.22 to 3.5) | 5.09(2.75 to 8.44) |  | 2.47(2.31 to 2.62) |  | 103.02(69.23 to 146.73) | 190.08(120.09 to 283.86) |  | 1.61(1.38 to 1.84) |  |
| **Palau** | Both | 234.61(133.18 to 376.04) | 212.23(128.61 to 327.73) |  | -0.17(-0.36 to 0.03) |  | 7.66(3.42 to 13.85) | 7.78(3.73 to 13.73) |  | -0.08(-0.24 to 0.09) |  | 226.95(128.56 to 364.62) | 204.45(124.77 to 315.28) |  | -0.17(-0.37 to 0.03) |  |
| **Palestine** | Both | 97.97(55.56 to 159.93) | 103.77(72.25 to 143.41) |  | 0.22(0.03 to 0.41) |  | 4.29(1.96 to 7.59) | 7.2(3.83 to 12.15) |  | 1.65(1.42 to 1.88) |  | 93.68(53.19 to 152.8) | 96.57(67.85 to 132.23) |  | 0.14(-0.05 to 0.32) |  |
| **Panama** | Both | 56.58(44.74 to 71.56) | 92.67(66.04 to 125.88) |  | 1.93(1.71 to 2.16) |  | 3.03(1.84 to 4.65) | 8.02(4.45 to 13.03) |  | 3.34(3.09 to 3.59) |  | 53.54(42.45 to 67.44) | 84.65(60.63 to 114.69) |  | 1.83(1.6 to 2.05) |  |
| **Papua New Guinea** | Both | 123.15(64.92 to 199.67) | 151(87.12 to 238.26) |  | 0.55(0.38 to 0.72) |  | 3.04(1.33 to 5.52) | 3.63(1.62 to 6.65) |  | 0.28(0.02 to 0.53) |  | 120.11(63.53 to 195.12) | 147.38(84.91 to 233.03) |  | 0.56(0.39 to 0.72) |  |
| **Paraguay** | Both | 69.61(46.7 to 98.65) | 77.29(49.71 to 119.56) |  | 0.4(0.27 to 0.53) |  | 2.15(1.13 to 3.5) | 3.3(1.57 to 5.8) |  | 1.4(1.27 to 1.53) |  | 67.46(44.99 to 95.67) | 74(47.25 to 114.55) |  | 0.36(0.23 to 0.49) |  |
| **Peru** | Both | 71.34(50.86 to 98.45) | 54.07(33.67 to 82.19) |  | -1.13(-1.36 to -0.9) |  | 1.87(1.04 to 3.04) | 2.89(1.48 to 4.92) |  | 1.33(1.1 to 1.56) |  | 69.47(49.5 to 95.87) | 51.18(31.85 to 77.73) |  | -1.23(-1.45 to -1) |  |
| **Philippines** | Both | 111.44(96.06 to 127.88) | 134.44(101.69 to 175.06) |  | 0.75(0.63 to 0.88) |  | 3.01(1.98 to 4.18) | 4.46(2.76 to 6.82) |  | 1.29(1.13 to 1.46) |  | 108.44(93.4 to 124.19) | 129.99(98.4 to 168.56) |  | 0.74(0.62 to 0.86) |  |
| **Poland** | Both | 85.23(80.37 to 90.1) | 56.24(47.88 to 64.78) |  | -1.47(-1.75 to -1.2) |  | 3.45(2.35 to 4.87) | 4.28(2.7 to 6.18) |  | 0.66(0.45 to 0.87) |  | 81.78(77.34 to 86.28) | 51.97(44.11 to 59.55) |  | -1.6(-1.88 to -1.33) |  |
| **Portugal** | Both | 120.18(97.63 to 145.82) | 71.12(57.01 to 86.6) |  | -2.21(-2.46 to -1.97) |  | 7(4.15 to 10.59) | 8.18(4.72 to 12.9) |  | 0.05(-0.29 to 0.39) |  | 113.18(92.46 to 137.52) | 62.95(50.71 to 76.02) |  | -2.42(-2.67 to -2.18) |  |
| **Puerto Rico** | Both | 110.35(87.91 to 135.01) | 66.08(48.39 to 87.78) |  | -1.54(-1.95 to -1.12) |  | 5.32(3 to 8.35) | 5.24(2.86 to 8.54) |  | 0.18(-0.19 to 0.56) |  | 105.03(83.9 to 128.11) | 60.84(44.57 to 81.02) |  | -1.65(-2.07 to -1.24) |  |
| **Qatar** | Both | 52.74(32.82 to 79.2) | 35.57(21.74 to 56.54) |  | -2.14(-2.79 to -1.49) |  | 2.94(1.39 to 5.01) | 4.16(1.94 to 7.7) |  | 0.29(-0.39 to 0.97) |  | 49.8(31.14 to 74.27) | 31.41(19.22 to 49.77) |  | -2.36(-3 to -1.71) |  |
| **Republic of Korea** | Both | 51.43(38.13 to 66.4) | 46.55(34.02 to 62.8) |  | -0.47(-0.62 to -0.32) |  | 2.3(1.26 to 3.82) | 5.65(3 to 9.31) |  | 3.02(2.61 to 3.43) |  | 49.13(36.64 to 63.52) | 40.9(29.89 to 55.25) |  | -0.77(-0.92 to -0.63) |  |
| **Republic of Moldova** | Both | 102.73(88.51 to 119.51) | 54.32(44.48 to 66.35) |  | -1.9(-2.16 to -1.63) |  | 3.78(2.23 to 5.88) | 3.12(1.84 to 4.94) |  | -0.55(-0.87 to -0.22) |  | 98.95(84.83 to 114.77) | 51.21(41.91 to 62.51) |  | -1.96(-2.23 to -1.7) |  |
| **Romania** | Both | 103.83(83.13 to 126.31) | 66.69(51.53 to 85.06) |  | -1.3(-1.61 to -1) |  | 3.77(2.23 to 5.93) | 4.42(2.5 to 7.01) |  | 0.77(0.51 to 1.04) |  | 100.06(80.64 to 121.92) | 62.27(47.95 to 80.09) |  | -1.41(-1.72 to -1.11) |  |
| **Russian Federation** | Both | 88.24(84.32 to 92.37) | 68.97(60.19 to 77.36) |  | -1.02(-1.19 to -0.85) |  | 3.5(2.43 to 4.81) | 4.22(2.94 to 5.78) |  | 0.59(0.31 to 0.87) |  | 84.75(81.28 to 88.39) | 64.74(56.37 to 72.62) |  | -1.1(-1.27 to -0.93) |  |
| **Rwanda** | Both | 145.82(82.16 to 239.9) | 127.59(73.42 to 211.07) |  | -0.96(-1.21 to -0.72) |  | 2.63(1.21 to 4.91) | 3.3(1.61 to 5.99) |  | 0.33(0.07 to 0.6) |  | 143.18(80.81 to 234.67) | 124.29(71.48 to 206.02) |  | -0.99(-1.24 to -0.75) |  |
| **Saint Kitts and Nevis** | Both | 266.74(222.69 to 314.97) | 74.46(50.69 to 100.73) |  | -4.11(-4.97 to -3.24) |  | 7.22(4.42 to 11.1) | 3.29(1.82 to 5.39) |  | -2.53(-3.24 to -1.82) |  | 259.51(216.16 to 306.02) | 71.17(48.75 to 95.55) |  | -4.17(-5.03 to -3.29) |  |
| **Saint Lucia** | Both | 140.15(118.89 to 164.53) | 112.35(86.66 to 142.7) |  | -0.39(-0.77 to 0) |  | 4.36(2.67 to 6.53) | 5.06(3.02 to 8.17) |  | 0.72(0.39 to 1.06) |  | 135.79(115.11 to 159.67) | 107.29(83.27 to 135.71) |  | -0.43(-0.81 to -0.04) |  |
| **Saint Vincent and the Grenadines** | Both | 154.44(129.41 to 182.87) | 149.54(118.45 to 184.59) |  | -0.61(-0.89 to -0.32) |  | 4.94(2.95 to 7.46) | 5.96(3.41 to 9.39) |  | 0.13(-0.16 to 0.41) |  | 149.5(125.37 to 177.06) | 143.58(113.61 to 176.56) |  | -0.63(-0.92 to -0.35) |  |
| **Samoa** | Both | 68.14(41.81 to 104.98) | 92.04(51.88 to 143.25) |  | 1.09(0.97 to 1.21) |  | 2.03(1.03 to 3.53) | 2.99(1.33 to 5.66) |  | 1.19(1.05 to 1.33) |  | 66.12(40.6 to 101.61) | 89.04(50.42 to 138.59) |  | 1.09(0.97 to 1.21) |  |
| **San Marino** | Both | 70.12(46.52 to 99.86) | 40.98(20.84 to 70.7) |  | -0.78(-1.18 to -0.38) |  | 5.86(3.04 to 10) | 4.77(1.83 to 9.11) |  | 0.28(-0.22 to 0.78) |  | 64.27(42.75 to 91.57) | 36.21(18.4 to 63.13) |  | -0.9(-1.29 to -0.51) |  |
| **Sao Tome and Principe** | Both | 54.76(29.65 to 87.26) | 82.48(44.46 to 142.62) |  | 1.11(0.73 to 1.5) |  | 1.25(0.56 to 2.28) | 2.69(1.23 to 5.22) |  | 2.33(1.98 to 2.67) |  | 53.52(29.02 to 85.56) | 79.79(43.01 to 137.73) |  | 1.08(0.7 to 1.47) |  |
| **Saudi Arabia** | Both | 38.98(23.38 to 59.33) | 51.15(30.71 to 78.93) |  | 0.93(0.71 to 1.14) |  | 1.21(0.59 to 2.09) | 3.14(1.5 to 5.62) |  | 3.31(3.14 to 3.49) |  | 37.77(22.54 to 57.39) | 48.01(28.87 to 74.29) |  | 0.82(0.59 to 1.04) |  |
| **Senegal** | Both | 60.93(36.37 to 94.23) | 82.95(47.22 to 139.89) |  | 1.02(0.84 to 1.19) |  | 1.27(0.61 to 2.32) | 2.25(1.01 to 4.2) |  | 1.79(1.62 to 1.97) |  | 59.66(35.63 to 92.15) | 80.7(45.79 to 136.06) |  | 1(0.82 to 1.18) |  |
| **Serbia** | Both | 105.07(75.5 to 145.72) | 64.49(44.94 to 88.36) |  | -1.68(-1.86 to -1.5) |  | 4.43(2.37 to 7.33) | 4.81(2.54 to 7.98) |  | 0.37(0.2 to 0.53) |  | 100.63(72.51 to 139.11) | 59.68(41.34 to 81.94) |  | -1.8(-1.99 to -1.62) |  |
| **Seychelles** | Both | 98.59(66.66 to 139.71) | 122.79(81.76 to 172.13) |  | 0.25(0.07 to 0.43) |  | 3.05(1.63 to 5.14) | 5.52(3 to 9.17) |  | 1.47(1.24 to 1.71) |  | 95.54(64.37 to 134.94) | 117.27(78.71 to 163.97) |  | 0.2(0.02 to 0.38) |  |
| **Sierra Leone** | Both | 47.84(25.04 to 81.79) | 79.39(42.28 to 132.13) |  | 1.94(1.82 to 2.07) |  | 0.97(0.39 to 1.89) | 1.99(0.88 to 3.88) |  | 2.61(2.47 to 2.75) |  | 46.87(24.41 to 80.15) | 77.4(41.13 to 128.28) |  | 1.93(1.8 to 2.05) |  |
| **Singapore** | Both | 84.99(69.09 to 102.58) | 40.09(32.27 to 48.56) |  | -2.52(-2.89 to -2.15) |  | 4.78(2.74 to 7.58) | 4.8(2.57 to 7.78) |  | 0.09(-0.23 to 0.42) |  | 80.2(65.69 to 96.81) | 35.29(28.64 to 42.9) |  | -2.77(-3.14 to -2.4) |  |
| **Slovakia** | Both | 80.5(59.65 to 105.65) | 52.98(35.48 to 74.17) |  | -1.37(-1.54 to -1.2) |  | 3.38(1.82 to 5.39) | 3.72(1.9 to 6.27) |  | 0.36(0.2 to 0.52) |  | 77.12(56.63 to 101.14) | 49.26(33.08 to 68.83) |  | -1.47(-1.64 to -1.31) |  |
| **Slovenia** | Both | 69.13(55.88 to 84.49) | 38.26(28.53 to 50.56) |  | -1.94(-2.27 to -1.61) |  | 4.16(2.25 to 6.57) | 4.17(2.34 to 6.9) |  | 0.26(-0.04 to 0.56) |  | 64.97(52.59 to 79.55) | 34.09(25.36 to 44.26) |  | -2.14(-2.47 to -1.8) |  |
| **Solomon Islands** | Both | 71.25(30.56 to 124.38) | 143.74(79.9 to 229.24) |  | 2.33(2.22 to 2.44) |  | 1.66(0.63 to 3.4) | 3.47(1.49 to 6.36) |  | 2.28(2.1 to 2.46) |  | 69.59(29.82 to 121.74) | 140.26(78.21 to 223.52) |  | 2.33(2.22 to 2.44) |  |
| **Somalia** | Both | 66.97(38.55 to 113.54) | 69.4(39.05 to 116.21) |  | -0.13(-0.28 to 0.02) |  | 1.21(0.56 to 2.24) | 1.37(0.6 to 2.45) |  | 0.14(-0.02 to 0.3) |  | 65.76(37.84 to 111.47) | 68.03(38.18 to 114.21) |  | -0.13(-0.28 to 0.02) |  |
| **South Africa** | Both | 140.35(115.33 to 168.4) | 107.66(82.16 to 136.62) |  | -0.7(-1.55 to 0.16) |  | 3.64(2.43 to 5.28) | 3.53(2.25 to 5.19) |  | 0.06(-0.74 to 0.86) |  | 136.71(112.41 to 164.28) | 104.13(79.4 to 132.19) |  | -0.72(-1.57 to 0.14) |  |
| **South Sudan** | Both | 64.22(36.31 to 104.97) | 86.79(49.84 to 139.58) |  | 1.01(0.6 to 1.42) |  | 1.28(0.58 to 2.36) | 2.07(0.93 to 3.67) |  | 1.65(1.24 to 2.06) |  | 62.94(35.67 to 102.93) | 84.71(48.75 to 136.17) |  | 1(0.58 to 1.41) |  |
| **Spain** | Both | 120.36(99.87 to 142.54) | 53.07(43.59 to 63.93) |  | -2.97(-3.2 to -2.74) |  | 7.88(4.58 to 12.24) | 6.09(3.52 to 9.42) |  | -1.17(-1.34 to -1.01) |  | 112.48(93.8 to 132.51) | 46.98(38.59 to 56.44) |  | -3.15(-3.39 to -2.91) |  |
| **Sri Lanka** | Both | 60.35(39.32 to 89.83) | 57.41(33.3 to 90.16) |  | -0.19(-0.3 to -0.07) |  | 1.88(0.97 to 3.21) | 3.33(1.51 to 6) |  | 1.93(1.82 to 2.05) |  | 58.47(37.93 to 86.66) | 54.08(31.3 to 85.43) |  | -0.28(-0.39 to -0.17) |  |
| **Sudan** | Both | 36.71(17.12 to 69.57) | 59.59(26.76 to 104.22) |  | 1.9(1.75 to 2.06) |  | 1.11(0.41 to 2.38) | 2.83(1.1 to 5.66) |  | 3.25(3.1 to 3.39) |  | 35.6(16.54 to 67.36) | 56.76(25.49 to 98.95) |  | 1.85(1.7 to 2) |  |
| **Suriname** | Both | 89.76(58.61 to 126.13) | 103.51(64.93 to 152.59) |  | 0.55(0.21 to 0.89) |  | 2.51(1.32 to 4.08) | 3.65(1.83 to 6.28) |  | 1.33(1 to 1.66) |  | 87.26(57.05 to 122.62) | 99.86(62.09 to 147.39) |  | 0.52(0.18 to 0.86) |  |
| **Sweden** | Both | 82.94(69.86 to 97.12) | 37.04(28.34 to 46.9) |  | -2.03(-2.24 to -1.83) |  | 6.01(3.83 to 8.96) | 4.23(2.49 to 6.81) |  | -0.52(-0.83 to -0.22) |  | 76.93(64.51 to 89.92) | 32.81(25.28 to 41.43) |  | -2.19(-2.39 to -1.99) |  |
| **Switzerland** | Both | 70.99(57.45 to 86.04) | 36.75(29.1 to 45.48) |  | -2.45(-2.84 to -2.05) |  | 5.43(3.12 to 8.49) | 4.57(2.64 to 7.09) |  | -0.86(-1.41 to -0.3) |  | 65.56(53.14 to 78.77) | 32.17(25.77 to 39.54) |  | -2.62(-3.01 to -2.24) |  |
| **Syrian Arab Republic** | Both | 66.8(40.57 to 103.92) | 84.2(54.05 to 128.6) |  | 0.43(0.09 to 0.78) |  | 2.85(1.41 to 5.03) | 6.49(3.18 to 11.67) |  | 2.38(2.17 to 2.59) |  | 63.95(38.91 to 99.82) | 77.71(49.96 to 118.53) |  | 0.31(-0.04 to 0.67) |  |
| **Taiwan (Province of China)** | Both | 75.48(61.21 to 89.9) | 61.19(48.82 to 75.85) |  | -0.97(-1.17 to -0.78) |  | 3.85(2.21 to 6.14) | 5.63(3.15 to 9.01) |  | 0.98(0.68 to 1.29) |  | 71.64(58.44 to 85.18) | 55.56(44.37 to 68.63) |  | -1.13(-1.32 to -0.93) |  |
| **Tajikistan** | Both | 95.87(68.29 to 127.92) | 68.75(35.54 to 117.48) |  | -1.38(-1.65 to -1.11) |  | 2.69(1.51 to 4.28) | 2.33(0.99 to 4.58) |  | -0.83(-1.18 to -0.47) |  | 93.19(66.33 to 124.31) | 66.42(34.3 to 113.38) |  | -1.4(-1.66 to -1.13) |  |
| **Thailand** | Both | 71.28(46.17 to 101.69) | 123.2(81.04 to 180.68) |  | 1.38(0.85 to 1.92) |  | 2.37(1.28 to 4.03) | 7.3(3.75 to 12.43) |  | 3.37(2.83 to 3.91) |  | 68.9(44.79 to 98.26) | 115.9(76.06 to 169.83) |  | 1.29(0.76 to 1.82) |  |
| **Timor-Leste** | Both | 35.12(17.95 to 63.48) | 63.51(38.09 to 100.34) |  | 1.97(1.33 to 2.61) |  | 0.76(0.28 to 1.51) | 1.84(0.81 to 3.45) |  | 3.13(2.49 to 3.76) |  | 34.36(17.55 to 62.09) | 61.67(37.08 to 97.7) |  | 1.94(1.3 to 2.58) |  |
| **Togo** | Both | 76.26(47.26 to 120.28) | 100.12(54.23 to 171.22) |  | 0.98(0.78 to 1.17) |  | 1.62(0.8 to 2.97) | 2.67(1.24 to 5.08) |  | 1.66(1.41 to 1.92) |  | 74.65(46.34 to 117.61) | 97.45(52.91 to 166.69) |  | 0.96(0.77 to 1.16) |  |
| **Tokelau** | Both | 175.66(96.79 to 278.19) | 280.76(178.12 to 414.27) |  | 0.63(0.26 to 1.01) |  | 4.79(2.09 to 8.96) | 9.78(4.97 to 16.83) |  | 1.25(0.82 to 1.68) |  | 170.87(94.39 to 270.58) | 270.98(171.71 to 399.88) |  | 0.61(0.24 to 0.99) |  |
| **Tonga** | Both | 183.78(118.61 to 271.56) | 205.25(126.05 to 311.46) |  | 0.33(0.22 to 0.43) |  | 5.81(2.91 to 10.03) | 7(3.45 to 12.27) |  | 0.34(0.16 to 0.53) |  | 177.97(114.59 to 262.55) | 198.25(122.01 to 300.25) |  | 0.33(0.22 to 0.43) |  |
| **Trinidad and Tobago** | Both | 112.38(96.79 to 130.9) | 131.35(90.19 to 182.29) |  | 0.06(-0.15 to 0.27) |  | 3.6(2.21 to 5.5) | 5.9(3.08 to 9.57) |  | 1.34(1.16 to 1.53) |  | 108.78(93.75 to 126.68) | 125.45(86.24 to 173.51) |  | 0.01(-0.2 to 0.22) |  |
| **Tunisia** | Both | 46.97(31.57 to 67.79) | 58.64(35.28 to 90.74) |  | 0.7(0.51 to 0.88) |  | 2.38(1.25 to 4.09) | 4.88(2.42 to 8.79) |  | 2.28(2.05 to 2.5) |  | 44.6(29.95 to 64.42) | 53.77(32.57 to 83.84) |  | 0.59(0.4 to 0.77) |  |
| **Turkmenistan** | Both | 95.05(81.97 to 109.45) | 79.07(57.71 to 108.04) |  | -0.27(-0.61 to 0.07) |  | 2.81(1.67 to 4.45) | 3.16(1.77 to 5.31) |  | 0.73(0.38 to 1.09) |  | 92.24(79.29 to 106.1) | 75.91(55.5 to 103.81) |  | -0.31(-0.65 to 0.03) |  |
| **Tuvalu** | Both | 155.82(81.47 to 260.38) | 159.53(90.33 to 260.7) |  | -0.04(-0.09 to 0.01) |  | 3.62(1.5 to 6.81) | 4.57(2.16 to 8.27) |  | 0.43(0.31 to 0.56) |  | 152.2(79.53 to 254.33) | 154.95(87.82 to 253.33) |  | -0.05(-0.1 to 0) |  |
| **Türkiye** | Both | 25.53(15.78 to 38.69) | 76.72(50.96 to 108.24) |  | 5.8(4.73 to 6.88) |  | 1.04(0.49 to 1.84) | 6.65(3.28 to 11.31) |  | 8.56(7.43 to 9.71) |  | 24.49(15.12 to 37.1) | 70.07(46.61 to 98.92) |  | 5.62(4.55 to 6.7) |  |
| **Uganda** | Both | 75.62(43.08 to 122.22) | 139.39(84.31 to 216.63) |  | 0.96(0.55 to 1.36) |  | 1.6(0.75 to 2.99) | 3.58(1.66 to 6.51) |  | 1.7(1.35 to 2.05) |  | 74.02(42.14 to 119.24) | 135.81(82.38 to 210.68) |  | 0.94(0.53 to 1.35) |  |
| **Ukraine** | Both | 135.21(111.1 to 163) | 66.8(35.01 to 109.63) |  | -2.96(-3.26 to -2.67) |  | 5.22(3.11 to 8.19) | 3.17(1.38 to 5.89) |  | -2.17(-2.39 to -1.94) |  | 129.99(106.42 to 155.53) | 63.63(33.59 to 104.81) |  | -3(-3.3 to -2.7) |  |
| **United Arab Emirates** | Both | 37.81(22.71 to 59.73) | 39.89(23.38 to 63.61) |  | -0.34(-0.79 to 0.12) |  | 1.67(0.81 to 2.94) | 2.91(1.3 to 5.46) |  | 1.14(0.6 to 1.68) |  | 36.15(21.61 to 57.06) | 36.99(21.88 to 59.18) |  | -0.43(-0.87 to 0.02) |  |
| **United Kingdom** | Both | 130.65(126.52 to 135.1) | 66.56(63.53 to 70.29) |  | -2.08(-2.33 to -1.82) |  | 8.24(5.78 to 11.34) | 6.76(4.65 to 9.35) |  | -0.4(-0.64 to -0.15) |  | 122.41(119.15 to 125.5) | 59.8(57.69 to 62.02) |  | -2.23(-2.49 to -1.97) |  |
| **United Republic of Tanzania** | Both | 110.33(69.66 to 166.17) | 139.77(79.48 to 231.11) |  | 0.73(0.65 to 0.8) |  | 2.28(1.2 to 4.07) | 3.69(1.77 to 6.81) |  | 1.45(1.36 to 1.54) |  | 108.05(68.14 to 162.45) | 136.08(77.48 to 225.45) |  | 0.71(0.63 to 0.78) |  |
| **United States Virgin Islands** | Both | 158.77(105.55 to 226.68) | 148.62(83.39 to 256.29) |  | -0.11(-0.45 to 0.24) |  | 6.23(3.14 to 10.65) | 7.7(3.59 to 14.84) |  | 0.73(0.38 to 1.09) |  | 152.54(101.28 to 218.38) | 140.93(79.01 to 241.6) |  | -0.15(-0.49 to 0.2) |  |
| **United States of America** | Both | 113.84(109.42 to 118.92) | 66.73(62.2 to 71.35) |  | -1.8(-2.04 to -1.57) |  | 9.02(6.33 to 12.3) | 7.26(5.11 to 10) |  | -0.78(-0.92 to -0.65) |  | 104.82(101.64 to 108.38) | 59.47(55.98 to 62.84) |  | -1.91(-2.16 to -1.66) |  |
| **Uruguay** | Both | 130.22(104.9 to 158.73) | 111.95(89.19 to 137.64) |  | -0.91(-1.15 to -0.68) |  | 4.96(2.86 to 7.66) | 6.6(3.83 to 10.62) |  | 0.52(0.31 to 0.72) |  | 125.26(101.55 to 152.51) | 105.35(83.9 to 129.61) |  | -0.99(-1.22 to -0.75) |  |
| **Uzbekistan** | Both | 90.62(71.99 to 111.42) | 80.74(60.06 to 106.08) |  | -0.45(-0.67 to -0.22) |  | 2.86(1.73 to 4.43) | 3.26(1.86 to 5.12) |  | 0.46(0.2 to 0.73) |  | 87.76(69.69 to 107.87) | 77.48(57.84 to 101.64) |  | -0.48(-0.7 to -0.26) |  |
| **Vanuatu** | Both | 79.73(45.5 to 125.98) | 133.98(79.43 to 206.35) |  | 1.19(1.01 to 1.37) |  | 2(0.95 to 3.75) | 3.3(1.66 to 5.73) |  | 0.96(0.74 to 1.19) |  | 77.73(44.29 to 122.9) | 130.68(77.22 to 201.24) |  | 1.2(1.01 to 1.38) |  |
| **Venezuela (Bolivarian Republic of)** | Both | 86.18(74.29 to 100.55) | 119.64(82.87 to 165.12) |  | 0.83(0.38 to 1.29) |  | 3.64(2.15 to 5.63) | 7.61(4.11 to 12.49) |  | 2.25(1.86 to 2.64) |  | 82.54(71.25 to 95.79) | 112.03(78.05 to 155.52) |  | 0.75(0.29 to 1.21) |  |
| **Viet Nam** | Both | 55.2(33.01 to 84.72) | 60.97(38.47 to 92.25) |  | 0.29(0.16 to 0.42) |  | 1.57(0.73 to 2.79) | 3.13(1.55 to 5.53) |  | 2.25(2.11 to 2.38) |  | 53.62(32.07 to 82.5) | 57.85(36.73 to 87.03) |  | 0.21(0.08 to 0.34) |  |
| **Yemen** | Both | 21.06(10.28 to 37.53) | 38.43(21.4 to 64.37) |  | 2.12(1.87 to 2.37) |  | 0.65(0.24 to 1.28) | 1.57(0.71 to 3.03) |  | 3.11(2.84 to 3.38) |  | 20.42(9.99 to 36.15) | 36.85(20.53 to 61.66) |  | 2.08(1.83 to 2.34) |  |
| **Zambia** | Both | 128.68(71.96 to 205.32) | 247.1(102.04 to 480.23) |  | 2.31(1.84 to 2.79) |  | 2.51(1.14 to 4.56) | 6.17(2.22 to 13.1) |  | 3.18(2.59 to 3.77) |  | 126.17(70.59 to 201.52) | 240.93(99.4 to 467.76) |  | 2.29(1.82 to 2.77) |  |
| **Zimbabwe** | Both | 62.97(40.83 to 92.92) | 151.73(89.19 to 246.48) |  | 4.08(3.18 to 4.99) |  | 1.53(0.79 to 2.58) | 3.55(1.65 to 6.68) |  | 3.58(2.74 to 4.44) |  | 61.44(39.74 to 90.89) | 148.18(86.89 to 240.59) |  | 4.1(3.19 to 5.01) |  |

DALYs: disability-adjusted life years; YLDs: years lived with disability; YLLs: years of life lost; CI: confidence interval.

**Table S8.** Decomposition of the percentage change in the DALYs of adolescents and young adults with breast cancer in various regions of the world from 1990 to 2021.

| Characteristics | Sex | Overall difference | Population aging | Population growth | Epidemiological change |
| --- | --- | --- | --- | --- | --- |
| Global | Both | 913994.98 | 236946.58(25.92%) | 608248.96(66.55%) | 68799.45(7.53%) |
| SDI |  |  |  |  |  |
| High SDI | Both | -108902.01 | 27593.42(-25.34%) | 5313.35(-4.88%) | -141808.78(130.22%) |
| High-middle SDI | Both | -40424.62 | 76375.02(-188.93%) | -9677.85(23.94%) | -107121.8(264.99%) |
| Middle SDI | Both | 293702.63 | 135578.11(46.16%) | 127657.95(43.47%) | 30466.57(10.37%) |
| Low-middle SDI | Both | 505748.26 | 45694.46(9.04%) | 269081.17(53.2%) | 190972.64(37.76%) |
| Low SDI | Both | 263490.37 | -108.91(-0.04%) | 189397.33(71.88%) | 74201.95(28.16%) |
| Andean Latin America | Both | 7204.54 | 1976.87(27.44%) | 6758.73(93.81%) | -1531.06(-21.25%) |
| Australasia | Both | -1963.80 | 691.44(-35.21%) | 1954.09(-99.51%) | -4609.32(234.71%) |
| Caribbean | Both | 5588.16 | 2596.9(46.47%) | 3072.08(54.97%) | -80.82(-1.45%) |
| Central Asia | Both | -1433.13 | 5689.14(-396.97%) | 8165.49(-569.77%) | -15287.75(1066.74%) |
| Central Europe | Both | -21505.25 | 4350.98(-20.23%) | -10545(49.03%) | -15311.23(71.2%) |
| Central Latin America | Both | 36763.40 | 10501.86(28.57%) | 22448.19(61.06%) | 3813.35(10.37%) |
| Central Sub-Saharan Africa | Both | 28257.96 | 593.85(2.1%) | 21122.99(74.75%) | 6541.12(23.15%) |
| East Asia | Both | -30796.63 | 98457.88(-319.7%) | -54891.02(178.24%) | -74363.48(241.47%) |
| Eastern Europe | Both | -36121.04 | 13701.2(-37.93%) | -20069.78(55.56%) | -29752.46(82.37%) |
| Eastern Sub-Saharan Africa | Both | 129082.65 | 2686.81(2.08%) | 95302.1(73.83%) | 31093.74(24.09%) |
| High-income Asia Pacific | Both | -11135.55 | 5205.66(-46.75%) | -9457.62(84.93%) | -6883.59(61.82%) |
| High-income North America | Both | -51634.79 | -2218.69(4.3%) | 9636.45(-18.66%) | -59052.55(114.37%) |
| North Africa and Middle East | Both | 126176.80 | 26509.82(21.01%) | 64585.54(51.19%) | 35081.44(27.8%) |
| Oceania | Both | 5227.75 | 468.28(8.96%) | 3821.37(73.1%) | 938.09(17.94%) |
| South Asia | Both | 480377.88 | 36473.79(7.59%) | 261245.21(54.38%) | 182658.88(38.02%) |
| Southeast Asia | Both | 144235.73 | 37982.02(26.33%) | 72760.79(50.45%) | 33492.92(23.22%) |
| Southern Latin America | Both | 1880.09 | 1952.64(103.86%) | 6384.94(339.61%) | -6457.48(-343.47%) |
| Southern Sub-Saharan Africa | Both | 18467.72 | 6899.93(37.36%) | 13587.23(73.57%) | -2019.44(-10.93%) |
| Tropical Latin America | Both | 48603.09 | 14405.37(29.64%) | 21893.71(45.05%) | 12304.01(25.32%) |
| Western Europe | Both | -75049.73 | 13910.21(-18.53%) | -13367.64(17.81%) | -75592.31(100.72%) |
| Western Sub-Saharan Africa | Both | 111769.13 | -328.95(-0.29%) | 83713.48(74.9%) | 28384.6(25.4%) |

DALYs: disability-adjusted life years; SDI: sociodemographic index.

**Table S9**. Incident cases, deaths and DALYs projections of adolescents and young adults breast cancer across different regions in 2044.

| **Location** | **Sex** | **Incident cases** | **Deaths** | **DALYs** |
| --- | --- | --- | --- | --- |
| **Global** | Both | 244018 | 54202 | 3224966 |
| **Andean Latin America** | Both | 1500 | 316 | 18608 |
| **Australasia** | Both | 1041 | 108 | 6653 |
| **Caribbean** | Both | 1134 | 313 | 17926 |
| **Central Asia** | Both | 1906 | 516 | 29102 |
| **Central Europe** | Both | 2278 | 326 | 19203 |
| **Central Latin America** | Both | 12043 | 1729 | 103766 |
| **Central Sub-Saharan Africa** | Both | 3963 | 1719 | 99294 |
| **East Asia** | Both | 29859 | 3136 | 217585 |
| **Eastern Europe** | Both | 3278 | 666 | 37139 |
| **Eastern Sub-Saharan Africa** | Both | 18939 | 7779 | 453616 |
| **High-income Asia Pacific** | Both | 3517 | 368 | 21207 |
| **High-income North America** | Both | 12880 | 1465 | 92317 |
| **North Africa and Middle East** | Both | 27160 | 3737 | 229829 |
| **Oceania** | Both | 567 | 226 | 14359 |
| **South Asia** | Both | 48318 | 16052 | 944402 |
| **Southeast Asia** | Both | 19974 | 5427 | 316472 |
| **Southern Latin America** | Both | 1915 | 399 | 22854 |
| **Southern Sub-Saharan Africa** | Both | 2108 | 1028 | 56582 |
| **Tropical Latin America** | Both | 6741 | 1353 | 83952 |
| **Western Europe** | Both | 12723 | 1301 | 81245 |
| **Western Sub-Saharan Africa** | Both | 15262 | 6127 | 353938 |

DALYs: disability-adjusted life years.
